# Phosphorylation-dependent control of transcription factor activity regulates temporal patterning of cortical cell fate specification

**DOI:** 10.1038/s41467-026-77195-8

**Published:** 2026-09-04

**Authors:** Arun Mahesh, Anuj Kumar Dwivedi, Xuan Wang, Souren Sadhukhan, Dima Amairy, Hanife Pekel, Özge Şensoy, Martin R. Larsen, Vijay K. Tiwari

**Affiliations:** 1 Institute for Molecular Medicine, University of Southern Denmark, Odense M, Denmark; 2 Graduate School of Engineering and Natural Sciences, Istanbul Medipol University, Istanbul, Turkey; 3 Regenerative and Restorative Medicine Research Center (REMER), Research Institute for Health Sciences and Technologies (SABITA), Istanbul Medipol University, Istanbul, Turkey; 4 Department of Pharmacy Services, Vocational School of Health Services, İstanbul Medipol University, İstanbul, Turkey; 5 Department of Biochemistry and Molecular Biology, University of Southern Denmark, Odense, Denmark; 6 Danish Institute for Advanced Study (DIAS), Odense M, Denmark; 7 Department of Clinical Genetics, Odense University Hospital, Odense C, Denmark; 8 Mohamed bin Zayed University of Artificial Intelligence, Abu Dhabi, UAE; 9 Wellcome-Wolfson Institute for Experimental Medicine, School of Medicine, Dentistry, and Biomedical Science, Queens University Belfast, Belfast, UK

**Keywords:** Developmental biology, Neuroscience, Computational biology and bioinformatics

## Abstract

The development of the cerebral cortex requires precise temporal control of transcription factor (TF) activity to coordinate neuronal and glial lineage transitions. However, the molecular mechanisms coupling signaling dynamics to TF function remain unclear. Here, we combine proteomic, phosphoproteomic, and multi-omic analyses of the developing mouse cortex to define phosphorylation as a key post translational mechanism governing cortical cell fate decisions. Across the neurogenic to gliogenic transition, we identify a global remodeling of the phosphoproteome, including extensive phosphorylation of distinct TFs that is linked to their developmental-stage and cell-type-specific gene regulatory activity. Among these, Hmgn3 and Nfib emerge as pivotal regulators acting in sequential developmental phases. Phosphorylation of Hmgn3 at S78 enhances its chromatin association and activation of progenitor related genes, promoting progenitor proliferation. In contrast, phosphorylation of Nfib at S265 strengthens DNA binding at distal enhancers, driving axonogenesis and neuronal maturation. In utero perturbations with wild-type and phospho-mutant TFs combined with single-cell transcriptomic analyses reveal corresponding shifts in cell-type proportions. Our findings establish site-specific phosphorylation as a molecular timer that orchestrates TF activity to control the temporal patterning of cortical fate specification and ensure the precise assembly of cortical circuits.

## Abstract (web summary)

Here, researchers reveal how chemical tags on two proteins act as a molecular timer during brain development, guiding stem cells to first multiply, then mature into neurons—offering new insight into how the cerebral cortex is built.

## Introduction

Brain development is a highly intricate and tightly regulated process that involves the timely generation and differentiation of various cell types, ultimately leading to the formation of the cerebral cortex. This complex journey is orchestrated through the synergy of multiple signaling pathways, the precise expression of pioneer transcription factors, and epigenetic mechanisms that drive the differentiation of neural stem cells into diverse cell types. Such processes are essential for higher cognitive functions and are conserved across species, including humans and mice^1,2^. Although there are differences in developmental timelines and cell population proportions among species, the fundamental genetic and molecular pathways governing cortical development are largely shared^3,4^. However, the interplay between phosphorylation signaling and gene expression programs remains poorly understood.

Several signaling pathways, including Wnt^5^, Sonic Hedgehog (Shh)^6^, Fibroblast Growth Factor (FGF)^7^, and Notch^8^, have been implicated in regulating neural progenitor proliferation and differentiation. They impact cellular function through post-translational modifications (PTMs) of downstream proteins, influencing their activity, stability, and interactions. These modifications include phosphorylation, ubiquitination, glycosylation, methylation, and more, each contributing to the complexity of cellular functions. Among these, phosphorylation is particularly significant in cellular signaling, including in both neurogenesis and gliogenesis^9,10^, impacting cell identity, differentiation, and maturation. Increasing evidence suggests that such phosphorylation cascades function to either enhance or inhibit the activity of specific transcription factors (TFs) to influence underlying gene expression programs. For instance, phosphorylation of STAT3 (Y705)^11^, Ngn2 (S231/S234)^12,13^ and Sox2 (S39)^14^ has been shown to influence neural stem cell fate, while the phosphorylation of OLIG2 (S147)^15^ is known to promote glial differentiation.

Temporal patterning refers to developmental stage-specific progression through which cortical progenitors transition across competence states to generate distinct neuronal and glial populations. This is distinct from developmental rate or duration, which describes how fast development proceeds. Temporal identity transitions have been extensively studied in Drosophila neuroblasts, where sequential transcription factor programs define lineage progression^16,17^. In both invertebrate and vertebrate systems, phosphorylation is emerging as a critical regulator of pro- neural factor activity, such as Neurogenin^12,18–20^ and Ascl1^21,22^, modulating differentiation competence and timing.

Despite major advances in developmental neurobiology, how phosphorylation regulates the spatiotemporal control of transcription factor (TF) activity during cortical development remains insufficiently understood^12,16–22^. In this study, we apply an integrated multi-omics framework to dissect the phosphoproteomic landscape of the developing mouse cortex, focusing on the neurogenic (E14.5) and gliogenic (E18.5) stages. We identify distinct patterns of protein expression and phosphorylation dynamics, including key TFs that correlate with key developmental transitions. Among these, we pinpoint Hmgn3 and Nfib as pivotal TFs regulating progenitor maintenance and neuronal differentiation, respectively. Combining in utero brain perturbation with single-cell transcriptomic (scRNA-seq) and genome-wide TF-binding (CUT&RUN) analyses, we demonstrate that site-specific phosphorylation alters their genomic occupancy and functional output. We showed phosphorylated Hmgn3 promotes progenitor proliferation, whereas phosphorylated Nfib drives axonogenesis and neuronal maturation. Together, these findings reveal phosphorylation as a fundamental regulatory layer coordinating TF activity across developmental stages, thereby refining our understanding of how molecular signaling establishes the cellular diversity of the cerebral cortex.

## Results

### A phospho-regulatory switch during the neurogenic-to-gliogenic transition in cortical development

**Fig. 1:**
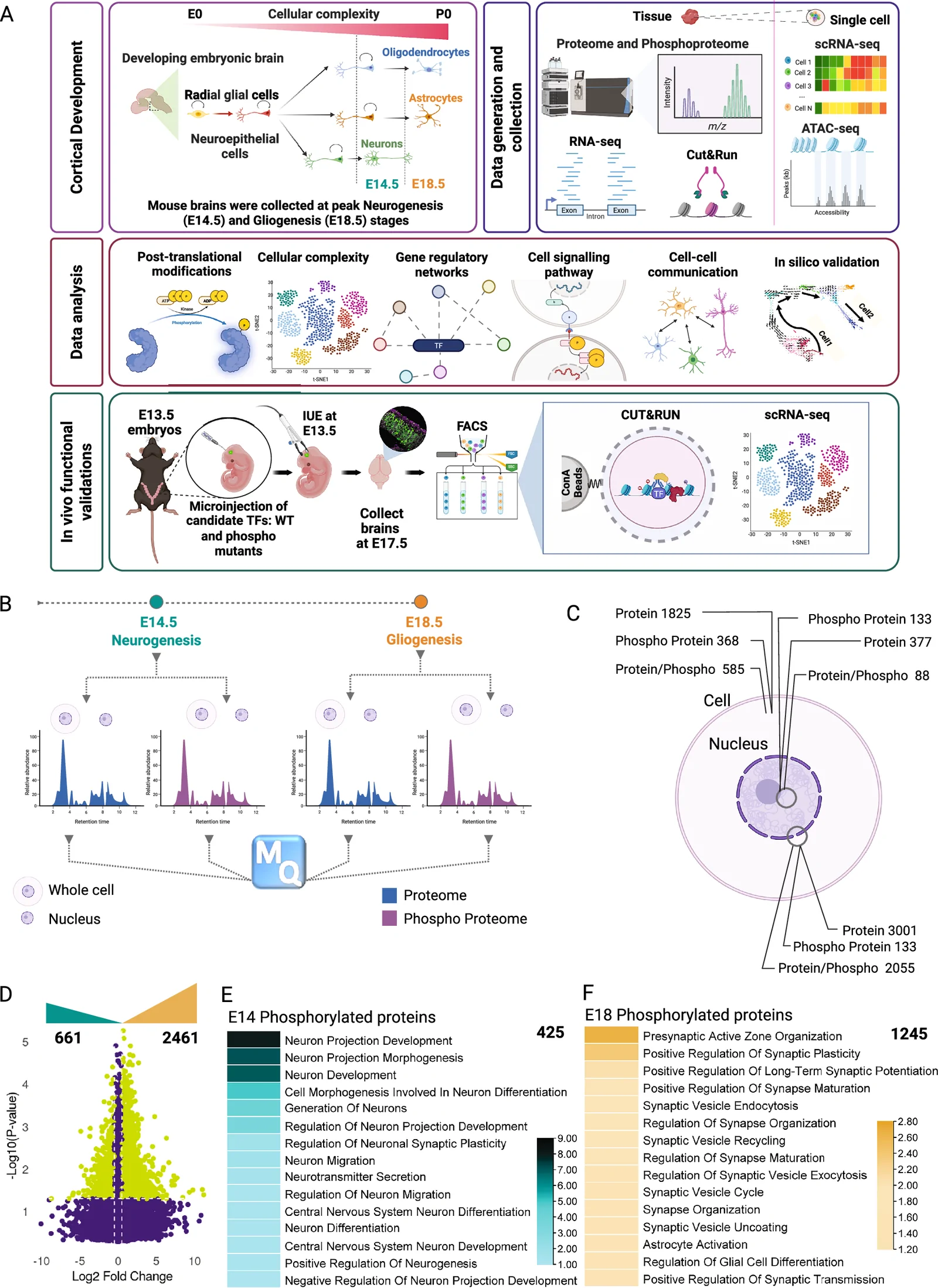
Phosphoproteome complexity increases during the neurogenic‑to‑gliogenic transition. **A** Overview of the integrated multi‑omics experimental design combining whole‑cell and nuclear proteomics, phosphoproteomics, bulk RNA‑seq, single‑cell RNA‑seq, and CUT&RUN from mouse cortex at E14.5 (neurogenic peak) and E18.5 (onset of gliogenesis). **B** Proteomic and phosphoproteomic profiles across stages. **C** Quantification of proteins and phosphoproteins detected in cytoplasmic, nuclear, and shared compartments (*n* = 3). **D** Volcano plot showing differentially phosphorylated proteins between E14.5 and E18.5, revealing a global increase in phosphorylation at later stages (*n* = 3, *P* ≤ 0.05, localization probability ≥ 0.8 and log2 fold change ± 0.5). **E**,** F** GO enrichment of hyper phosphorylated proteins at E14.5 (**E**) and E18.5 (**F**), showing neurogenesis related enrichment at E14.5 and gliogenesis related pathways at E18.5. Created in BioRender. Lusby, R. (2026) https://BioRender.com/w1b4a8x.

To delineate the molecular programs governing the transition from neurogenesis to gliogenesis, we generated a comprehensive multi-omic atlas of the developing mouse cortex at embryonic day 14.5 (E14.5) and E18.5 (Fig. 1A). These timepoints capture the peak of neurogenesis and the onset of gliogenesis, respectively. Our analysis integrates whole-cell and nuclear proteomics and phosphoproteomics with bulk RNA-Seq, CUT&RUN, and single-cell RNA-Seq to create a holistic view of cortical maturation, from transcription and chromatin state to protein expression and post-translational modification (Fig. 1A).

To define the post-translational landscape of this critical developmental window, we first performed in-depth comparative proteomic and phosphoproteomic profiling of the E14.5 and E18.5 cortex at both whole-cell and nuclear levels (Fig. 1B). This subcellular resolution allowed us to distinguish nuclear, cytoplasmic, and shared proteomes (Fig. 1C). These cortex-derived proteomics datasets demonstrated high reproducibility across biological replicates, confirming the robustness of distinct proteomic and phosphoproteomic profiles at each stage (Supplementary Fig. 1A–D).

Differential analysis revealed a dynamic post-translational landscape. We observed a significant increase in the number of hyper-phosphorylated proteins at E18.5 compared to E14.5 (Fig. 1D and Supplementary Data 1), a change that coincides with increasing cellular diversity and the start of gliogenesis. Gene ontology (GO) enrichment of differentially enriched phosphoproteins demonstrated a clear stage-specific functional divergence. At E14.5, hyper-phosphorylated proteins were predominantly associated with neurogenesis, neuron migration and neuronal projection (Fig. 1E). In contrast, by E18.5, the phosphoproteome was significantly enriched for biological processes related to neuron maturation, plasticity and regulation of glial cell differentiation (Fig. 1F). These data reveal extensive phosphoproteome remodeling during cortical development, with stage-specific phosphorylation patterns corresponding to distinct developmental programs of neurogenesis at E14.5 and gliogenesis at E18.5. Notably, our phosphoproteomic design captures differences between two key developmental states and reflects stage-associated regulatory states rather than dynamic trajectories.

### Phospho-control of transcription factor activity defines cell identity during cortical development

**Fig. 2:**
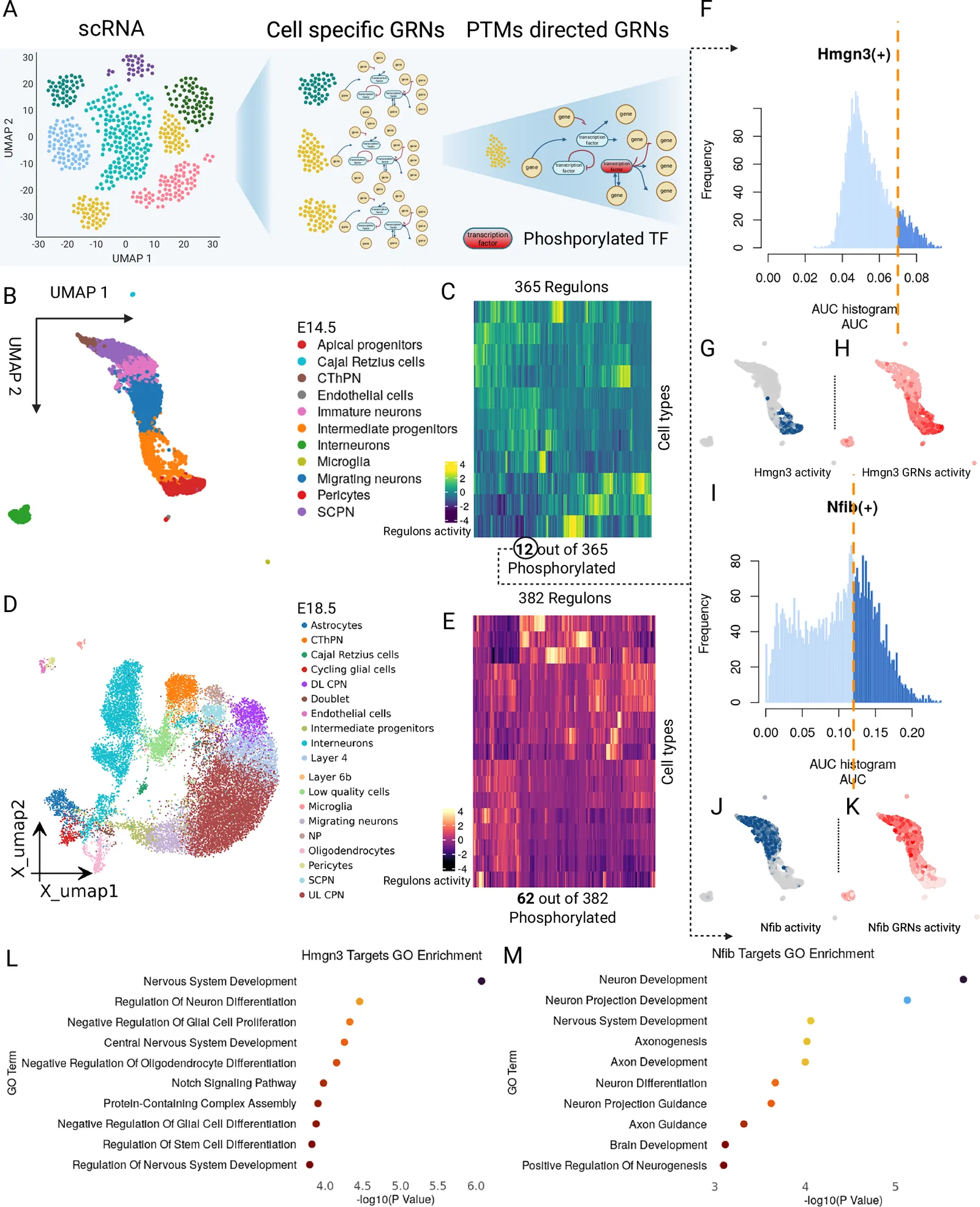
Phospho‑regulation of transcription factor networks defines cell identity during cortical development. **A** Schematic illustrating the integration of phosphoproteomic data with scRNA‑seq‑derived gene regulatory networks (GRNs). **B**,** D** UMAP representations of cell clusters at E14.5 (**B**) and E18.5 (**D**) annotated by cell type. **C**,** E** Heatmaps showing top regulons per cell type, revealing a > 5‑fold increase in differentially phosphorylated TFs-from 12 (E14.5) to 62 (E18.5). **F**–**H** Hmgn3 regulon activity and target expression, highlighting its enrichment in progenitors. **I**–**K** Nfib regulon activity and target expression marking neuronal populations. **L**,** M** GO enrichment (*p*-value ≤ 0.05) analyses of corresponding target genes of Nfib and Hmgn3. Created in BioRender. Lusby, R. (2026) https://BioRender.com/akf4d7k.

To identify transcriptional regulators of cell identity, we constructed cell-type-specific gene regulatory networks (GRNs) for the neurogenic (E14.5) and gliogenic (E18.5) stages of cortical development (Fig. 2A). Single-cell RNA-seq analysis resolved distinct populations of progenitors, neurons, and glia at each stage (Fig. 2B, D and Supplementary Fig. 2A, B). At E14.5, the GRNs of apical progenitors and migrating neurons were driven by canonical neurogenic regulons such as Hes5 and Neurod1. In contrast, the E18.5 network was characterized by the activity of gliogenic regulons like Olig2 and Stat1, alongside those defining mature neuronal subtypes (e.g., Rorb, Dlx1) (Supplementary Figs. 3–7). Integration of phosphoproteomic data with GRN analysis revealed that a subset of these transcription factors exhibits stage-associated phosphorylation changes. We observed a more than five-fold increase in the number of differentially phosphorylated TFs between the two stages, from 12 (out of 365) at E14.5 to 62 (out of 382) at E18.5 (Fig. 2C, E), indicating an expansion of phospho-regulatory modulation during the transition to gliogenesis.

To investigate the functional relevance of phosphorylation-dependent regulation, we focused on two representative transcription factors, Hmgn3 and Nfib, selected based on high-confidence phosphoproteomic detection, strong gene regulatory network activity, and complementary developmental expression patterns (Supplementary Fig. 1H, I, and Supplementary Figs. 8A, 9A–E). At E14.5, Hmgn3 activity was highest in apical and intermediate progenitors, and its target genes were significantly enriched for stem cell maintenance and differentiation pathways (Fig. 2F–H, L and Supplementary Fig. 8B). In contrast, Nfib activity was preferentially associated with differentiating and mature neuronal populations at E14.5, with its target genes controlling neuronal projection and synaptic development (Fig. 2I–K, M and Supplementary Fig. 8C). Together, these observations suggested that stage-associated phosphorylation of a transcription factors play important role for phosphorylation-dependent gene regulatory programs, which defines cell identity during cortical development.

### Phosphorylation-associated rewiring of developmental gene expression programs

**Fig. 3:**
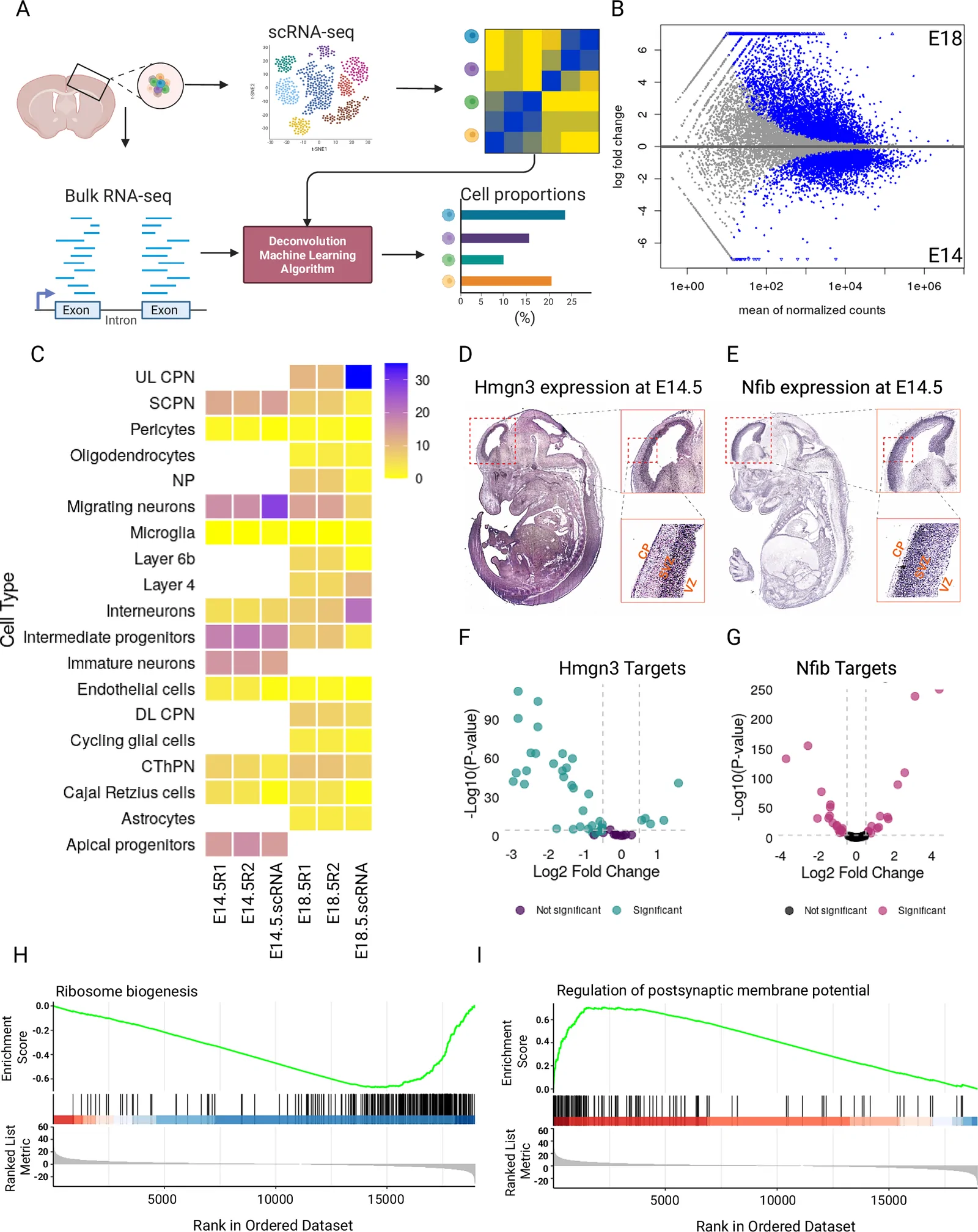
Transcriptomic analysis validates a functional switch between Hmgn3 and Nfib. **A** Workflow for bulk RNA‑seq and deconvolution analysis of E14.5 and E18.5 cortices. **B** MA plot showing differentially expressed genes (DEGs) between stages. **C** Heatmap depicting cell‑type composition inferred from bulk data matched with scRNA‑seq reference. **D**,** E** In situ hybridization images (GenePaint Atlas) show Hmgn3 localized to VZ/SVZ and Nfib enriched in CP neurons. **F**,** G** Volcano plots illustrating E18.5 downregulation (Wilcoxon rank-sum test, padj < 0.05, log2FoldChange < − 0.5) of Hmgn3 target genes (**F**) and upregulation (Wilcoxon rank-sum test, padj < 0.05, log2FoldChang > 0.5) of Nfib target genes (**G**). **H**,** I** GSEA revealing downregulation of ribosome biogenesis pathways and upregulation of postsynaptic signaling at E18.5. Created in BioRender. Lusby, R. (2026) https://BioRender.com/y3fhl2q.

To validate the downstream consequences of this phosphorylation-associated regulatory mechanism, we performed bulk RNA-sequencing on the matched embryonic cortical tissues used in the proteomic analysis (Fig. 3A). Differential gene expression (DEG) analysis identified stage-specific transcriptional changes between E14.5 and E18.5 (Fig. 3B). The resulting transcriptomic dataset (Supplementary Fig. 1E–G) was deconvoluted to confirm concordant cell-type proportions with our single-cell analysis (Fig. 3C). We focused on the two representative TFs identified through GRN analysis, Hmgn3, active in E14.5 progenitors, and Nfib, active in E14.5 neurons (Fig. 2F–M and Supplementary Fig. 8). In situ expression analysis of E14.5 mouse brain validated the distinct spatial expression patterns for Hmgn3 and Nfib. Hmgn3 was enriched in the progenitor-rich ventricular and sub-ventricular zones (VZ/SVZ) (Fig. 3D), Nfib exhibited a clear developmental gradient, with low expression in the VZ and strong expression in differentiating neurons throughout the deep and upper layers of the cortical plate (CP) (Fig. 3E and Supplementary Fig. 10A). Importantly, phosphorylation did not detectably alter the subcellular localization of Hmgn3 and Nfib (Supplementary Fig. 10B, C). In line with stage-associated GRN activity, the target genes of Hmgn3 were significantly downregulated at E18.5, while the targets of Nfib were concurrently upregulated (Fig. 3F, G). This stage-specific, phosphorylation-driven exchange in TF dominance corresponds to a global functional shift in the cortical transcriptome. Gene Set Enrichment Analysis (GSEA) of the bulk RNA-Seq data revealed downregulation of pathways related to ribosome biogenesis, a hallmark of proliferating progenitors^23^ and upregulation of pathways governing postsynaptic membrane potential, essential for maturing neuronal function (Figs. 3H, I and Supplementary Fig. 1F).

Altogether, these findings indicate that stage-associated phosphorylation of key transcription factors acts as a critical regulatory layer, by coordinated rewiring of gene expression programs during cortical development.

### Phosphorylation-Dependent Regulation of Hmgn3 and Nfib Across Cortical Differentiation Stages

From the broader set of phosphorylated transcription factors identified in our dataset (Supplementary Fig. 9A, B), we prioritized Hmgn3 and Nfib as representative candidates using a systematic framework integrating phosphoproteomic confidence, GRN activity, developmental expression, and experimental feasibility. Both factors exhibited high-confidence phosphorylation (localization probability ≥ 0.8) with significant stage-associated differences between neurogenic (E14.5) and gliogenic (E18.5) stages, and together represent complementary developmental contexts progenitor maintenance and neuronal maturation, respectively enabling assessment of phosphorylation-dependent regulation across the full cortical lineage trajectory.

**Fig. 4:**
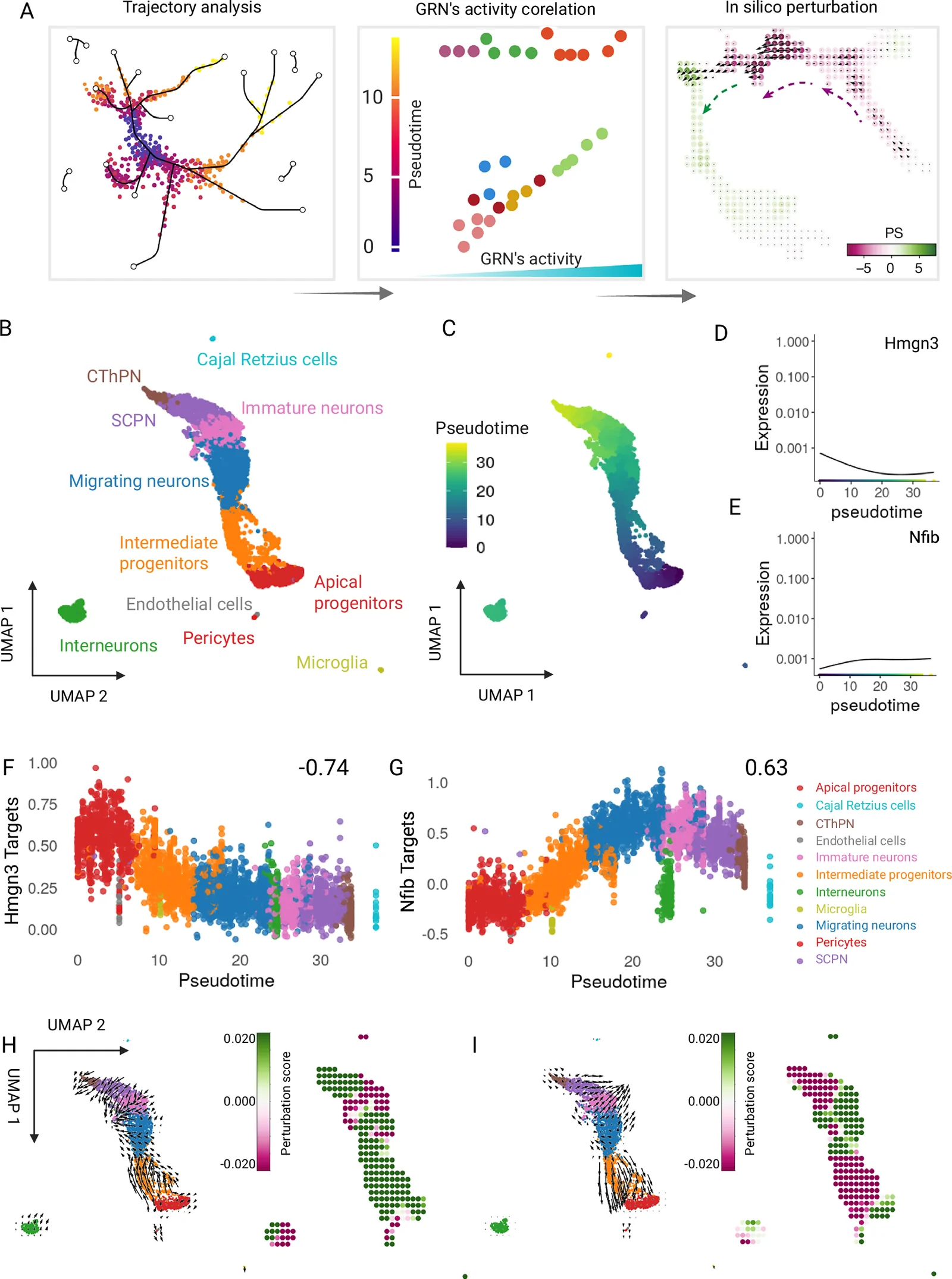
Developmental regulation of Hmgn3 and Nfib regulon activity during cortical differentiation. **A** Schematic of single‑cell trajectory and perturbation analysis. **B**,** C** UMAP visualization of cell‑type identities (**B**) and developmental pseudotime (**C**) from progenitors to mature neurons. **D**,** E** Expression dynamics of Hmgn3 (**D**) and Nfib (**E**), showing temporally distinct activation patterns. **F**,** G** Correlation of Hmgn3 (**F**) and Nfib (**G**) module activity with pseudotime, confirming early Hmgn3 and late Nfib engagement. **H**,** I** In silico perturbation predictions: Hmgn3 loss impairs early differentiation (**H**), while Nfib perturbation disrupts late neuronal maturation (**I**). Created in BioRender. Lusby, R. (2026) https://BioRender.com/8r5g0ws.

Ordering single cells along pseudotime from neural progenitors to mature neurons (Fig. 4A–C) revealed a coordinated stage-associated transition between the two factors. The Hmgn3 GRN was highly active in early progenitor populations but declined progressively as differentiation advanced (Fig. 4D, F), whereas the Nfib GRN became increasingly activated as Hmgn3 activity diminished, with peak activity coinciding with neuronal migration and maturation (Fig. 4E, G and Supplementary Fig. 11). This complementary dynamic was further supported by strong, stage-specific regulon enrichment in scRNA-seq–derived GRNs (Fig. 4C and Supplementary Fig. 9C, D).

In silico perturbation analyses reinforced distinct functional roles within the differentiation trajectory (Supplementary Fig. 12A–B). Suppression of Hmgn3 increased neurogenic output, consistent with premature loss of progenitor identity (Fig. 4H), while disruption of Nfib selectively impaired intermediate progenitor populations and reduced the efficiency of maturation among migrating and cortical projection neurons (Fig. 4I).

We next asked whether phosphorylation contributes mechanistically to these activity differences. The phosphosites S78 (Hmgn3) and S265 (Nfib) reside within highly conserved domains, where in silico mutagenesis predicts distinct effects on protein stability and function (Supplementary Figs. 13–14). Notably, residues flanking Nfib S265 overlap with known variant hotspots for human neurodevelopmental disorders (Supplementary Fig. 13D). Kinase prediction further identified divergent upstream regulators CDK/NLK families for Nfib and GRK/PRPK families for Hmgn3 suggesting that stage-specific kinase activities contribute to the differential regulation of these transcription factors during cortical neurogenesis (Supplementary Figs. 15–16).

### Phosphorylation at S78 regulates Hmgn3 chromatin association and progenitor-specific function

**Fig. 5:**
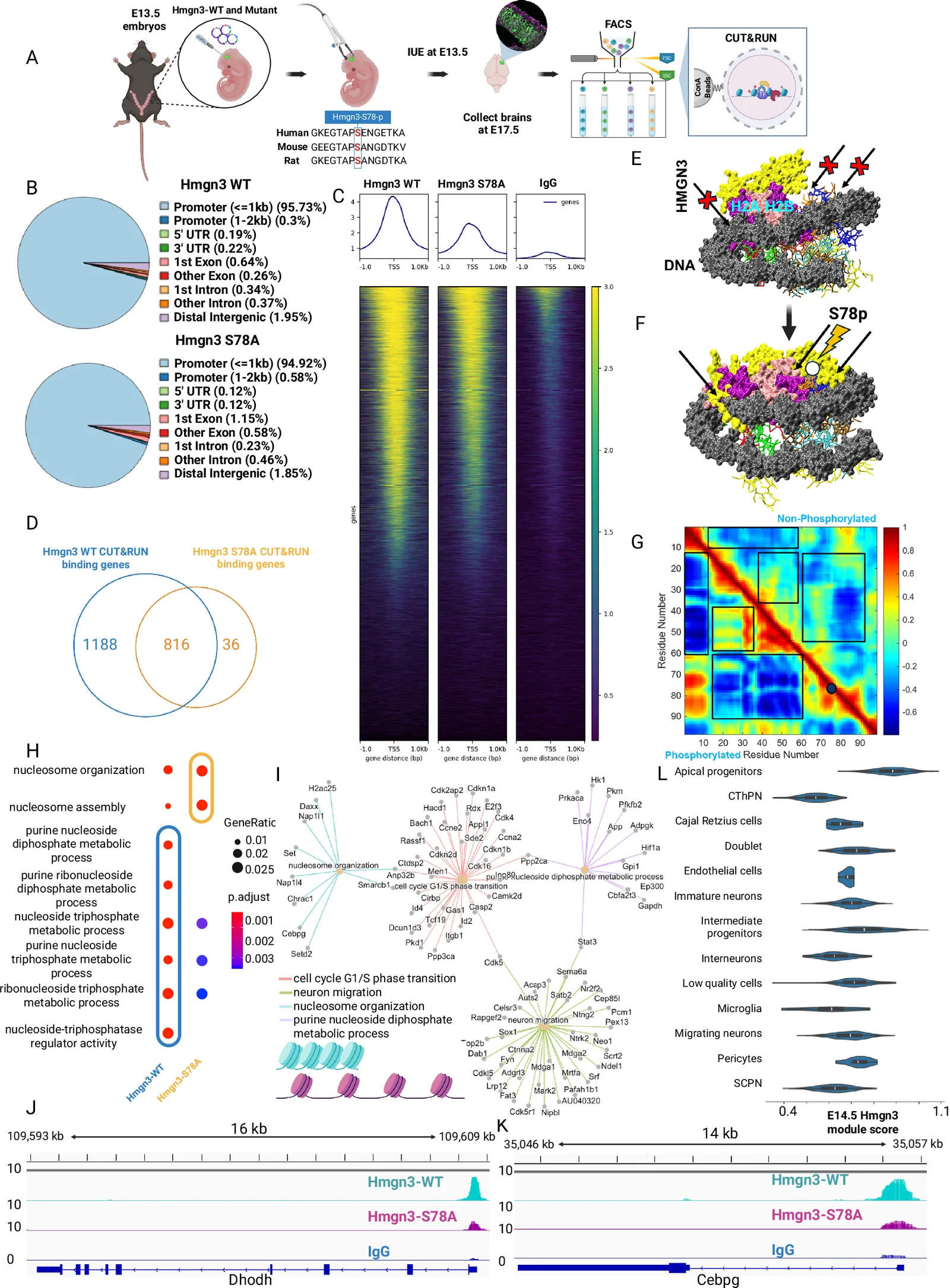
Phosphorylation at S78 is essential for Hmgn3 chromatin binding and progenitor‑state maintenance. **A** Schematic of in utero electroporation (IUE) and CUT&RUN for Hmgn3 WT (*n* = 2) and phospho-deficient Hmgn3 S78A (*n* = 2). **B**–**D** Genome‑wide occupancy analysis showing markedly reduced peaks (*q*-value < 0.05) for S78A mutant, indicating phosphorylation‑dependent chromatin engagement. **E**,** F** The representative conformation of non-phosphorylated and phosphorylated Hmgn3 complex with the nucleosome modeled using the crystal structure of HMGN2/nucleosome complex (PDB ID: 9D3M). The parts of Hmgn3 that contact with histones and DNA are indicated with arrows in the phosphorylated system, whereas those that do not contact are indicated with red crosses. The Hmgn3 and the nucleosome are shown in surface representation and yellow and gray, respectively. The phosphorylated residue is shown by a white ball. Each histone protein is shown with a different color. **G** Cross‑correlation matrices comparing the dynamic behavior of phosphorylated vs. unphosphorylated Hmgn3. **H**,** I** GO analyses (abs(log2FoldChange) > 1 & padj < 0.05) show Hmgn3‑WT targets enriched for nucleotide metabolism and nucleosome organization; these diminish in S78A. **J**,** K** Examples of differential peak occupancy at representative targets. (L) Combined CUT&RUN and E14.5 scRNA-seq analyses (*n* = 5249 cells) demonstrate significant enrichment of Hmgn3 activity in apical progenitors. Created in BioRender. Lusby, R. (2026) https://BioRender.com/3a7wmw2.

To determine whether Hmgn3 function in neural progenitors depends on its DNA-binding activity and whether this association is modulated by phosphorylation, we performed genome-wide binding analyses in developing mouse cortex using CUT&RUN following expression of either wild-type (WT) Hmgn3 or a phospho-deficient mutant S78A (Fig. 5A). WT Hmgn3 exhibited broad genomic occupancy, with 2671 peaks linked to 2006 genes, whereas the S78A mutant bound substantially fewer loci (866 peaks associated with 852 genes), indicating that S78 phosphorylation is critical for stable chromatin association (Fig. 5B–D). Despite this dramatic reduction, both WT and mutant proteins preferentially targeted promoter-proximal regions ( < 1 kb from transcription start sites) (Fig. 5B and Supplementary Fig. 17A–B), suggesting that phosphorylation influences Hmgn3’s engagement strength rather than its genomic binding preference (Supplementary Data 2–6).

Hmgn3 directly binds nucleosomes and facilitates chromatin decompaction to promote transcriptional activation^24^. To elucidate how phosphorylation modulates this interaction, we performed coarse-grained molecular dynamics (CG-MD) simulations based on the Hmgn3–nucleosome crystal structure (PDB ID: 9D3M). Our model, containing two Hmgn3 molecules bound to opposing nucleosome faces, revealed that in both phosphorylated and unphosphorylated states, Hmgn3 anchors through residues R25–R29, interacting with the acidic patch of H2A–H2B (Fig. 5E, F). However, the phosphorylated form displayed fewer histone–DNA contacts (375 ± 60 vs. 618 ± 95) and tighter Hmgn3–nucleosome interactions (381 ± 33 vs. 305 ± 32) (Fig. 5G and Supplementary Fig. 17C). Moreover, phosphorylation modulated correlations between the N-terminal and middle/distal site, as well as between the middle and the distal phosphorylation site, S78, as revealed by dynamics cross-correlation analyses (Fig. 5G). Together, these simulations support a model in which phosphorylated Hmgn3 makes stronger contacts with the nucleosome, destabilizes histone–DNA interactions, leading to a more transcription-permissive chromatin state. To assess the biological impact of this regulation, we analyzed Gene Ontology (GO) terms associated with WT and mutant Hmgn3 target genes (Supplementary Data 2–6). WT Hmgn3–bound genes were highly enriched for nucleotide metabolism and nucleosome organization pathways, whereas these enrichments were markedly reduced in the S78A mutant (Fig. 5H–I). For example, representative targets such as Cebpg and Dhodh, implicated in nucleosome organization and purine metabolism, respectively, showed stronger WT binding compared with the mutant (Fig. 5J, K). Integrative analysis with E14.5 cortical scRNA-seq data revealed that Hmgn3 target genes were most enriched in apical progenitors, supporting its role in maintaining early progenitor identity (Fig. 2F–H; 5 L).

Collectively, these results establish that site-specific phosphorylation of Hmgn3 at S78 is a key regulatory mechanism that reinforces its chromatin occupancy and facilitates the transcriptional programs sustaining progenitor states during cortical development.

### Nfib’s Phosphorylation at S265 enhances its chromatin binding and drives neuronal maturation programs

**Fig. 6:**
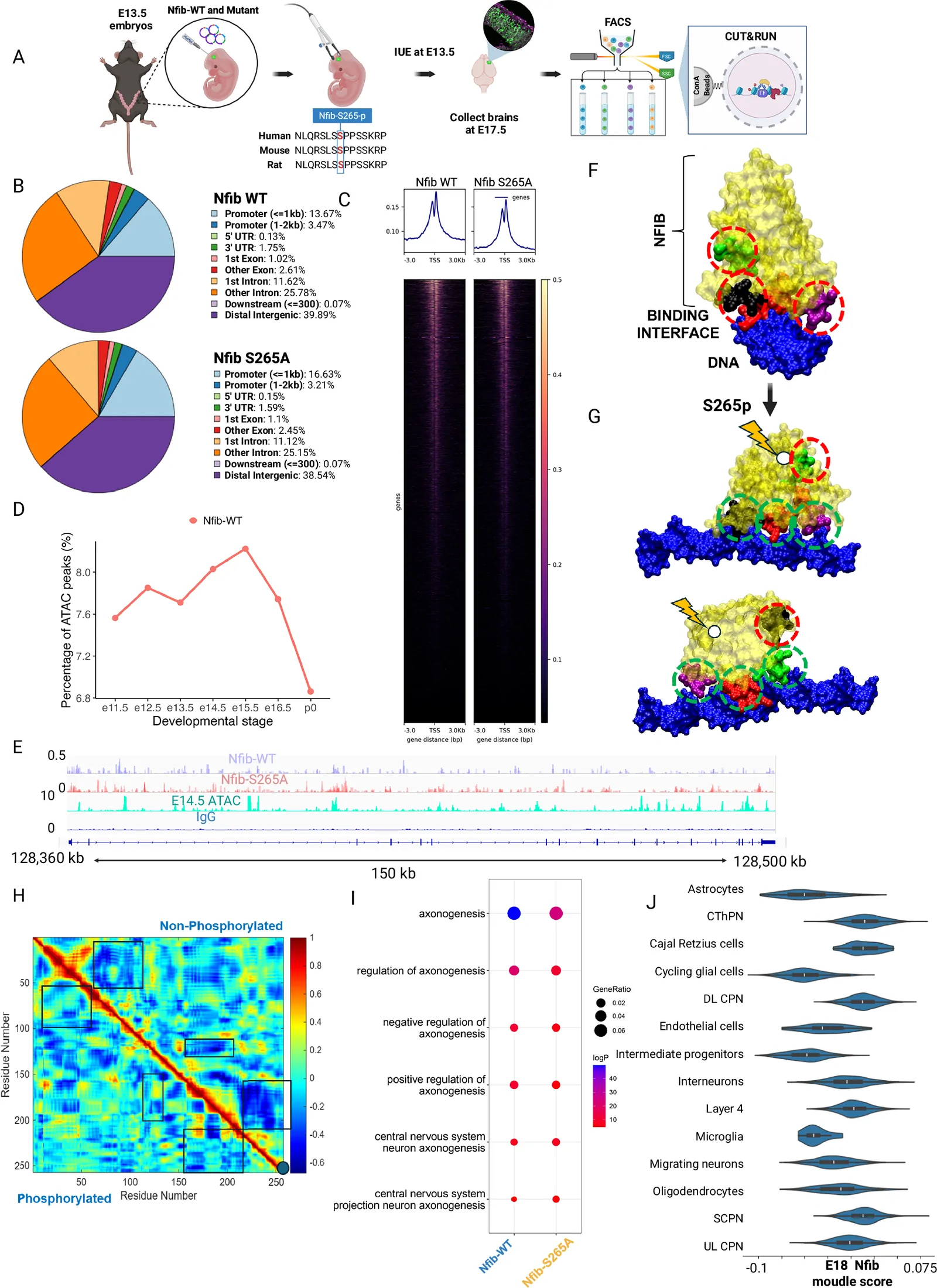
Phosphorylation at S265 enhances Nfib chromatin binding and drives neuronal maturation. **A** Experimental workflow for IUE–CUT&RUN of NfibWT (*n* = 2) and phospho deficient Nfib S265A (*n* = 2) constructs. **B**,** C** Comparison of genomic binding sites (*q*-value < 0.05), showing stronger occupancy for WT Nfib. **D**,** E** Integration with time‑course forebrain ATAC‑seq (*q*-value < 0.05) reveals Nfib binding regions gaining accessibility around E14.5–E15.5. **F**, **G** The representative conformation of the non-phosphorylated and phosphorylated Nfib complex with DNA. Regions that do not contact with DNA are circled with red dashed lines, whereas those that contact with DNA are circled with green dashed lines. NFIB and DNA are shown in surface representation and in yellow and blue, respectively. The phosphorylated residue is shown by a white ball. The interface region is determined based on the crystal structure of NF1-X/DNA complex (PDB ID:9QKY). **H** Dynamic cross‑correlation comparison shows long‑range allosteric effects of S265 phosphorylation stabilizing DNA interactions. **I** GO enrichment with DEGs (Wilcoxon rank-sum test, padj < 0.05), indicating WT Nfib targets axonogenesis and neuron differentiation pathways. **J** scRNA-seq integration reveals preferential expression of Nfib targets in neuronal lineages at E18.5 (*n* = 20,275 cells). Created in BioRender. Lusby, R. (2026) https://BioRender.com/u7ohyvt.

Prompted by the findings above, we next examined whether phosphorylation modulates Nfib function during cortical development. We performed genome-wide binding profiling of Flag-tagged wild-type (WT) Nfib and its phospho-deficient mutant (S265A) using CUT&RUN following in utero expression (Fig. 6A). WT Nfib bound to a substantially greater number of genomic loci (5753 peaks, 2433 genes) compared to the S265A mutant (3232 peaks, 1196 genes), indicating that S265 phosphorylation enhances chromatin occupancy (Fig. 6B, C). In contrast to Hmgn3, a large fraction of Nfib targets were located at distal regulatory elements, but no major shift in genomic binding preference was observed between WT and mutant forms (Fig. 6B, C, Supplementary Fig. 18A and Supplementary Data 7–10). Integration with time-course forebrain ATAC-seq data (E11.5–P0) revealed that Nfib-bound elements exhibited maximal chromatin accessibility at E14.5–E15.5, coinciding with peak neurogenesis, and declined thereafter (Fig. 6D, E). These observations suggest that while phosphorylation does not alter target specificity, it amplifies Nfib binding capacity at developmentally active distal enhancers.

To investigate how phosphorylation influences Nfib–DNA complex formation, we conducted coarse-grained molecular dynamics (CG-MD) simulations using the interface residues revealed by the Nfix–DNA crystal structure (PDB ID: 9QKY) to monitor interactions at the binding interface of Nfib/DNA. The non-phosphorylated complex displayed a single dominant interaction region (residues D110–W128), whereas the phosphorylated complex exhibited three stable contact regions: D110–W128 and A78–R85 (red and purple) maintained across three replicates of the simulations, and a flexible third contact alternating between R39–H45 and K139–R150 (black and green) (Fig. 6F). These conformational changes are consistent with enhanced DNA binding observed experimentally (Fig. 6G-E and Supplementary Fig. 18C). Although S265 lies outside the DNA-binding interface, phosphorylation induced notable long-range allosteric effects. Dynamic cross-correlation analyses revealed that phosphorylation reduced negative correlations between the S265 and DNA-contact residues as well as among three contact regions, allowing formation of additional stable interactions within the complex (Fig. 6H).

Gene Ontology (GO) analysis of WT Nfib–bound genes highlighted enrichment in axonogenesis and neuronal differentiation pathways, which was markedly diminished in the S265A mutant (Fig. 6I). Integrating E14.5 cortical scRNA-seq data with Nfib targets revealed that neuronal populations were selectively enriched for Nfib-bound genes, whereas glial lineages showed minimal association patterns consistent with earlier analyses (Figs. 2I–K, 6J).

Collectively, these results establish that phosphorylation of Nfib at S265 is a key regulatory event that enhances DNA binding through allosteric modulation and strengthens occupancy at developmentally active distal enhancers, thereby driving the transcriptional programs underlying neuronal maturation during cortical development.

### Phosphorylation-dependent regulation of Hmgn3 and Nfib directs divergent gene expression programs underlying cortical cell-fate decisions

**Fig. 7:**
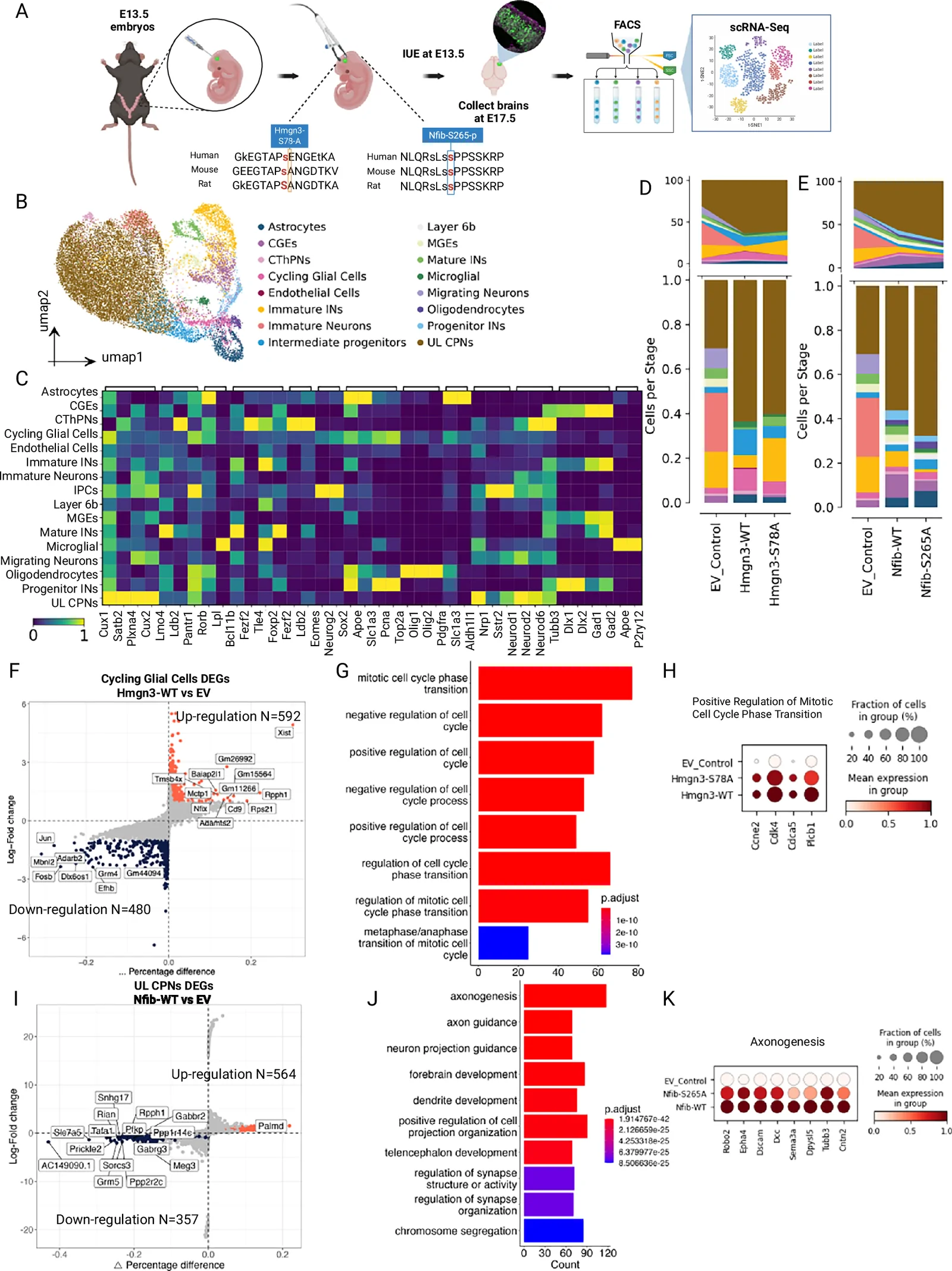
Single‑cell transcriptomics reveal phosphorylation‑dependent lineage changes driven by Hmgn3 and Nfib. **A** Overview of IUE–scRNA‑seq design with EV, Hmgn3‑WT, Hmgn3‑S78A, Nfib‑WT, and Nfib‑S265A conditions. **B**, **C** UMAP clustering and marker‑based cell‑type annotations across all conditions. **D**,** E** Stacked bar plots showing lineage proportion shifts induced by Hmgn3 (**D**) and Nfib (**E**) overexpression and mutation. **F**–**H** Differentially expressed genes (DEGs, Wilcoxon rank-sum test, padj < 0.05) and GO enrichment in cycling glial cells (Hmgn3 WT vs EV), showing loss of pathway activation in S78A. **I**–** K** DEGs (Wilcoxon rank-sum test, padj < 0.05) and GO enrichment in UL CPNs (Nfib WT vs EV), revealing that S265 phosphorylation enhances activation of axonogenesis and guidance genes. Results link phosphorylation state to specific fate outcomes and transcriptional programs. Created in BioRender. Lusby, R. (2026) https://BioRender.com/bd0pfdn.

To functionally link the phosphorylation-dependent, stage-specific activities of Hmgn3 and Nfib to their underlying gene regulatory programs and associated cell-fate transitions, we performed in utero electroporation (IUE) of empty vector (EV) control, wild-type (WT; Hmgn3 or Nfib), and phospho-deficient mutants (Hmgn3‑S78A, Nfib‑S265A) into E13.5 mouse cortices, followed by FACS-based isolation and single-cell RNA sequencing (scRNA‑seq) at E17.5 (Fig. 7A). The scRNA-seq data resolved developmental trajectories encompassing major cortical lineages, annotated using canonical markers (Fig. 7B, C).

Pairwise comparisons between EV, WT, and mutant constructs revealed clear phosphorylation-dependent cell-fate outcomes. WT Hmgn3 overexpression led to increased proportions of intermediate progenitor cells (IPCs) ( + 11.6%), upper-layer callosal projection neurons (UL CPNs) ( + 32.8%), and cycling glial cells ( + 7.0%), alongside modest astrocyte expansion, consistent with reported roles of HMGN proteins in astrocytic differentiation^25^. These gains were accompanied by reductions in immature interneurons (INs), migrating neurons, and immature neurons. Comparison of WT vs. S78A further showed that phosphorylation enhanced progenitor-associated lineages (IPCs and UL CPNs) while the mutant favored increased IN and caudal ganglionic eminence (CGE)-derived populations (Fig. 7D and Supplementary Fig. 19A).

Similarly, WT Nfib overexpression promoted expansion of UL CPNs, CGE-derived neurons, astrocytes, and progenitor INs, while the EV control retained higher proportions of immature neuronal populations (Fig. 7E). Relative to WT, the S265A mutant displayed reduced IN and MGE-related outputs, but increased proportions of UL CPNs, astrocytes, and oligodendrocytes, suggesting that phosphorylation fine-tunes the progenitor-to-neuron transition and subsequent maturation trajectory (Fig. 7E and Supplementary Fig. 19B).

To link these lineage alterations to transcriptional mechanisms, we analyzed differentially expressed genes (DEGs) and corresponding CUT&RUN targets. In IPC populations, Hmgn3 WT vs. EV revealed 592 upregulated and 480 downregulated genes (Fig. 7F), enriched cell-cycle and nucleosome organization pathways (Fig. 7G). These gene sets overlapped with loci bound more strongly by WT than by S78A in CUT&RUN, suggesting that phosphorylation enhances Hmgn3 occupancy and activation of progenitor maintenance programs (Figs. 5H, 7H).

For Nfib, WT versus EV comparison identified 564 upregulated and 357 downregulated genes (Fig. 7I), with significant enrichment of axonogenesis and axon guidance pathways (Fig. 7J). Phosphorylated Nfib directly occupied many of these target loci, whereas S265A showed reduced binding and correspondingly diminished expression of the same genes (Fig. 7K), demonstrating that Nfib phosphorylation is required for activating axon formation programs in post-migratory neurons. Complementary pseudotime analysis of the IUE scRNA-seq data further supported these stage-specific roles: Hmgn3 activity was associated with early neural stem cell proliferation and cell-cycle progression, whereas Nfib influenced both excitatory and inhibitory neuronal outputs (Supplementary Fig. 19C–F; Supplementary Data 11).

Collectively, these findings establish a phosphorylation-dependent mechanism by which Hmgn3 drives progenitor expansion, and Nfib promotes neuronal network formation. This dual regulation underscores how temporal phosphorylation of transcription factors regulates the gene-expression programs governing cell-fate decisions and lineage maturation during cortical development.

### Site-specific phosphorylation of Hmgn3 and Nfib controls temporal progenitor competence and cell-fate specification in the developing cortex

To determine how phosphorylation of Hmgn3 and Nfib controls cell-fate specification in vivo, we combined BrdU pulse-labeling with quantitative immunohistochemistry of lineage markers in IUE brain sections, enabling direct comparison of progenitor output and fate allocation between wild-type and phospho- mutant at defined developmental stages.

**Fig. 8:**
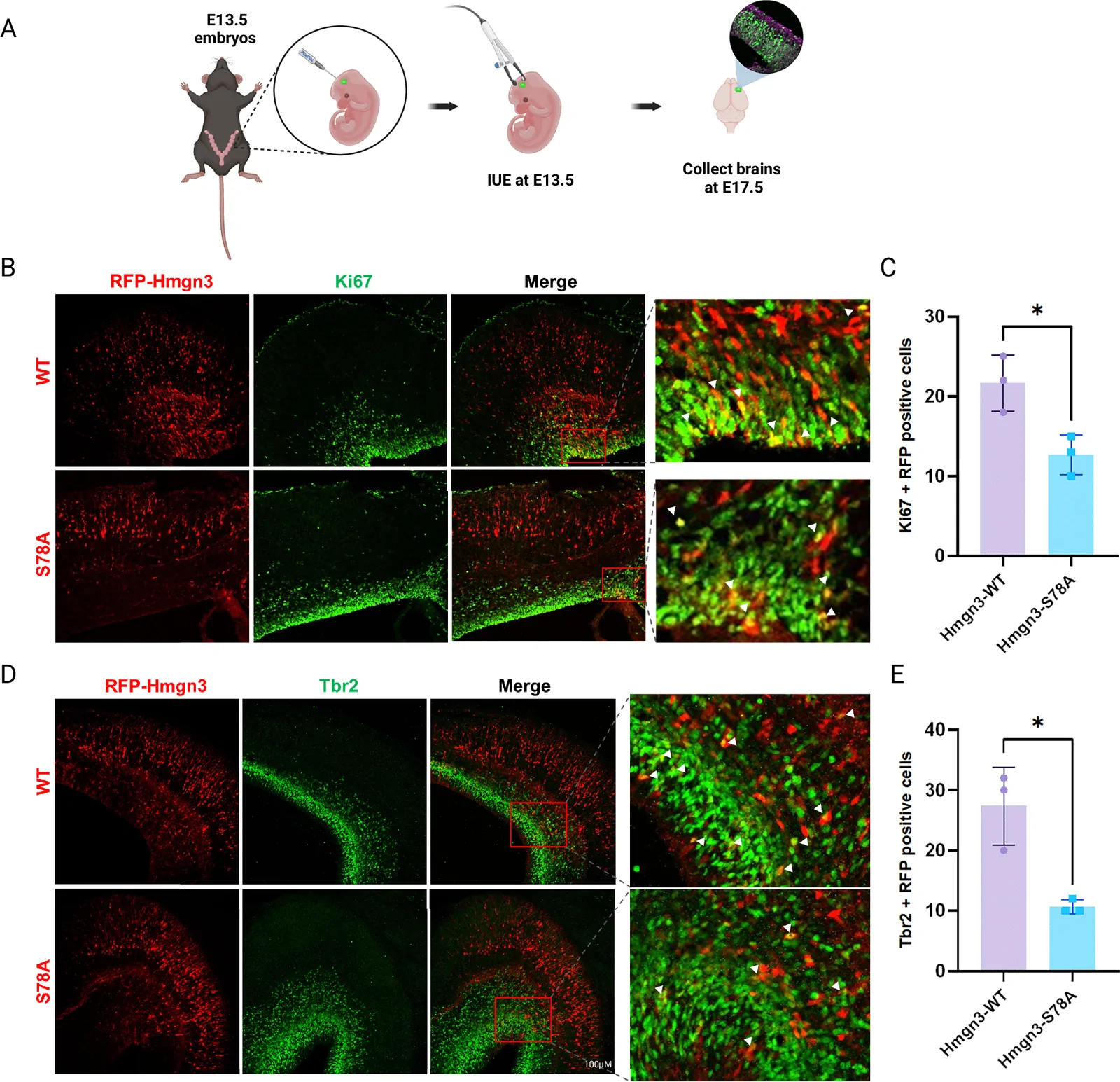
Hmgn3 S78 Phosphorylation sustains cortical progenitor proliferation and intermediate progenitor pool expansion. **A** Experimental layout for the in-utero electroporation (IUE) assay where IUE was carried out at E13.5 and analyzed at E17.5. **B**,** C** Immunofluorescence images showing changes in proliferative cell populations stained with Ki67 following overexpression of wild-type and phospho-mutant of Hmgn3, with their quantification (RFP + Ki67 + cells) represented in the bar graph (*P* = 0.026). **D**,** E** Immunofluorescence images showing changes in intermediate progenitor cell populations stained with Tbr2 following overexpression of wild-type and phospho-mutant of Hmgn3, with their quantification (RFP + Tbr2 + cells) represented in the bar graph. (*P* = 0.042). Scale bars: 100 µm. Statistical significance was calculated using a Welch’s two-tailed *t* test. *P < 0.05, **P < 0.01, ns: not significant; *n* = 3 independent biological replicates. Data are mean ± s.d. Created in BioRender. Lusby, R. (2026) https://BioRender.com/um2tqyf.

Hmgn3-WT overexpression significantly increased the proportions of proliferative cells (Ki67⁺ RFP⁺; 1.7-fold) and intermediate progenitors (Tbr2⁺ RFP⁺; 2.6-fold) relative to the S78 A phospho-mutant (Fig. 8A–E and Supplementary Data 12), findings consistent with GRN activity and scRNA-seq analyses (Figs. 2G, H, 7D). BrdU⁺ RFP⁺ cells at E17.5 were enriched within the ventricular zone (VZ) and subventricular zone (SVZ) in Hmgn3-WT cortices relative to S78A (1.7-fold increase; Supplementary Fig. 21A–C and Supplementary Data 12), confirming phosphorylation-dependent progenitor expansion. This effect was reproducible at an earlier developmental stage, with a 1.4-fold increase in BrdU incorporation observed at E15.5 following E12.5 IUE (Supplementary Fig. 21D–F). Consistent with the expanded progenitor pool, Hmgn3-WT also increased upper-layer neuron production (Satb2⁺ RFP⁺; 1.45-fold versus S78A; Supplementary Fig. 20A, B and Supplementary Data 12). Strikingly, the S78A mutant shifted output toward interneuron fates, with Gad1⁺ RFP⁺ cells increased 1.37-fold relative to WT (Supplementary Fig. 20C, D and Supplementary Data 12), demonstrating that S78 phosphorylation is required to bias progenitor output toward excitatory lineages during cortical neurogenesis.

**Fig. 9:**
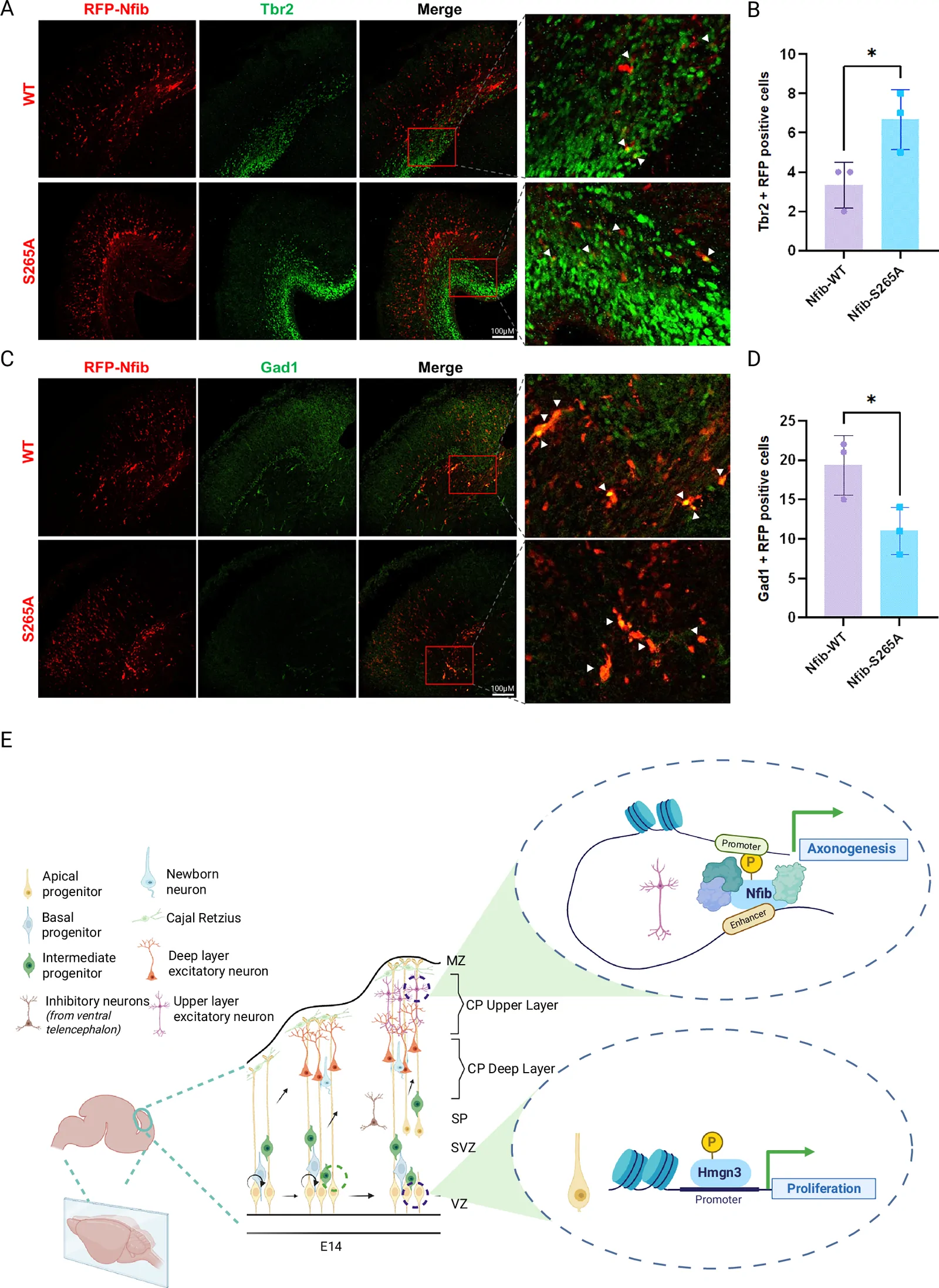
Nfib S265 Phosphorylation promotes interneuron fate specification and coordinates temporal progression of cortical lineages. **A**,** B** Immunofluorescence images showing changes in intermediate progenitor cell populations stained with Tbr2 following overexpression of wild-type and phospho-mutant of Nfib, with their quantification (RFP + Tbr2 + cells) represented in the bar graph (*P* = 0.0432). **C**,** D** Immunofluorescence images showing changes in interneuron cell populations stained with Gad1 following overexpression of wild-type and phospho-mutant of Nfib, with their quantification (RFP + Gad1 + cells) represented in the bar graph (*P* = 0.0431). **E** A schematic model illustrating the phosphorylation-dependent functional interplay between Hmgn3 and Nfib. Phosphorylated Hmgn3 promotes progenitor expansion and transition competence, while subsequent Nfib phosphorylation enhances distal enhancer targeting to activate neuronal maturation and axonogenesis. Together, their sequential, phosphorylation-dependent activities establish the temporal order of cortical cell-fate transitions. Scale bars: 100 µm. Statistical significance was calculated using Welch’s two-tailed *t* test. *P < 0.05, **P < 0.01, ns: not significant; *n* = 3 independent biological replicates. Data are mean±s.d. Created in BioRender. Lusby, R. (2026) https://BioRender.com/4g7gscm.

In contrast, overexpression of the Nfib-S265A phospho-mutant increased intermediate progenitor proportions (Tbr2⁺ RFP⁺) twofold relative to Nfib-WT (Fig. 9A, B and Supplementary Data 12). BrdU pulse-labeling at E16.5 showed that BrdU⁺ RFP⁺ cells at E17.5 were enriched in VZ/SVZ compartments under S265A conditions (1.56-fold increase versus WT; Supplementary Fig. 23E–G and Supplementary Data 12), indicating that loss of Nfib phosphorylation causes progenitor re-entry or delayed exit. This progenitor expansion was accompanied by increased production of upper-layer neurons (Satb2⁺ RFP⁺: 1.64-fold; NeuN⁺ RFP⁺: 2.5-fold versus Nfib-WT; Supplementary Fig. 23A–D and Supplementary Data 12). Complementing these excitatory shifts, Nfib-WT generated markedly more interneurons than S265A, with Gad1⁺ RFP⁺ cells increased 1.8-fold in the WT condition (Fig. 9C, D and Supplementary Data 12). These results confirm that S265 phosphorylation is specifically required for Nfib-directed interneuron specification, establishing a post-translational mechanism that distinguishes its inhibitory versus excitatory lineage outputs.

Together, the convergence of lineage tracing, BrdU birthdating, and pseudotime analyses supports a model in which site-specific phosphorylation acts upstream of lineage commitment by controlling the timing and directionality of progenitor competence transitions. The opposing fate biases produced by Hmgn3-S78A (interneuron gain) and Nfib-S265A (progenitor re-entry and excitatory expansion) at the same developmental stage argue against simple effects on proliferation rate or terminal fate bias. Instead, they reveal a phosphorylation-encoded regulatory logic that coordinates the temporal balance between excitatory and inhibitory cortical lineages during neurogenesis (Supplementary Fig. 19C–F).

## Discussion

The development of the cerebral cortex depends on the precise, sequential execution of cellular programs that guide progenitor proliferation, neuronal differentiation, and gliogenesis. A central question in developmental neurobiology is how the activity of key transcription factors (TFs) is dynamically controlled to ensure these transitions occur with proper temporal patterning. Here, we define a phosphorylation-dependent modulation of transcription factor activity that contributes to stage-specific cell-fate decisions during cortical development. This model posits that site-specific phosphorylation of two TFs, Hmgn3 and Nfib, acts as a functional rheostat that regulates both activity and genomic targeting. Through this mechanism, phosphorylation provides a layer of temporal control that ensures a smooth transition from neurogenesis to gliogenesis.

Our global proteomic and phosphoproteomic profiling revealed a profound remodeling of the post‑translational landscape during the neurogenic‑to‑gliogenic transition, marked by a greater than five‑fold increase in phosphorylated TFs. This coordinated wave of phospho‑regulation highlights phosphorylation as a primary and widespread mechanism for developmental state transitions rather than an isolated event^26–30^. While our two-timepoint design identifies functionally relevant phosphorylation differences between developmental stages, it does not resolve continuous temporal dynamics. Future studies incorporating additional developmental stages will be required to distinguish gradual, stepwise, or cyclical regulation.

Within this landscape, Hmgn3 and Nfib emerged as key phosphor-regulated TFs that act sequentially (Fig. 9E). Hmgn3 functions as a progenitor maintenance factor that sustains proliferation and chromatin accessibility in early progenitors^31^. Phosphorylation at S78 tempers its proliferative activity, acting as a molecular brake that prevents excessive expansion of the progenitor pool. Loss of this modification (S78A) leads to hyper-expansion and cortical malformations, indicating that phosphorylation-dependent attenuation of Hmgn3 is critical for progenitor to neuron transition. As Hmgn3 activity subsides, Nfib assumes the role of a maturation driver, governing neuronal migration, axonogenesis, and glial maturation^32–35^. The severe migration failure of the Nfib S265A mutant underscores that phosphorylation at this site is indispensable for its function in vivo.

Mechanistically, our CUT&RUN and molecular dynamics analyses reveal that phosphorylation provides a genomic targeting signal. S265 phosphorylation on Nfib directs it to distal enhancers controlling forebrain-specific gene programs, whereas the unphosphorylated mutant is misdirected to promoter-proximal, cell-cycle regions, explaining its pronounced loss-of-function phenotype. This “phosphorylation‑as‑a‑GPS” model illuminates how transient signaling cues can be stably encoded into gene-regulatory outcomes through altered TF–DNA interactions.

Our single-cell transcriptomic analyses further demonstrate that phosphorylation state dictates lineage allocation (Fig. 9E). For Hmgn3, phosphorylation promotes progenitor proliferation and nuclear processes early in development^36,37^, later facilitating gliogenesis^25^. Conversely, phosphorylated Nfib favors neurogenic outcomes, particularly interneuron differentiation, whereas loss of phosphorylation shifts neural cell fate toward astroglial lineages. Previous studies have demonstrated that Nfib plays an important role in interneuron generation and maturation during cortical development^35^. Our findings not only confirm the role of Nfib in interneuron specification but further demonstrate that S265 phosphorylation is required for this developmental fate decision during cortical development. The developmental gradient in Nfib phosphorylation from hyperphosphorylation at E14.5 to hypophosphorylation at E18.5 mirrors the transition from neuronal migration to axonal maturation, linking phosphorylation to the temporal patterning of lineage identity^32–35^.

Earlier-stage electroporation (E12.5) revealed enhanced sensitivity of early progenitor populations to Hmgn3 phosphorylation, with more pronounced effects on progenitor expansion and fate specification compared to E13.5 manipulation. This stage-dependent sensitivity suggests that phosphorylation-dependent regulation is particularly critical during early progenitor expansion phases, complementing our findings from E13.5 perturbations that capture effects during the neurogenic-to-gliogenic transition.

Together, our observations establish phosphorylation as a pivotal regulatory axis that controls TF activity, temporal patterning, and cell-fate specification. By modulating the balance between progenitor, neuronal, and glial populations, phospho‑dependent control ensures the orderly assembly of cortical circuits. Disruption of these molecular regulatory mechanisms could contribute to neurodevelopmental disorders characterized by altered cell‑type proportions.

In summary, our work provides a systems‑to‑mechanism framework for understanding how temporal phosphorylation of transcription factors coordinates developmental state transitions. By delineating a sequential phospho‑switch between Hmgn3 and Nfib that balances progenitor maintenance with neuronal differentiation, we uncover a unifying principle of developmental temporal patterning and demonstrate how post‑translational signals integrate with gene regulatory networks to construct the cerebral cortex.

## Methods

### Proteome and phosphoproteome profiling of mouse cortical development

Cortex was dissected from C57BL/6 J mouse E14.5 and E18.5 embryos (*n* = 3), lysed with gentle lysis buffer [10 mM Tris (pH 7.5), 150 mM NaCl, 1 mM EDTA, 0.5% NP-40 and protease and phosphatase inhibitor cocktail (Roche)]. Sample preparation and the LC-MS/MS run were performed as previously described^38^. Briefly, for the whole proteome and phosphoproteome (*n* = 3 per stage) data during neurogenesis (E14.5) and gliogenesis (E18.5) stages, samples were digested and labeled with 6-plex tandem mass tag (TMT) and run on ultra-high performance liquid chromatography-MS/MS (UHPLC-MS/MS) on a Thermo Fisher platform. Peptide spectra were mapped against the mouse proteome, downloaded from UniProt in February 2023, using MaxQuant version 2.2.0. Phosphorylation sites at both stages were identified under default parameters using MaxQuant^39,40^. To quantify phosphorylation, we normalized the phosphorylation intensity of each site using the corresponding protein’s total intensity. Only phosphorylation events with a localization probability of ≥ 0.8 were considered for further analysis. Differential phosphorylation analysis between E14.5 and E18.5 stages was conducted using the Student’s *t* test (*P* ≤ 0.05). Functional enrichment analysis of differentially phosphorylated proteins was performed using EnrichR^41^. To investigate the novelty of the phosphorylation sites and identify potential kinases responsible for phosphorylation, phosphorylated sequences were submitted to the PhosphoSitePlus webserver^42,43^. We also assessed the impact of amino acid substitutions at phosphorylation sites on protein stability and pathogenicity using DUET, PMut, and AlphaFold predictions^44–46^. To assess the presence of neurodevelopmental disorder (NDD) variants in the proximity of candidate genes Hmgn3 and Nfib phosphorylated sites, we utilized two specialized databases. The SysNDD^47^ database was employed to identify variants associated with NDDs, while the SAFARI ASD (https://gene.sfari.org/) Risk Gene Database was used to evaluate variants linked to autism spectrum disorder (ASD) risk.

### Single-cell RNA-seq data acquisition and processing

We obtained the raw count matrix for E14.5 and E18.5 stages from previously published single-cell RNA sequencing (scRNA-seq) datasets of the mouse prefrontal cortex^48^. The raw count data were processed using Seurat^49,50^, where normalization, clustering, and dimensional reduction were performed using default parameters. Cells present at each developmental stage were clustered and annotated accordingly.

### Construction of gene regulatory networks

To construct gene regulatory networks (GRNs), we utilized the gene expression matrix across different cell types. PySCENIC^51,52^ was used with default parameters to identify activated GRNs for each cell type during the neurogenesis and gliogenesis stages. We identified the top transcription factors (TFs) driving cell specificity at both developmental stages by analyzing their regulon specificity score (RSS) and their target gene activity. These TFs were further analyzed to check their phosphorylation status, enabling the identification of differentially phosphorylated and activated TFs between neurogenesis and gliogenesis stages.

### Transcription factor candidate prioritization

Candidate transcription factors were selected from the phosphoproteomic dataset based on: (i) significant stage-associated phosphorylation, (ii) integration with gene regulatory network activity, (iii) complementary developmental expression patterns, and (iv) feasibility for experimental validation.

### Plasmids

Hmgn3 and Nfib genes were amplified from the cDNA derived from the E14.5 mouse cortex and cloned into the pCAG-mScartet plasmid. Phospho mutants Hmgn3-S78A and Nfib-S265A were introduced by the site-directed mutagenesis method as described previously^53^. Plasmid Maxi prep was done using NucleoBond Xtra Maxi Plus endotoxin-free plasmid DNA kit (cat no: 740426.50) as per the manufacturer's instructions.

### *In utero* electroporation (IUE)

Plugged C57BL/6 J females were either purchased from Janvier Labs or obtained from the core breeding unit at BML, University of Southern Denmark, and E0.5 was defined as the timepoint on which the vaginal plug appeared. Mice either at E12.5 or E13.5 (n = 3 embryos per condition per timepoint) were subjected to *in utero* electroporation and embryos harvested as previously described^54^. Electroporation was performed using the pCAG-mScarlet-3XFlag vector encoding for Nfib-WT, Nfib-S265A, Hmgn3-WT, Hmgn3-S78A, and an empty vector served as a control. In brief, plasmids were prepped using an NncleoBond Xtra Maxi Endotoxin-free Plasmid maxi kit (cat no: 740426.50) and plasmid concentration adjusted to 1.5 μg/μl. Pregnant mice were anesthetized with isofluorane at E13.5, and ∼ 1.5 μl of plasmid solution was injected into the ventricle of embryonic brains. The plasmids were electroporated into the dorsal ventricular zone with six pulses of 30 V, 50 ms each at 1 s interval delivered through platinum electrodes using round electrodes CUY650P5 and Nepa Gene electroporator (NEPA21 Type II). After three to four days of electroporation (E15.5 or E17.5), the brain tissues were processed for the analyses.

### BrdU incorporation experiments

To assess lineage progression and temporal patterning, BrdU pulse-labeling was performed following in utero electroporation. BrdU (0.15 mg/g body weight) was administered at defined developmental stages (E14.5 and E16.5), and embryos were collected at later timepoints (E15.5 and E17.5) for analysis. BrdU⁺ RFP⁺ cells were quantified across cortical layers and cell-type markers. For each condition: > 500 labeled cells were analyzed, across (*n* = 3) embryos.

### Immunohistochemistry and quantification

Brains were fixed in 4% paraformaldehyde in 0.1 M phosphate buffer (PFA, pH 7.4) overnight at 4 °C, cryoprotected in 30% sucrose and cryosectioned (15 µm). Immunohistochemistry was performed as previously described^54^ using anti-Tbr2 (1:200, Abcam, ab23345), anti-Ki67 (1:500, Thermo, 14-5698-82), anti-Satb2 (1:100, Abcam, ab92446), anti-NeuN (1:400, SYSY, 266 004), anti-Gad1 (1:200, SYSY, 198 308), anti-BrdU (1:400, Proteintech, 66241-1-Ig), anti-Nfib (1:100, Sigma, HPA003956), and anti-Hmgn3 (1:100, Abcam, ab72233) with DAPI used to counterstain nuclei.

For BrdU immunohistochemistry, sections were washed three times with PBS and incubated in 2 N HCl for 30 min at 37 °C for DNA denaturation prior to blocking and primary Brdu antibody staining.

Images were acquired using a Nikon Ti2 confocal microscope. Electroporated cells were identified by fluorescent reporter expression (RFP). For each condition: ≥300 cells were quantified per marker, across *n* = 3 embryos. Pictures were digitally assembled using NiS elements software 6.0 (Nikon) and composites analyzed using ImageJ. Targeted cells after *in utero* electroporation were identified based on RFP expression, and the relevant region of the lateral cortex was selected as a cortical column with boundaries perpendicular to the apical border. Cortical layers of the VZ, SVZ, IZ and CP were identified upon Tbr2 immunoreactivity, which defines the SVZ, and by the characteristic low density of DAPI^+^ nuclei in the IZ. The number of cells positive for any given marker was finally calculated as a proportion of the originally targeted cell pool (RFP^+^). Images were quantified using ImageJ software, and GraphPad Prism was used for the statistics and images.

### Isolation of electroporated cells

For the isolation of electroporated cells from E17.5, the dorsolateral neocortex (*n* = 3-4) was dissected, pooled, and a single-cell suspension was produced per condition using the MACS Neural Tissue Dissociation kit containing papain (Miltenyi Biotec, 130-092-628).

The cells were resuspended to 1 million cells/ml in FACS buffer (3%BSA, 1 mM EDTA in HBSS w/o Ca2 + and Mg2 + ), passed through a 40 μm filter and immediately processed by FACS on a five-laser Invitrogen BIGFOOT cell sorter. For each condition, 30,000 to 50,000 cells were collected in 1.5 mL protein LoBind tubes containing FACS buffer.

### scRNA-sequencing cell fixation preparation

scRNA-seq libraries were generated according to Parse Biosciences protocols: Evercode Cell Fixation v3 and Evercode WT Mini v3. For cell fixation, FACS sorted single cell suspensions were centrifuged for 10 min at 200 × *g* at 4 °C in 15 ml BSA-coated Falcon tubes and resuspended in Cell Prefixation Buffer with BSA 7.5% (Gibco) in 15 ml BSA-coated Falcon tubes. Cell suspensions were incubated in fixation solution, followed by the addition of permeabilization solution and stop buffer, according to the manufacturer’s instructions. Cell suspensions were frozen using a Mr. Frosty Freezing container (Sigma-Aldrich) at − 80 °C until further use. Samples barcoding and sub-libraries were prepared using the Evercode WT Mini v3 as per the manufacturer’s instructions and sequenced on a NovaSeq 6000 instrument.

### WT and Phospho mutant single-cell RNA-seq data analysis

Single-cell transcriptomic libraries were prepared using the Evercode WT Mini kit (Parse Biosciences, Chemistry v3) according to the manufacturer’s protocol. Sequencing was performed on two sublibraries, and raw FASTQ files were processed using the Trailmakers pipeline (Parse Biosciences), which includes demultiplexing, read alignment, and generation of gene-cell count matrices. Reads were mapped to the GRCm38 (mm10) mouse reference genome.

Downstream analysis was performed in R using the Seurat package (v4.0)^49^ Quality control, normalization, dimensionality reduction, clustering, and visualization were conducted following standard Seurat workflows. Cell type annotation was performed based on the expression of established marker genes for the major cortical population^48^. Developmental trajectory inference was performed using CytoTRACE v2^55^, which estimates differentiation states and reconstructs lineage trajectories.

### CUT&RUN

CUT&RUN experiments were performed on E17.5 IUE sorted cells using specific antibodies for the following proteins: anti-Flag (Sigma, M2 clone F1804) and normal IgG. Biological replicates (*n* = 2 per condition) were generated for all the CUT&RUN assays. Samples were prepared following the EpiCypher CUTANA ChIP/CUT&RUN Kit protocol (Epicypher, SKU: 14-1048). An IgG antibody served as a negative control to evaluate non-specific background signal from chromatin captured during immunoprecipitation, and an E. Coli spike-in control was added for normalizing enrichment during analysis. CUT&RUN libraries were prepared according to the CUTANA™ CUT&RUN Library Prep Kit (Epicypher) for Illumina, and each sample was uniquely barcoded with the Multiplex Oligos for Illumina. Libraries were pooled and sequenced using 75-base pair paired-end reads on the NovaSeq 6000 platform, obtaining a sequencing depth of at least 10, 12 million reads per sample.

### CUT&RUN and ATAC-seq data processing and analysis

CUT&RUN was performed on IUE FACS-sorted cells to profile the genome-wide binding of Nfib wild-type (WT) and S265A mutant, Hmgn3-WT and S78A mutant. The experiment included two biological replicates for each condition, and IgG served as a control. The sequencing reads were subjected to quality control using FastQC (v0.12.1) and adapter trimming with Cutadapt^56^ (v4.6). Reads were aligned to the mouse reference genome mm10 using Bowtie2^57^ (v2.4.4), followed by duplicate removal and quality filtering using Picard (v3.1.0) and Samtools^58^ (v1.15.1). Peaks were called using MACS2^59^, and ChIPseeker^60^ was used for downstream genomic analysis and annotation. Signal tracks were generated using DeepTools^61^ (v3.5.1) for visualization.

The ATAC-Seq data covers E11.5 to P0, derived from the embryonic mouse forebrain, were obtained from previous public data^62^. To process the ATAC-Seq, the default ENCODE ATAC-seq pipeline (http://github.com/ENCODE-DCC/atac-seq-pipeline) is applied to the ATAC-Seq FASTQ file. The overlap peaks between the development course ATAC-Seq and Nfib CUT&RUN are calculated by the BEDTools^63^ intersect function with the parameter of -f 0.2.

### Whole transcriptome generation and analysis

Whole transcriptome sequencing was performed using E14.5 and E18.5 mouse cortex (*n* = 3 biological replicates per stage). Sequencing libraries were prepared and sequenced to a depth of approximately 10 million reads per sample. Adapter sequences were trimmed from the raw FASTQ files using Cutadapt^64^ (v4.9). High-quality adapter-trimmed reads were then mapped to the mouse reference genome (mm10) using the STARlong utility within the STAR aligner^65^. Following read alignment, transcriptome assembly and gene expression quantification were conducted using StringTie2^66^ with default parameters applied to each replicate at different stages. For differential gene expression analysis between stages, we utilized the DESeq2^67^ package, which provided fold-change estimates and significance testing for each gene. We performed deconvolution of the bulk transcriptome to check the cell type proportion using a reference from the corresponding single-cell data with the granulator. To gain further insights into the biological processes associated with the differentially expressed genes, we performed Gene Set Enrichment Analysis (GSEA) using the clusterProfiler^68^ package.

### In-silico perturbation analysis

We performed in-silico perturbations using CellOracle for key TFs, Hmgn3 and Nfib, to simulate the effects of their perturbation during the neurogenesis stage (E14.5). The impact on developmental flow and cell differentiation was assessed, providing insights into their role in neuronal development.

### System modeling

#### Nfib

The full-length sequence of the Nfib was obtained from UniProt^69^ (ID: P97863), which corresponds to Mus musculus. The 3D structure of the protein was modeled using AlphaFold^70^. Further structural modeling was carried out by PyMOL software, where residues P15 – P272 were removed to build up the final model.

The DNA structure belonging to *mus musculus* was retrieved from the Protein Data Bank (PDB ID: 9QKY)^71^. Four identical 31-base pair double-stranded DNA molecules were present in the reference DNA structure, and only one of them was retained in the model. The modeled Nfib protein was aligned to the selected DNA, ensuring that both molecular structures shared the same coordinates. S265 is phosphorylated using the SP2 option in the CHARMM-GUI server.

#### Hmgn3

The nucleosome core particle, containing DNA and histones, was retrieved from the PDB (PDB ID: 9D3M)^72^. This structure represents an assembled double-stranded DNA from *xenopus borealis*, eight histone chains (H3, H4, H2A and H2B), and two copies of the short-sequence Hmgn2 protein.

From the UniProt database, the full *mus musculus* amino acid sequence for HMGN3a identified (ID: Q9DCB1). Two copies of the Hmgn3 were modeled using AlphaFold. The crystal Hmgn2 contains a conserved RRSARL motif, which is shared by Hmgn3. Therefore, Hmgn3 models were aligned with respect to the reference Hmgn2 common region using PyMOL to ensure consistent spatial coordinates. S78 is phosphorylated using the SP2 option in the CHARMM-GUI server.

### System preparation

Preparation of the Coarse-Grained (CG) model of the DNA–protein complex followed the Martini standard workflow, based upon the Martini Tutorial for protein–DNA Systems (https://cgmartini.nl/docs/tutorials/Legacy/martini2/dna.html). Each preparation step was executed within GROMACS v2023^73^. All atom protein and DNA structures were initially converted to GROMACS-compatible formats via the gmx editconf module.

DNA was converted to coarse-grained representation using martinize-dna.py, where ds-stiff was set to maintain the rigidity of double-stranded DNA. The non-phosphorylated protein was coarse-grained using martinize.py, and the phosphorylated protein was coarse-grained using martini25.py^74^, utilizing the Martini force field (martini22) in both instances^75^. Elastic Network Force Constant and Upper Cutoff Distance defaults were employed. Secondary Structure Predictions from DSSP were utilized to assign backbone restraints.

A dodecahedral simulation box was created via gmx editconf, and the system was centered within the box. This was followed by the solvation process of the system, where gmx solvate module was employed, and a previously equilibrated Martini water model was utilized. The system was then ionized and neutralized by 0.15 M KCl concentration with the help of gmx genion module^73^. The system was then used as the initial configuration for subsequent molecular dynamics simulations.

### Coarse-grained molecular dynamics simulations

CG molecular dynamics simulations were performed using GROMACS 2023 following the standard Martini 2.2 protocol. Systems were energy-minimized using the steepest descent method, followed by NPT equilibration for 250 ns using a velocity-rescale thermostat and C-rescale barostat. Production simulations were run for 5 μs with a 20 fs time step under periodic boundary conditions.

Electrostatic interactions were treated using the reaction field method (εᵣ = 15; cutoff = 1.1 nm), and van der Waals interactions employed a 1.1 nm cutoff with potential-shift Verlet modifications. Three independent replicates were performed for each phosphorylated and non-phosphorylated system, each initiated with different velocity distributions.

### Analysis

Dynamic Cross Correlation Map: Correlations (both correlated and anti-correlated) in motion for each residue pair were measured with dynamic cross-correlation maps (DCCMs), which are calculated via the Bio3D package^76^. The analysis was carried out on backbone beads only. Only backbone bead residues were used in the analysis of these correlations. As described in the equation below, the correlation coefficient can be defined by:

$${DCC}\left(i,j\right)=\frac{{\left\langle \Delta {r}_{i}\left(t\right)\cdot \Delta {r}_{j}\left(t\right)\right\rangle }_{t}}{\sqrt{{\left\langle {{{\rm{||}}}\Delta {r}_{i}\left(t\right){{\rm{||}}}}^{2}\right\rangle }_{t}}\sqrt{{\left\langle {{{\rm{||}}}\Delta {r}_{j}\left(t\right){{\rm{||}}}}^{2}\right\rangle }_{t}}},$$

where ${r}_{i}\left(t\right)$ and ${r}_{j}\left(t\right)$ represent the positions of atoms *i* and *j* at time *t*, accordingly, while 〈·〉ₜ denotes the time average. The displacement vectors $\Delta {r}_{i}\left(t\right)$ and $\Delta {r}_{j}\left(t\right)$ correspond to deviations from the respective time-averaged positions. Correlation coefficients can vary from − 1 to 1, meaning that when a correlation coefficient is positive, it indicates correlated motion of the two residues; conversely, a negative value indicates that they have anti-correlated motion.

#### Number of contacts

Identification of intermolecular interactions in the simulated systems was analyzed through the calculation of the contacts number at the interfaces between proteins and nucleosomes. These calculations were completed for all trajectory files, by utilizing the gmx mindist module by GROMACS. The contact distances used for this analysis had a cutoff distance of 0.6 nm to define interatomic contact distances. Contact numbers were then determined for each frame from the 5 µs simulations and then averaged over three independent replicates for each system.

### Statistical analysis and visualization

Statistical analyses and data visualizations were performed using R and Python. Custom scripts were developed to analyze experimental data, generate figures, and perform statistical tests. Schematic illustrations of the experimental design were created using BioRender. Post-translational modifications (PTMs) and variants were mapped onto protein sequences using the PROTTER^77^ webserver. Confocal images and Western blot images were processed using ImageJ software. GraphPad Prism software was used for the statistical calculations.

### Reporting summary

Further information on research design is available in the Nature Portfolio Reporting Summary linked to this article.

## Supplementary information

Supplementary Information

Description of Additional Supplementary Files

Supplementary Data 1-12

Reporting Summary

Transparent Peer Review file

## Source data

Source Data

## Data Availability

All datasets generated in this study are publicly available. Bulk RNA sequencing data generated during this study have been deposited in the Gene Expression Omnibus (GEO) under accession number GSE302501. Proteome and phosphoproteome datasets are available via the ProteomeXchange Consortium under accession number PXD066619. CUT&RUN sequencing data have been deposited in GEO under accession number GSE327199, and single-cell RNA-seq data from in utero electroporation experiments (Parse Biosciences platform) are available under accession number GSE327212. Previously published datasets used in this study include single-cell RNA-seq and single-cell ATAC-seq datasets of mouse cortical development (E10.5–P4 and E13.5–E18.5, respectively) are available under GSE153164. All other data supporting the findings of this study are available from the corresponding authors upon request. Source data are provided in this paper.

## Code availability

No custom code was generated for this study. All analyses were performed using publicly available software as described in the Methods.
