# Supplementary material for: Phosphorylation-dependent control of transcription factor activity regulates temporal patterning of cortical cell fate specification
Source: Nat Commun. 2026 Sep 4;17:10605. doi: 10.1038/s41467-026-77195-8 (PMC13645745; doi:10.1038/s41467-026-77195-8)
Supplement: Supplementary file 1 [file 41467_2026_77195_MOESM1_ESM.pdf]

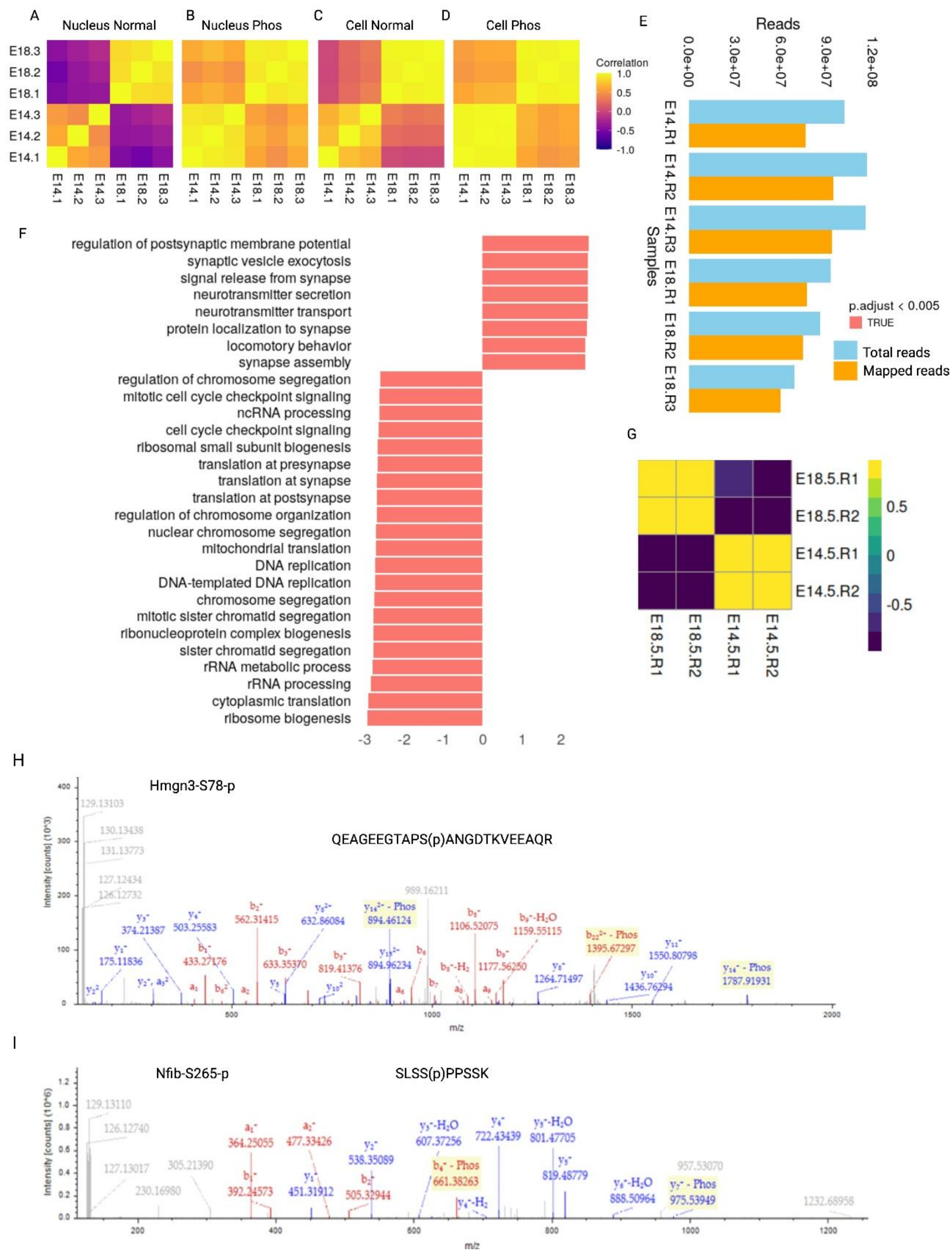

Supplementary Figure 1. Quality assessment and reproducibility of proteomic and transcriptomic datasets. (A–D) Correlation heatmaps of biological replicates (n=3) for the whole-cell and nuclear proteomes (A, B) and phosphoproteomes (C, D), demonstrating high reproducibility within each stage and clear separation of neurogenic (E14.5) and gliogenic (E18.5) cortices. (E) Bar plot showing distribution of total and mapped RNA-seq reads for each biological replicates (n=3). (F) GSEA highlighting top enriched (adjusted p value ≤ 0.05) biological processes for genes upregulated at E14.5 (neurogenic) and E18.5 (gliogenic) stages. (G) Correlation heatmap of

bulk RNA-seq replicates confirming dataset consistency. (H–I) Identified phosphorylation sites on Hmgn3 and Nfib across three biological replicates. Together, these analyses confirm high data quality and reproducibility across omic layers.

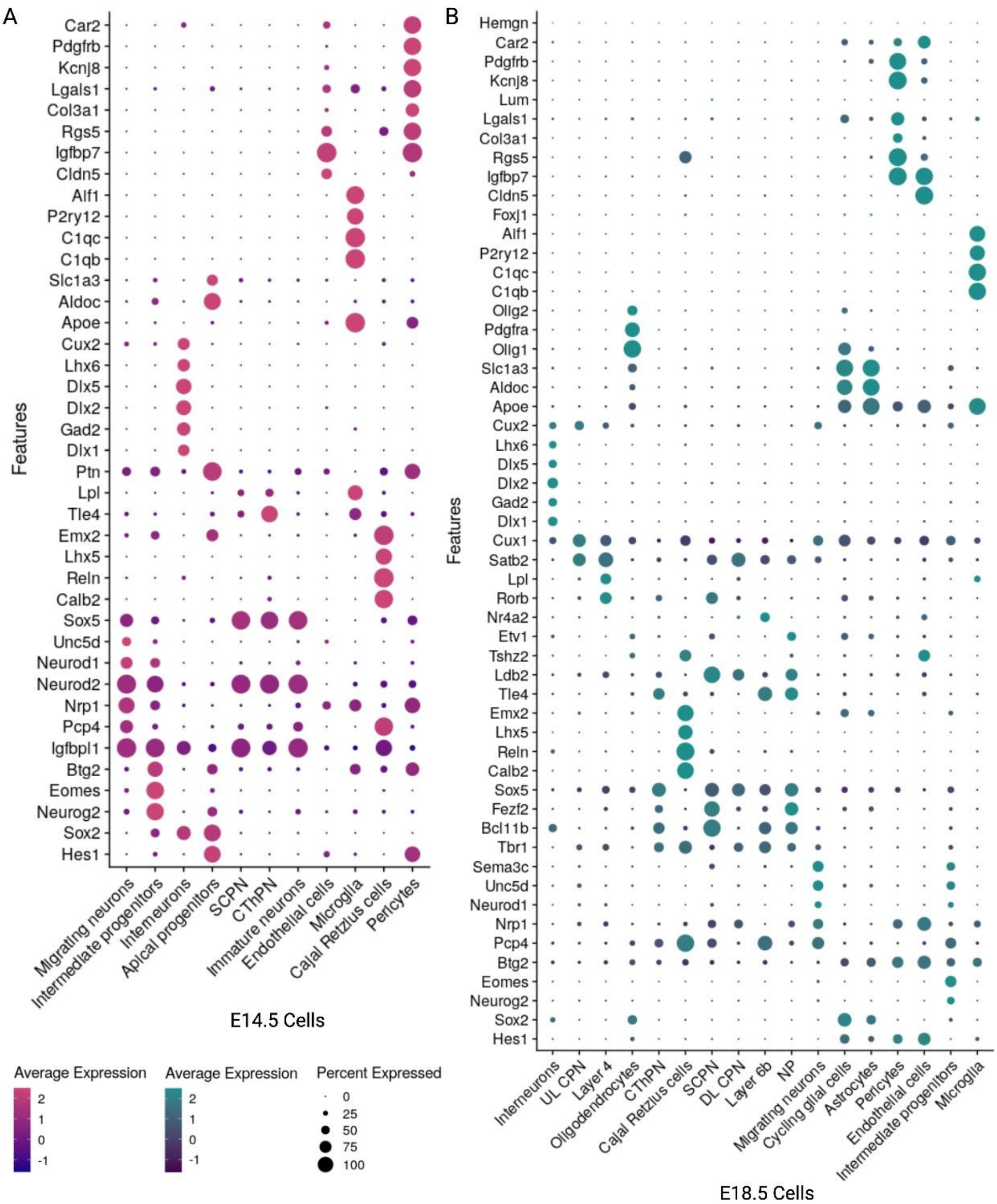

Supplementary Figure 2. Marker-based annotation of cell types at neurogenic and gliogenic stages. (A, B) UMAP visualizations showing canonical marker expression used to define cell populations at E14.5 (A) and E18.5 (B).

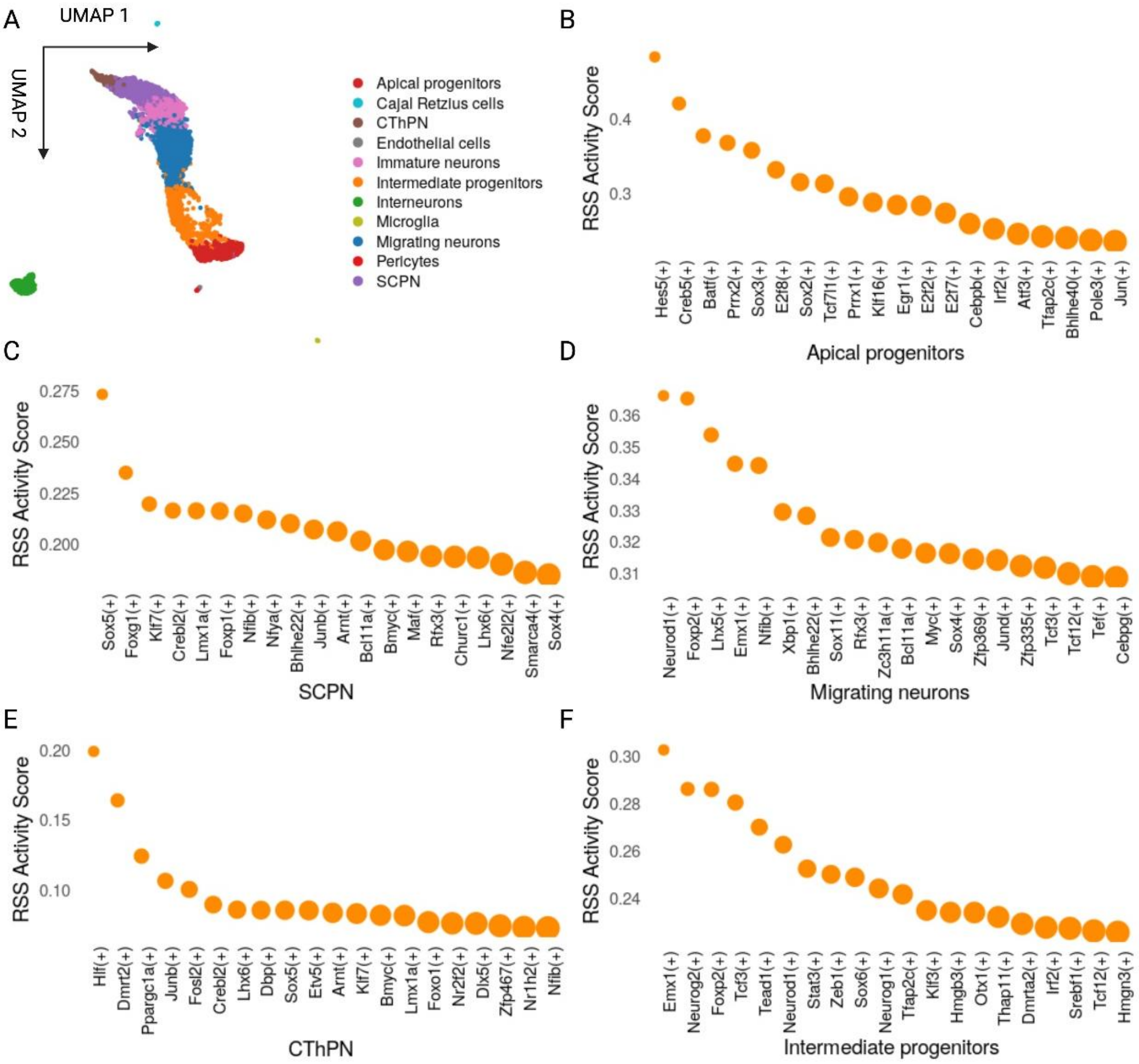

Supplementary Figure 3. Regulons defining cell-type specificity during neurogenesis (E14.5, Part 1). (A) UMAP of E14.5 cell clusters. (B–F) Regulon specificity score (RSS) analysis showing top TFs driving identity of (B) apical progenitors (Hes5), (C) subcerebral projection neurons (Foxg1), (D) migrating neuron (Neurod1), (E) corticothalamic projection neurons (Sox5), and (F) intermediate progenitors.

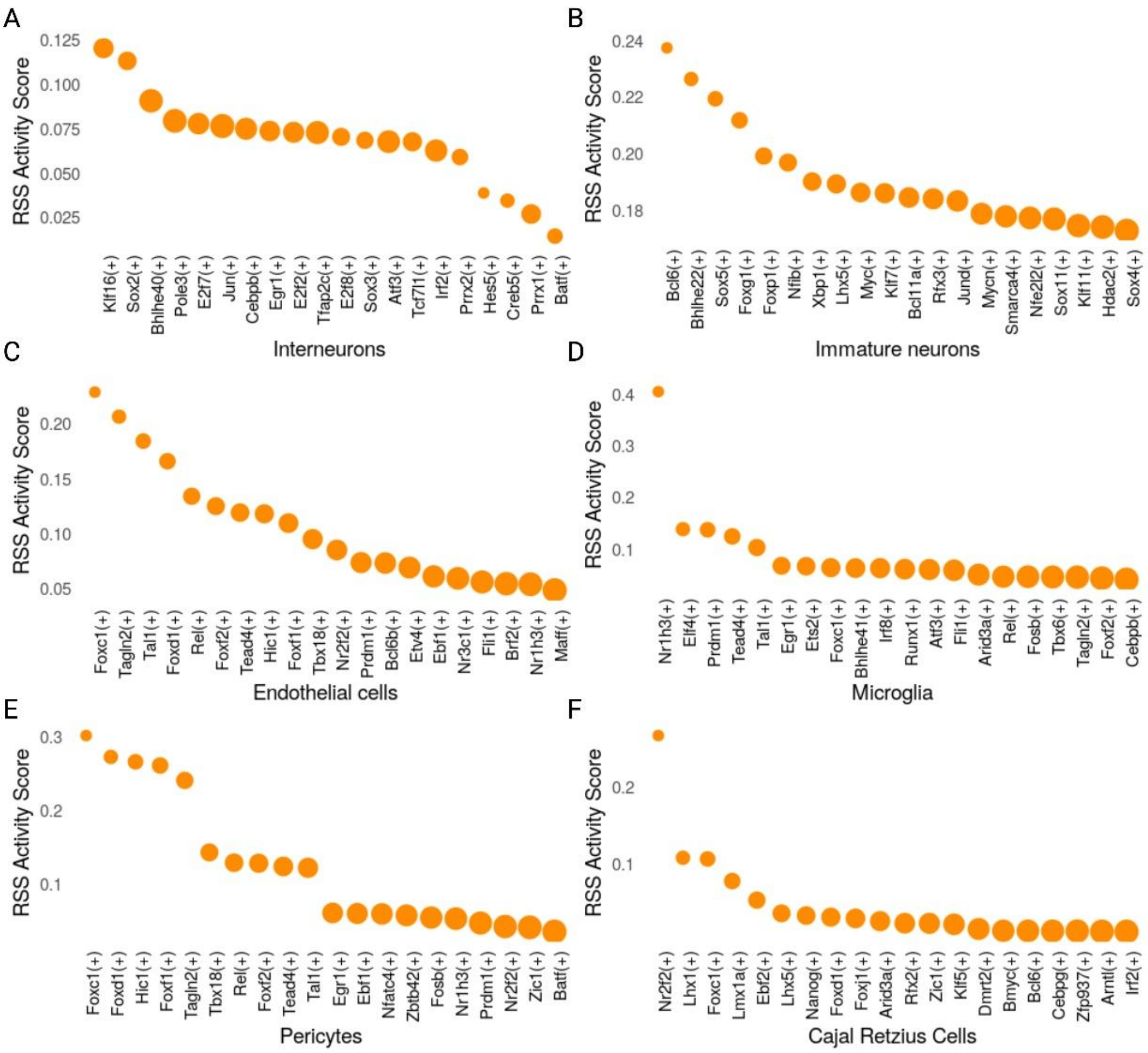

Supplementary Figure 4. Regulons defining additional E14.5 cell populations (Part 2). RSS analysis identifies dominant TFs for (A) interneurons (Sox2), (B) immature neurons (Bhlhe22), (C) endothelial cells, (D) microglia, (E) pericytes, and (F) Cajal–Retzius cells, demonstrating cell-type-specific transcriptional programs during neurogenesis.

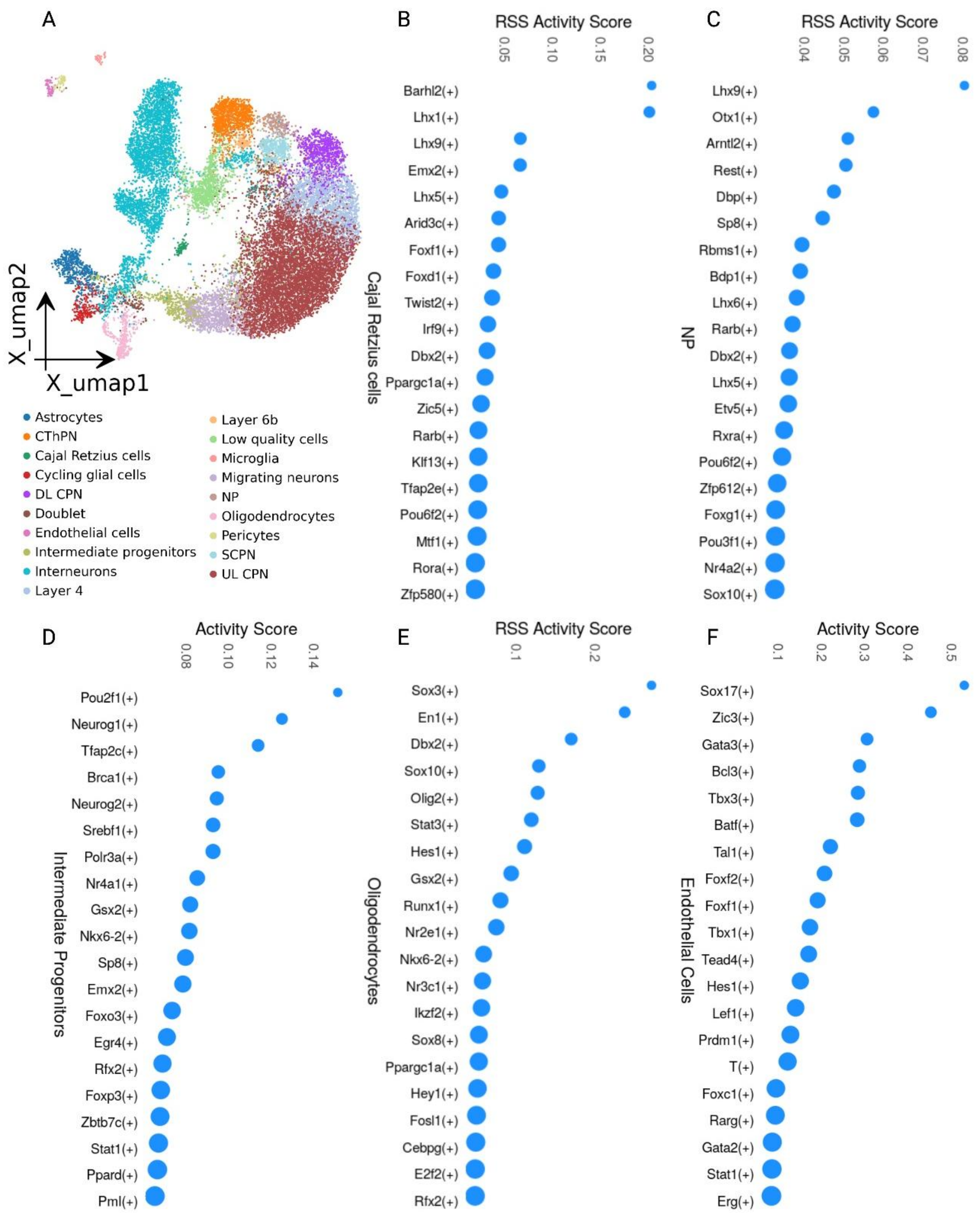

Supplementary Figure 5. Key regulons during gliogenesis (E18.5, Part 1). (A) UMAP of cell clusters at E18.5. (B–F) Major regulons identified for (B) Cajal–Retzius cells, (C) neural progenitors (Stat1), (D) intermediate progenitors, (E) oligodendrocytes (Olig2), and (F) endothelial cells, reflecting maturation of distinct lineages.

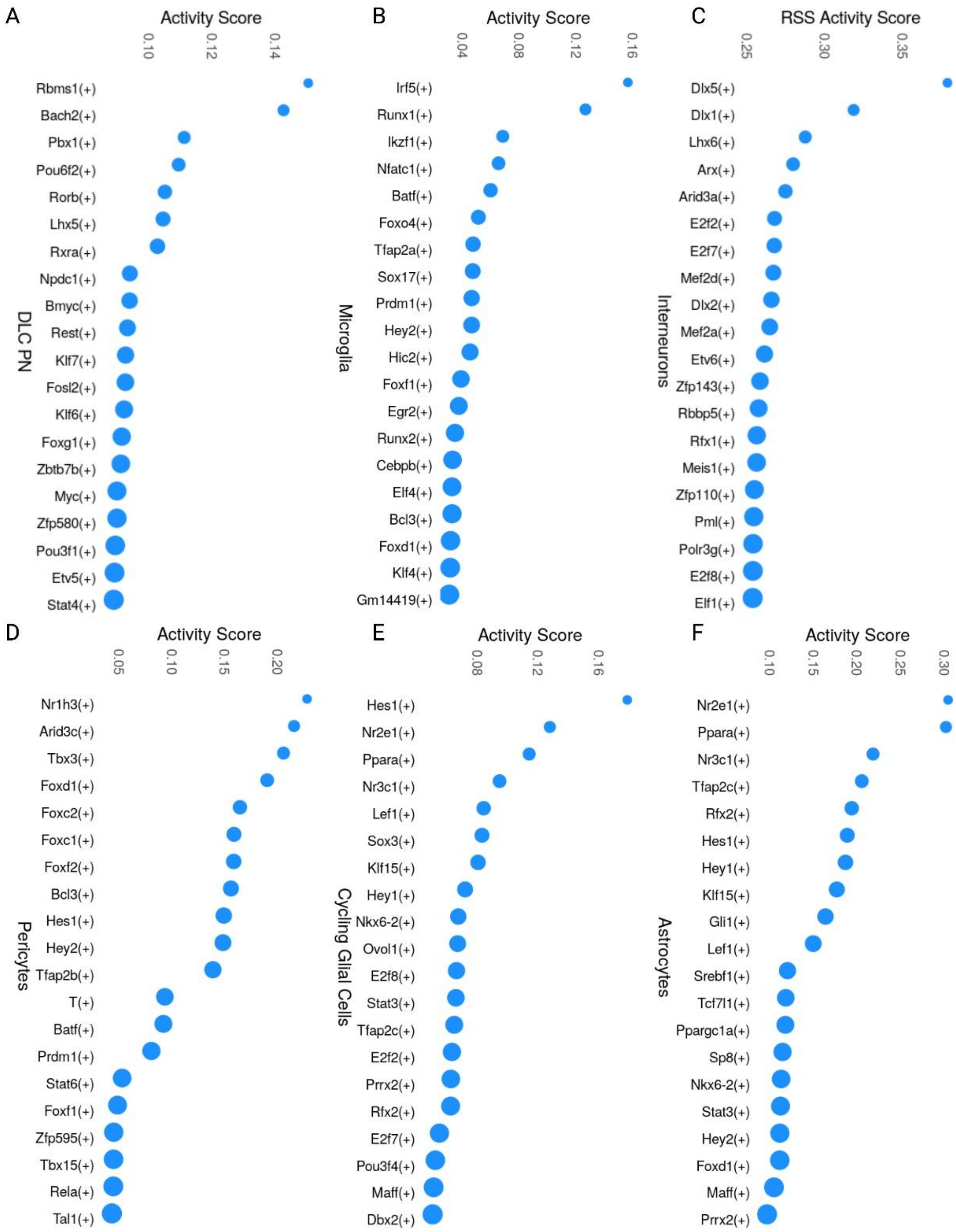

Supplementary Figure 6. Regulons defining additional gliogenic cell types (E18.5, Part 2). (A–F) Top regulons for (A) dorsolateral cortical projection neurons (DL CPNs), (B) microglia, (C) interneurons (Dlx1), (D) pericytes, (E) cycling glial cells, and (F) astrocytes. Transcriptional control patterns reveal expansion of gliogenic and immune modules at late embryonic stages.

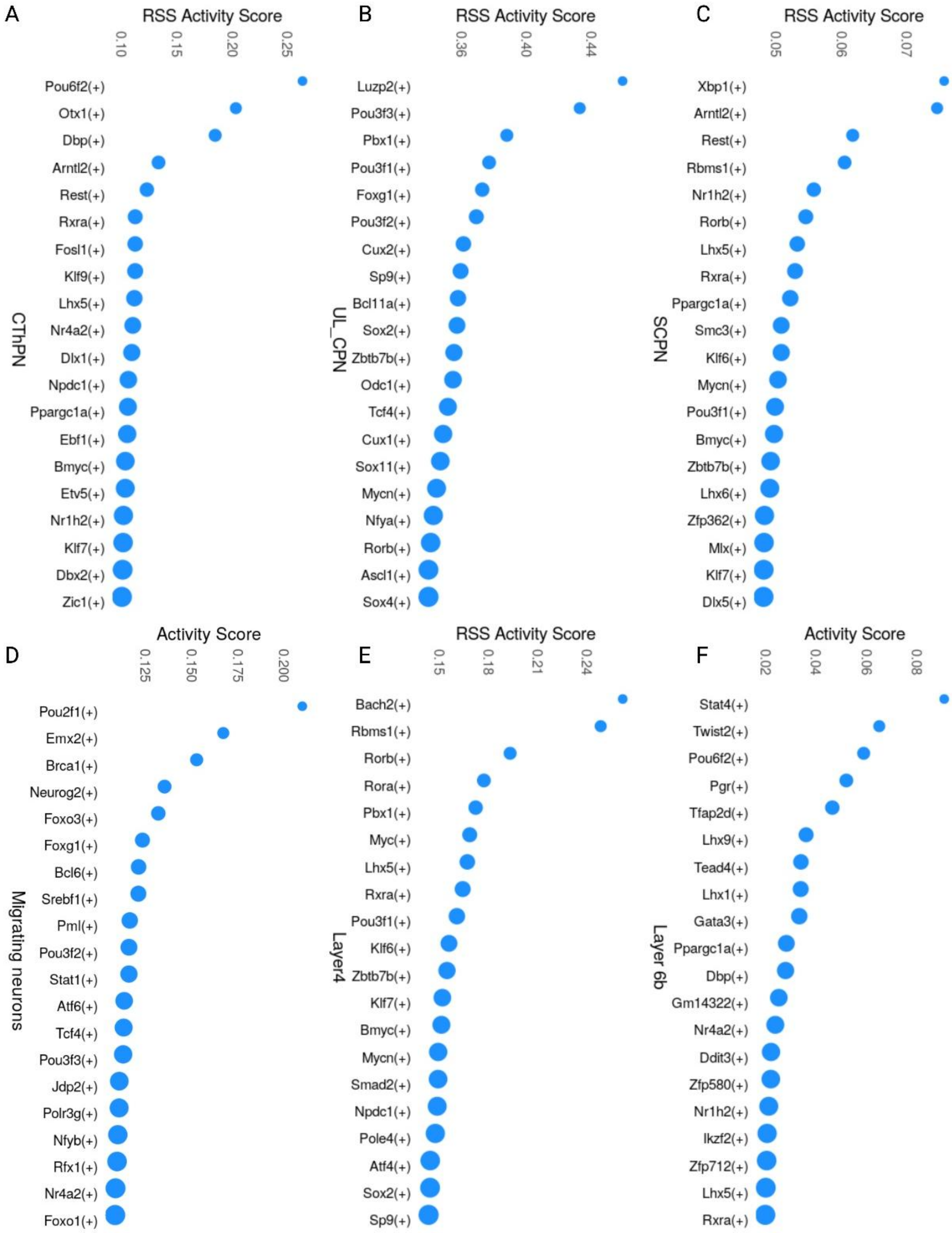

Supplementary Figure 7. Regulons defining mature neuronal subtypes at E18.5 (Part 3). (A–F) RSS analysis showing top regulons for (A) corticothalamic projection neurons (CThPN), (B) upper-layer CPNs (UL CPN), (C) subcerebral projection neurons (SCPN), (D) migrating neurons, (E) layer 4 neurons (Rorb), and (F) layer 6b neurons, capturing stage-specific transcriptional specialization.

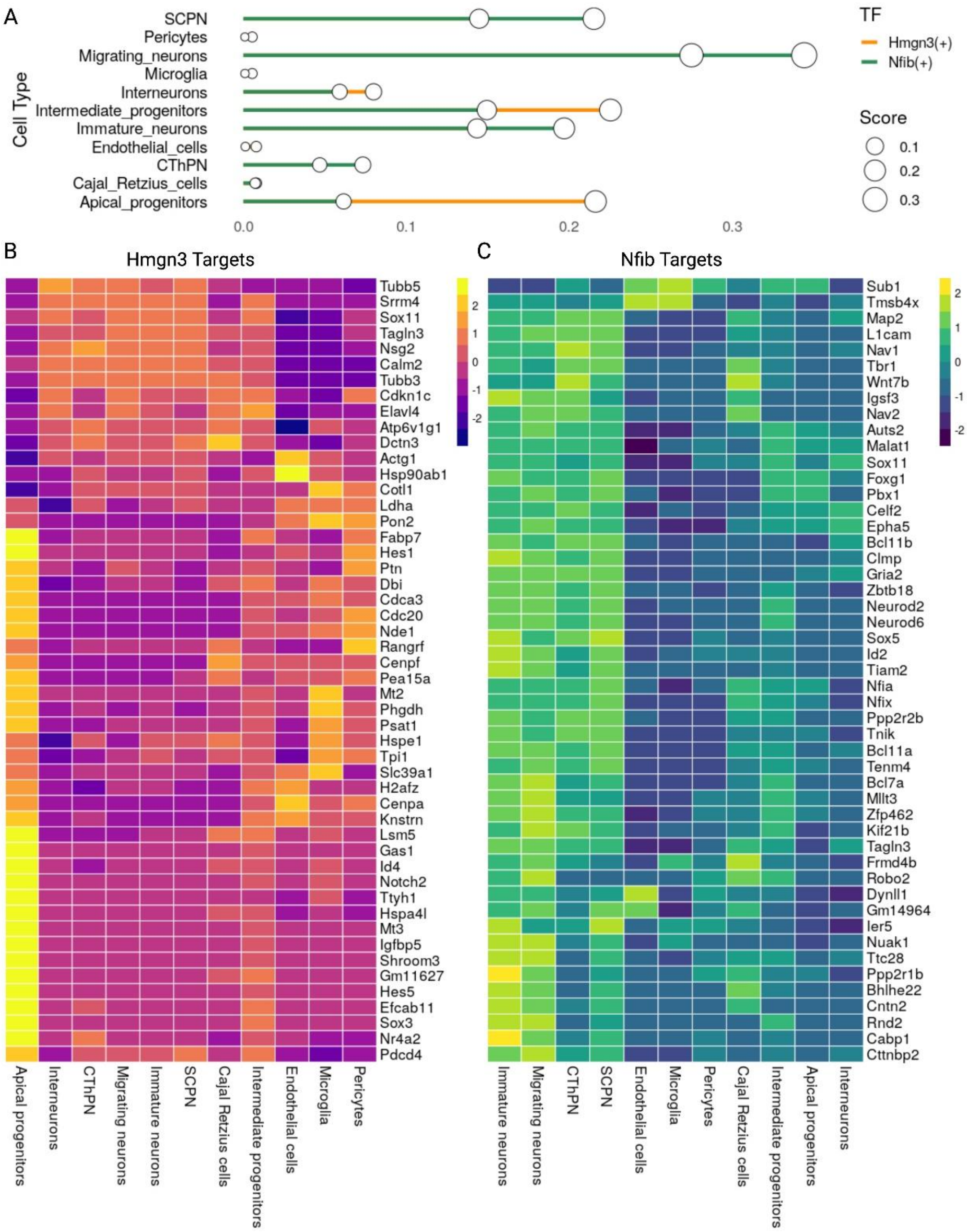

Supplementary Figure 8. Cell-type specificity of Hmgn3 and Nfib regulons during neurogenesis. (A) Bar plot of RSS scores showing Hmgn3 activity enriched in progenitors and Nfib in neuronal populations. (B–C) UMAP plots of aggregated target-gene expression for Hmgn3 (B) and Nfib (C), confirming co-expression of their regulons within expected cell types.

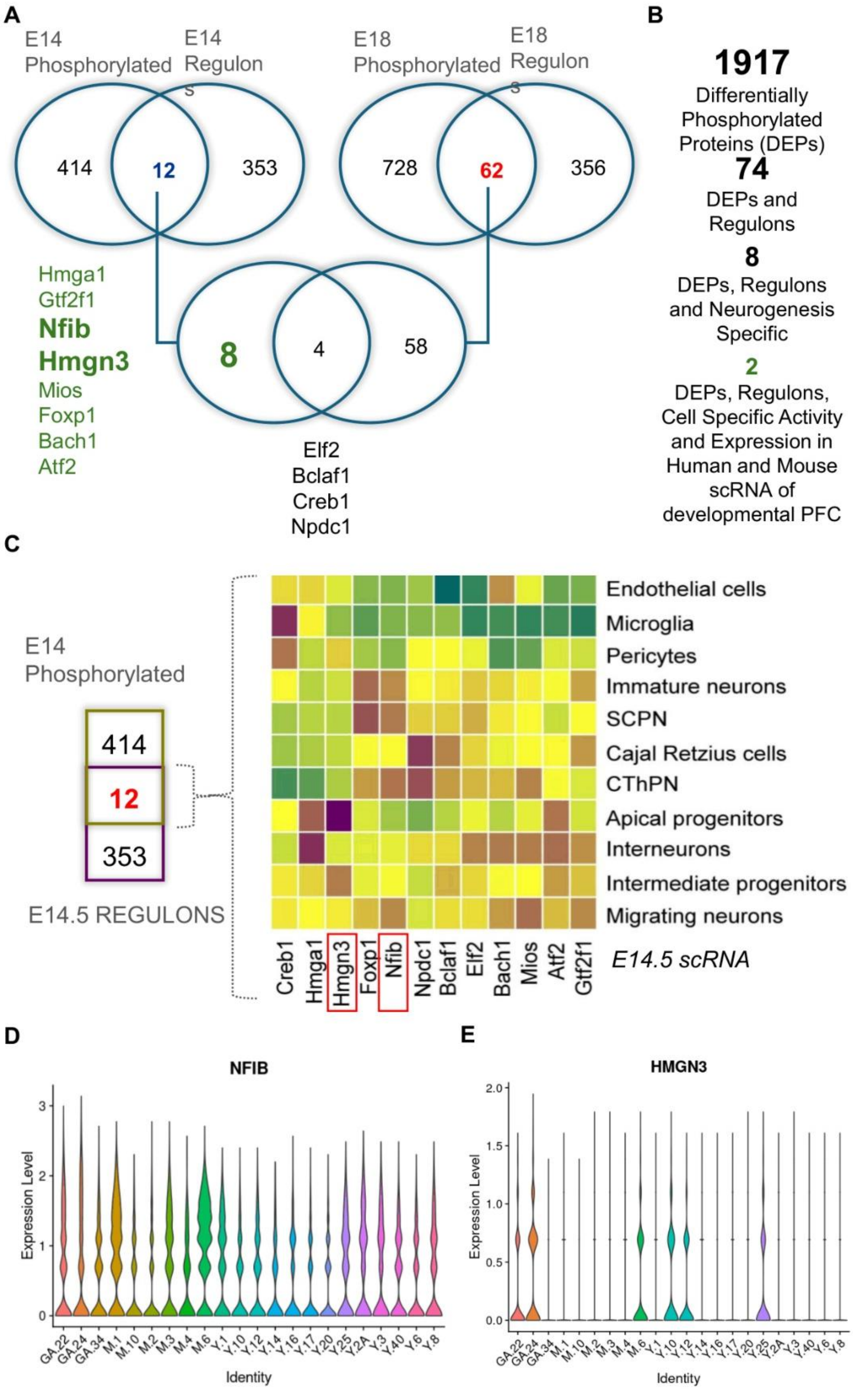

Supplementary Figure 9. Workflow for selection of Hmgn3 and Nfib for functional characterization. (A–B) Overlap between phosphorylated proteins and transcription factor (TF) regulons at E14.5 and E18.5, identifying 12 and 62 phosphorylated TFs, respectively. Comparison across stages shows shared (n = 8) and stage-specific candidates, including Hmga1, Gtf2f1, Nfib, Hmgn3, Mios, Foxp1, Bach1, and Atf2. (C) Heatmap showing expression of selected phosphorylated TFs across E14.5 single-cell RNA-seq cell types. (D–E) Violin plots

showing expression of NFIB (D) and HMGN3 (E) across developmental stages in prefrontal cortex single-cell RNA-seq data.

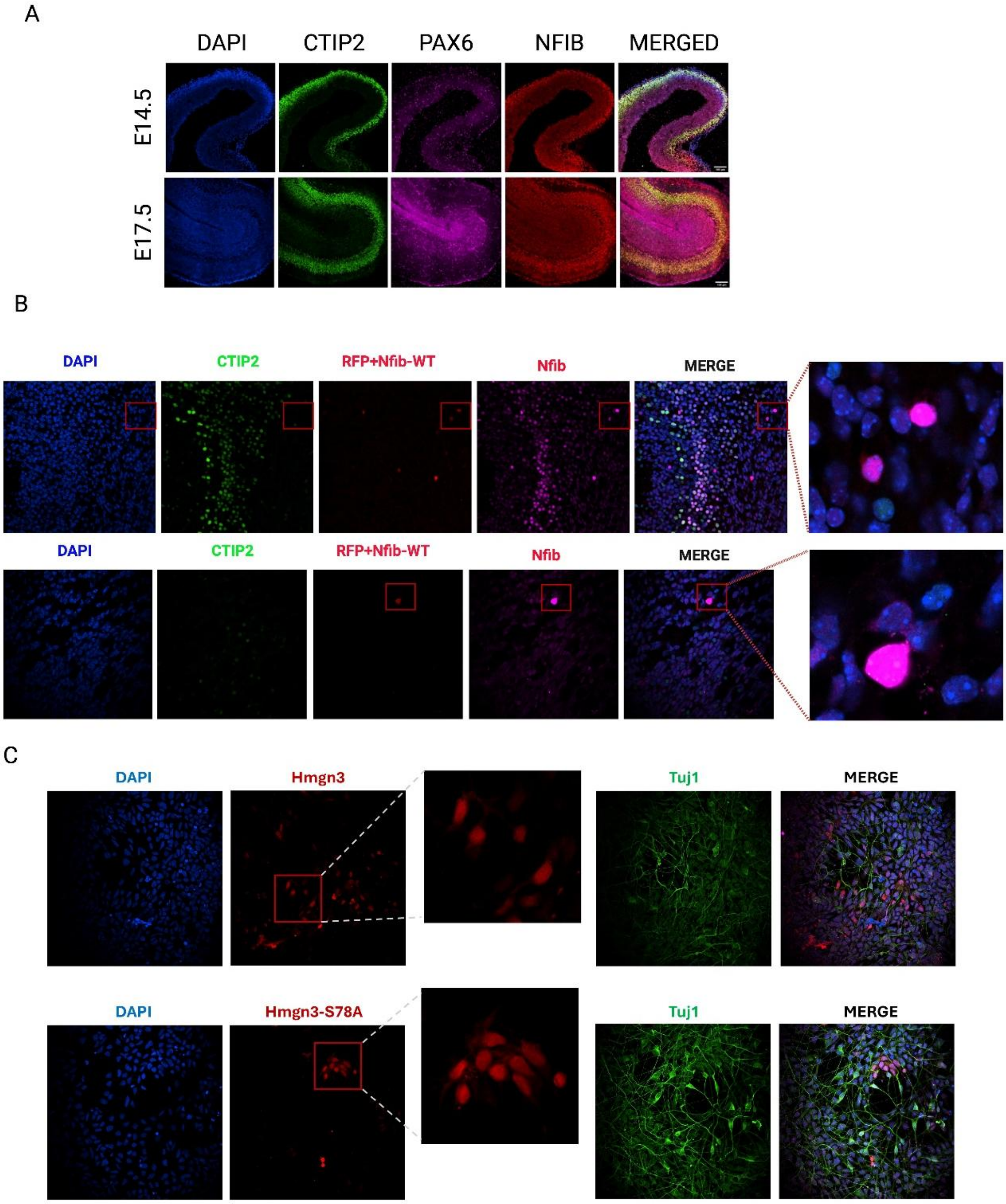

Supplementary Figure 10. Subcellular localization of Hmgn3 and Nfib phospho mutants. (A) Immunofluorescence showing endogenous Nfib expression in E14.5 and E17.5 cortex. (B) Loss of phosphorylation does not alter Nfib nuclear localization. (C) Similarly, Hmgn3 S78A mutation does not affect nuclear localization, confirming phosphorylation impacts function rather than subcellular distribution.

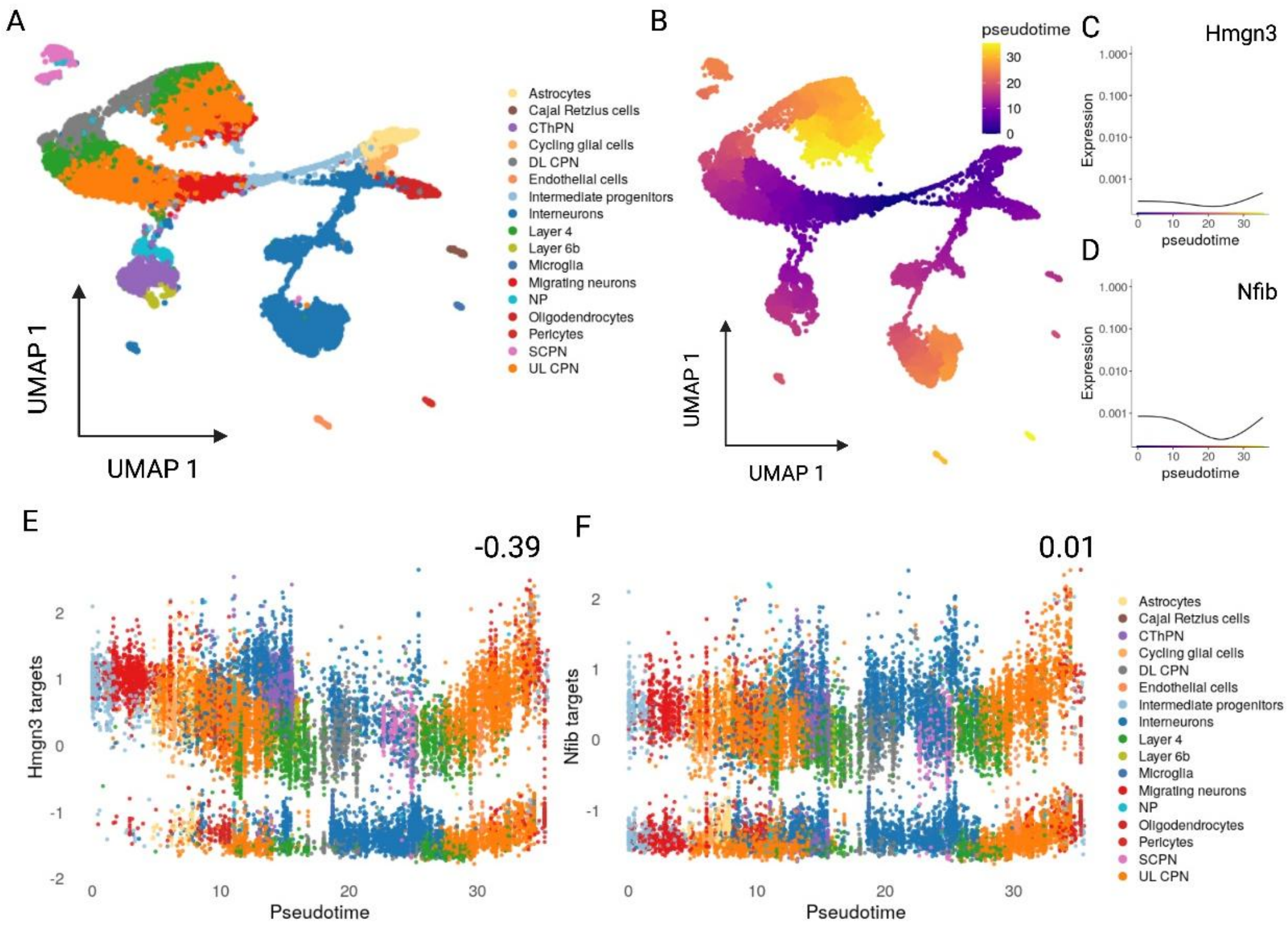

Supplementary Figure 11. Pseudotime dynamics of Hmgn3 and Nfib during cortical development at E18.5. (A) UMAP representation of E18.5 single-cell RNA-seq data colored by annotated cell types. (B) UMAP colored by pseudotime, illustrating developmental progression from progenitors to differentiated cell populations. (C–D) Expression of Hmgn3 (C) and Nfib (D) along pseudotime. (E–F) Expression of Hmgn3 (E) and Nfib. (F) target gene modules plotted across pseudotime (Hmgn3: r = −0.39; Nfib: r = 0.01).

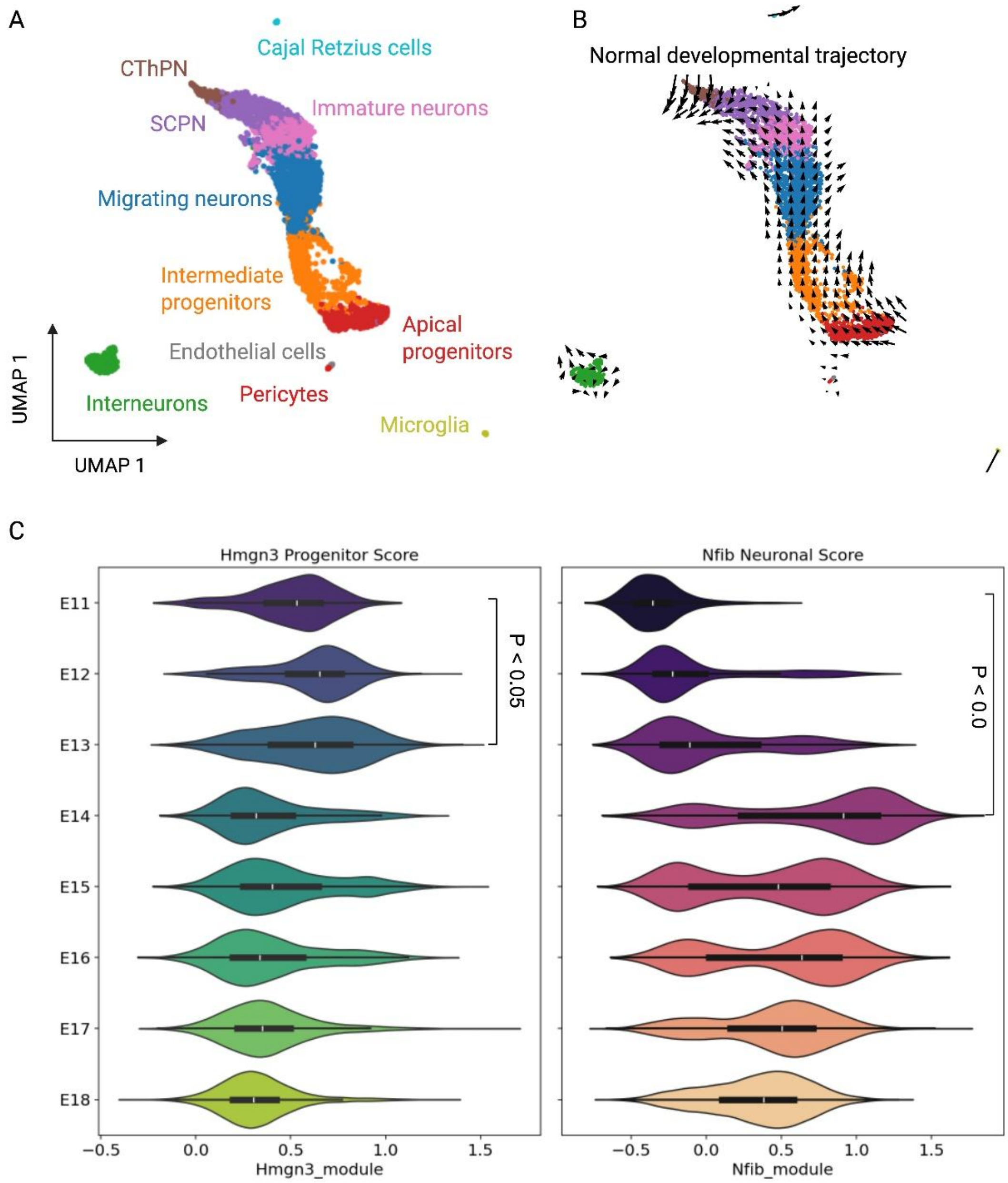

Supplementary Figure 12. Trajectory analysis and *in silico* perturbation of neurogenesis. (A) UMAP representation of neurogenic cell populations. (B) Developmental trajectory overlay illustrating progressive differentiation from progenitors through neuronal maturation used for perturbation modeling. (C) Hmgn3 (left) and Nfib (right) target genes' module score through each developmental stage (E11-E18). Hmgn3 targets received highest score at E13; Nfib targets received highest score at E14.

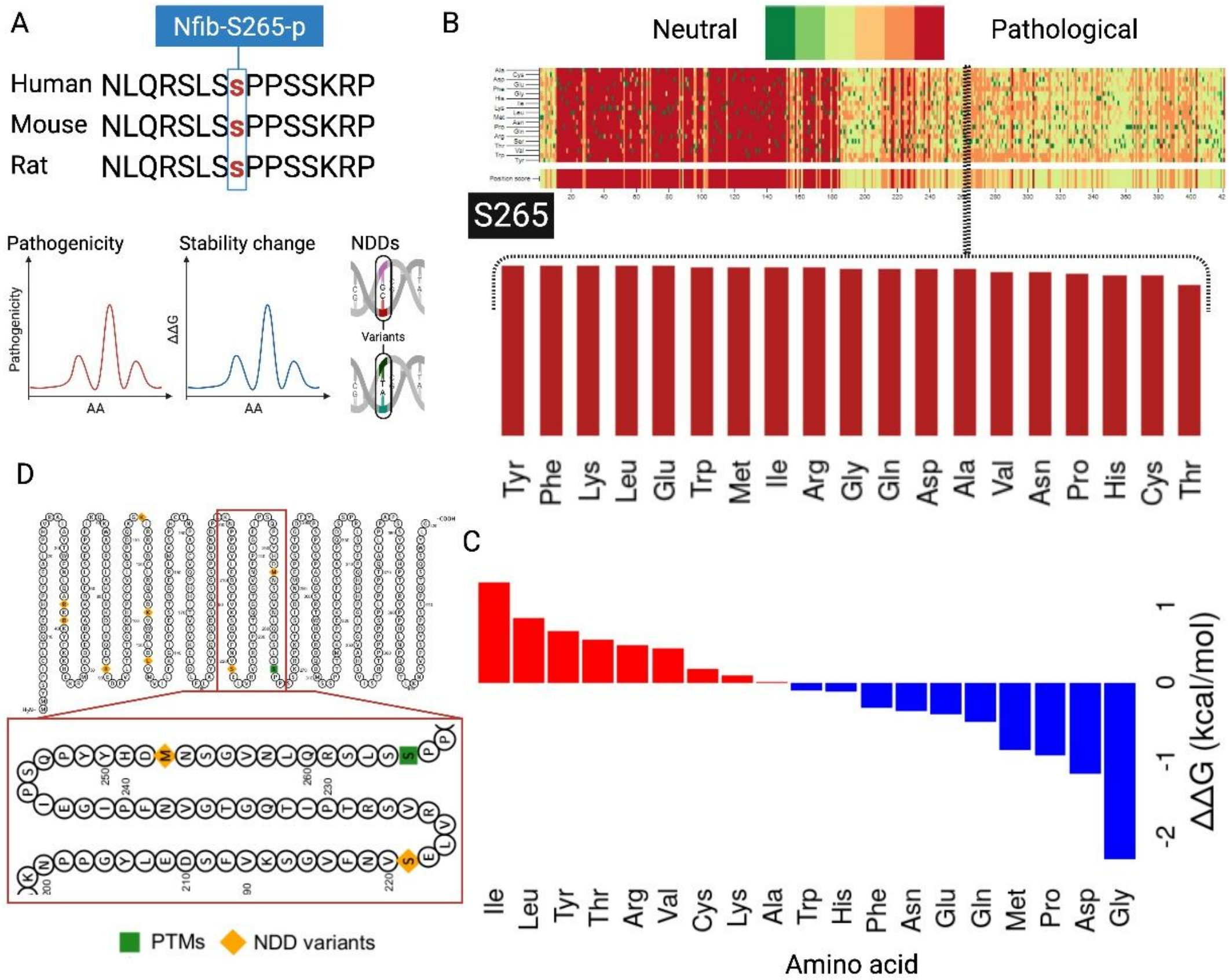

Supplementary Figure 13. Computational prediction of pathogenicity at Nfib S265. (A) Schematic of Nfib protein denoting S265 phosphosite. (B) Pathogenicity scores (CADD) for possible amino acid substitutions. (C) Predicted effects on protein stability (ΔΔG). (D) Position of human neurodevelopmental disorder (NDD) variants surrounding S265. Together, analyses identify S265 as a conserved, functionally critical site.

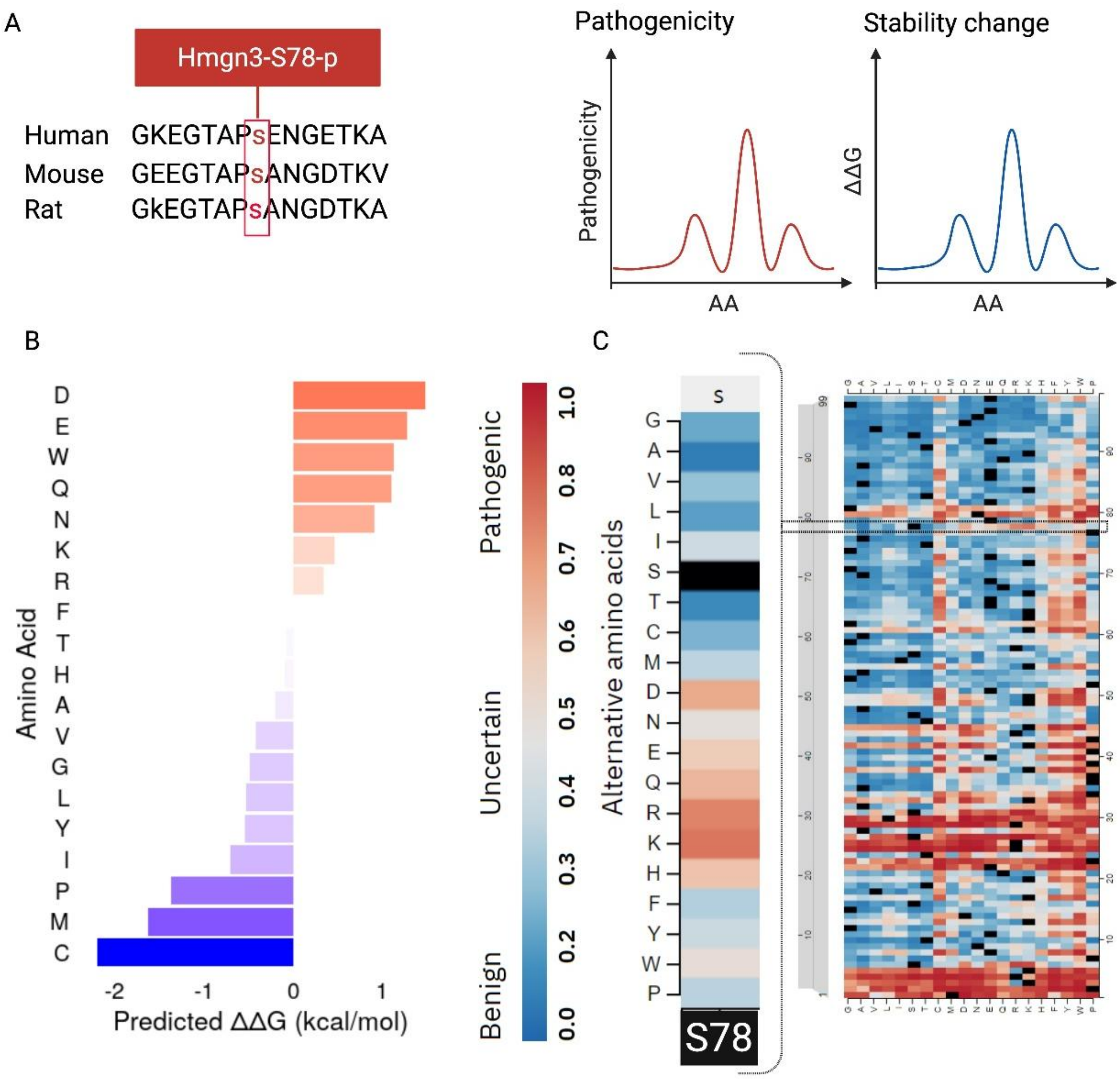

Supplementary Figure 14. Computational analysis of the Hmgn3 S78 phosphosite. (A) Cross-species conservation of S78 in Hmgn3. (B) Predicted effects of substitutions on protein stability (ΔΔG). (C) Pathogenicity scores for S78 variants, supporting functional importance of this residue in structural integrity and activity.

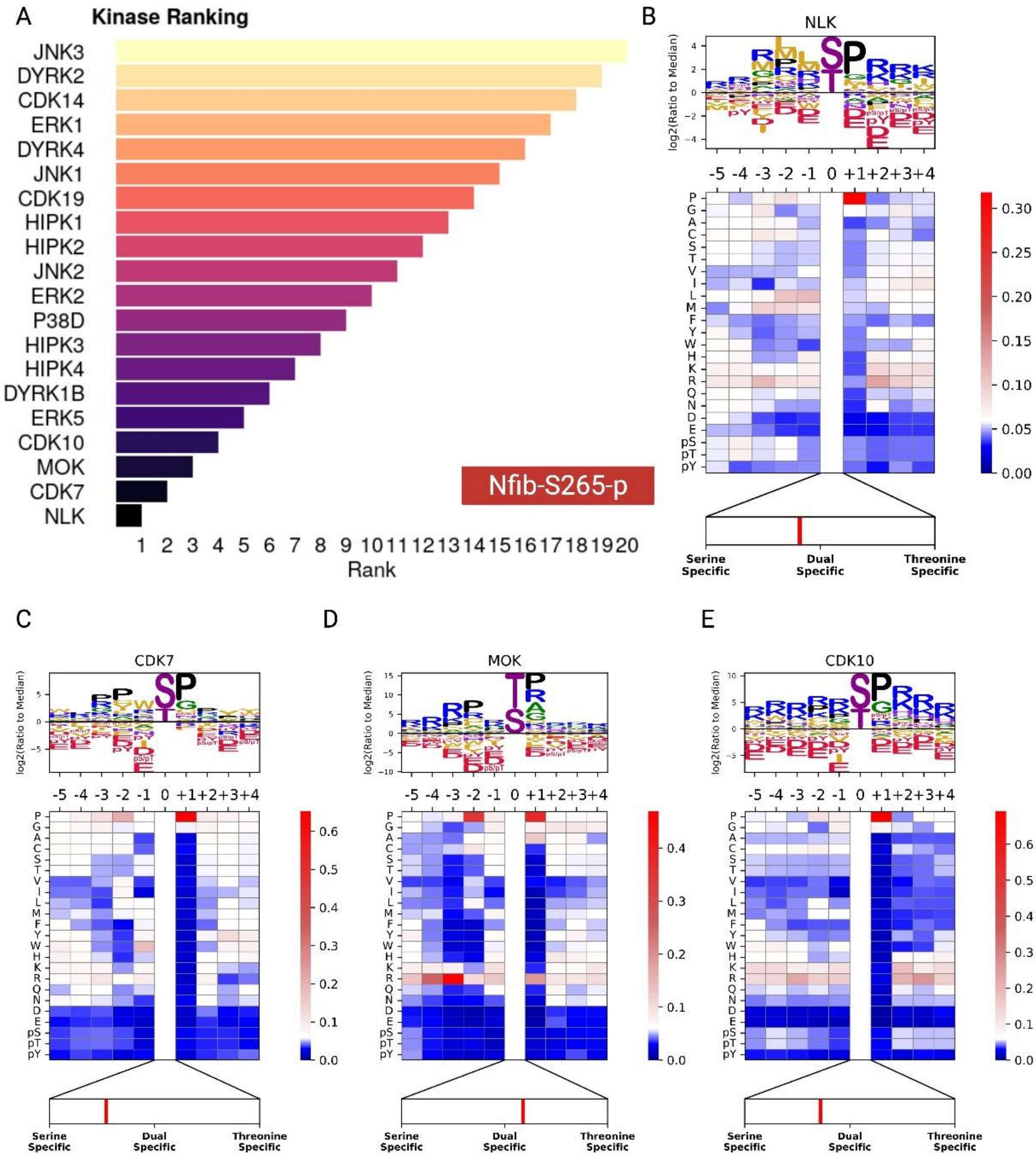

Supplementary Figure 15. Prediction of upstream kinases targeting Nfib S265. (A) Top 20 candidate kinases ranked by motif scoring. (B–E) Consensus motif logos for top candidates NLK (B), CDK7 (C), MOK (D), CDK10 (E) showing sequence similarity to the S265 flanking region.

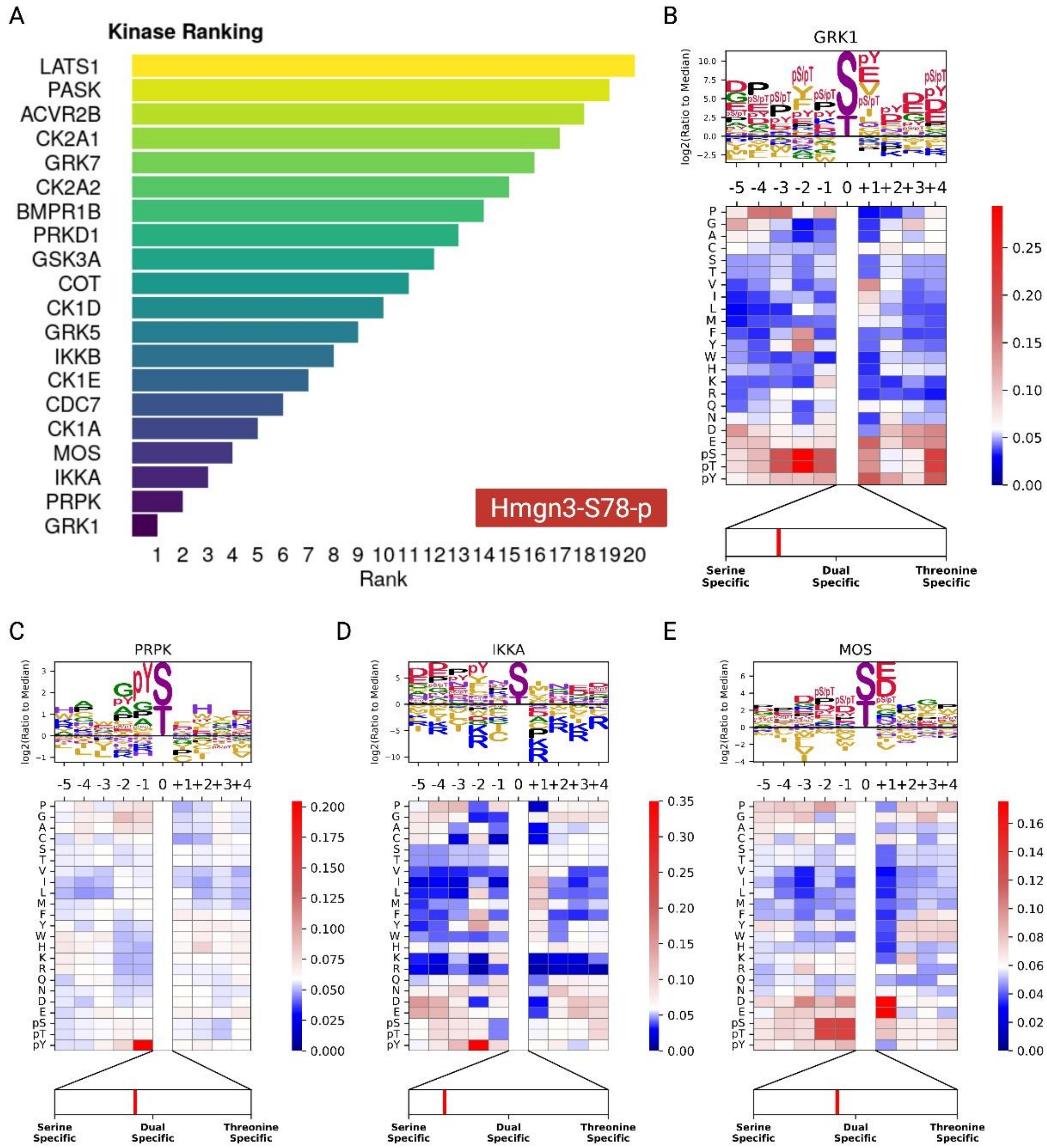

Supplementary Figure 16. Prediction of upstream kinases targeting Hmgn3 S78. (A) Top 20 candidate kinases predicted for Hmgn3 S78. (B–E) Motif logos for leading candidates GRK1 (B), PRPK (C), IKKα (D), and MOS (E), supporting potential kinase recognition near S78.

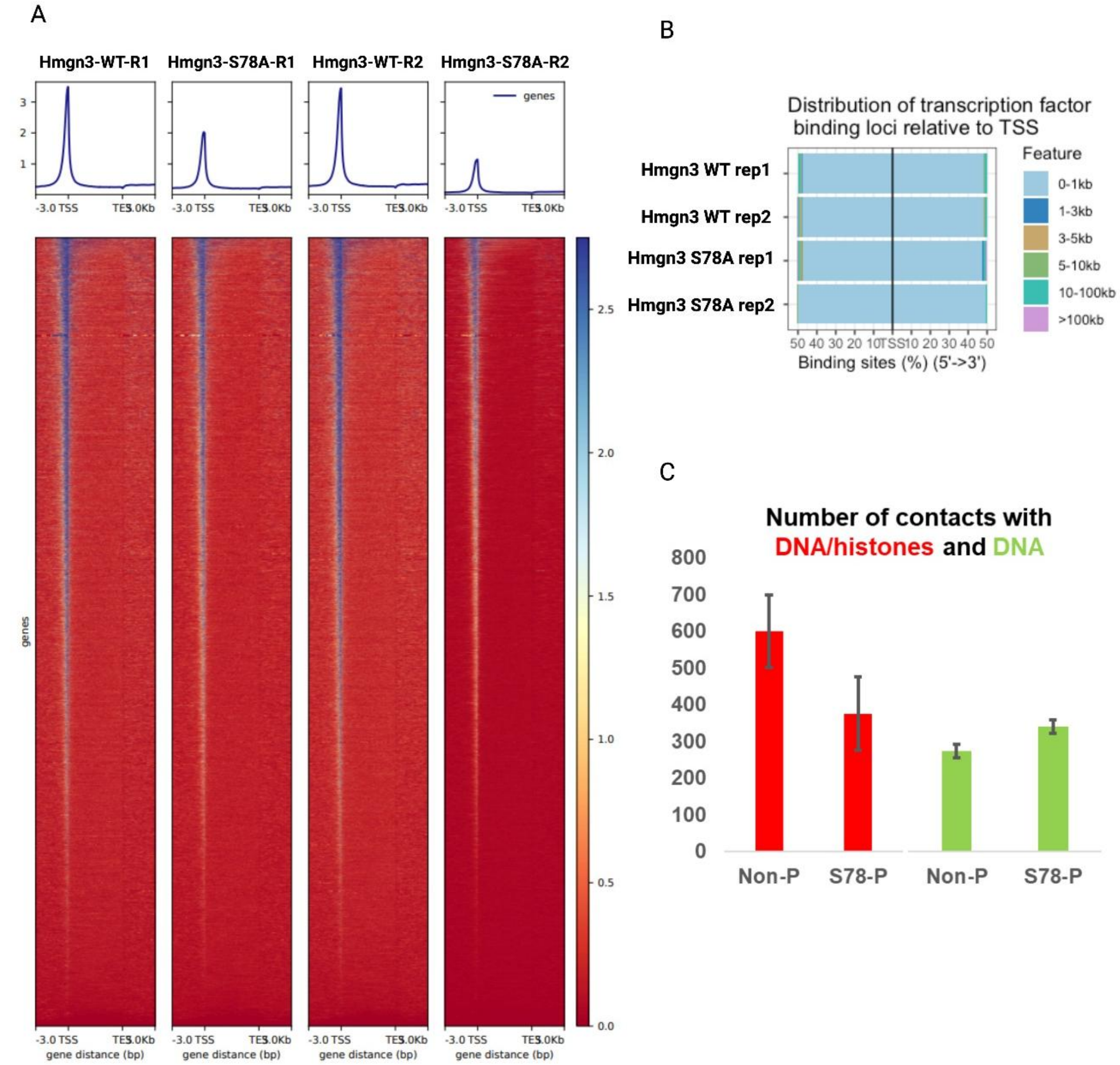

Supplementary Figure 17. Genomic distribution of Hmgn3 binding sites. (A) CUT&RUN profiles of Hmgn3-WT and HMGN3-S78A demonstrating overall binding distribution across genomic regions. (B) Quantitative distribution of Hmgn3 peaks relative to transcription start sites (TSS). Phosphorylation at S78 does not alter genomic preference but impacts binding strength. (C) The number of contacts with DNA (green) and contacts between DNA and histones (red) in non- and phosphorylated Hmgn3.

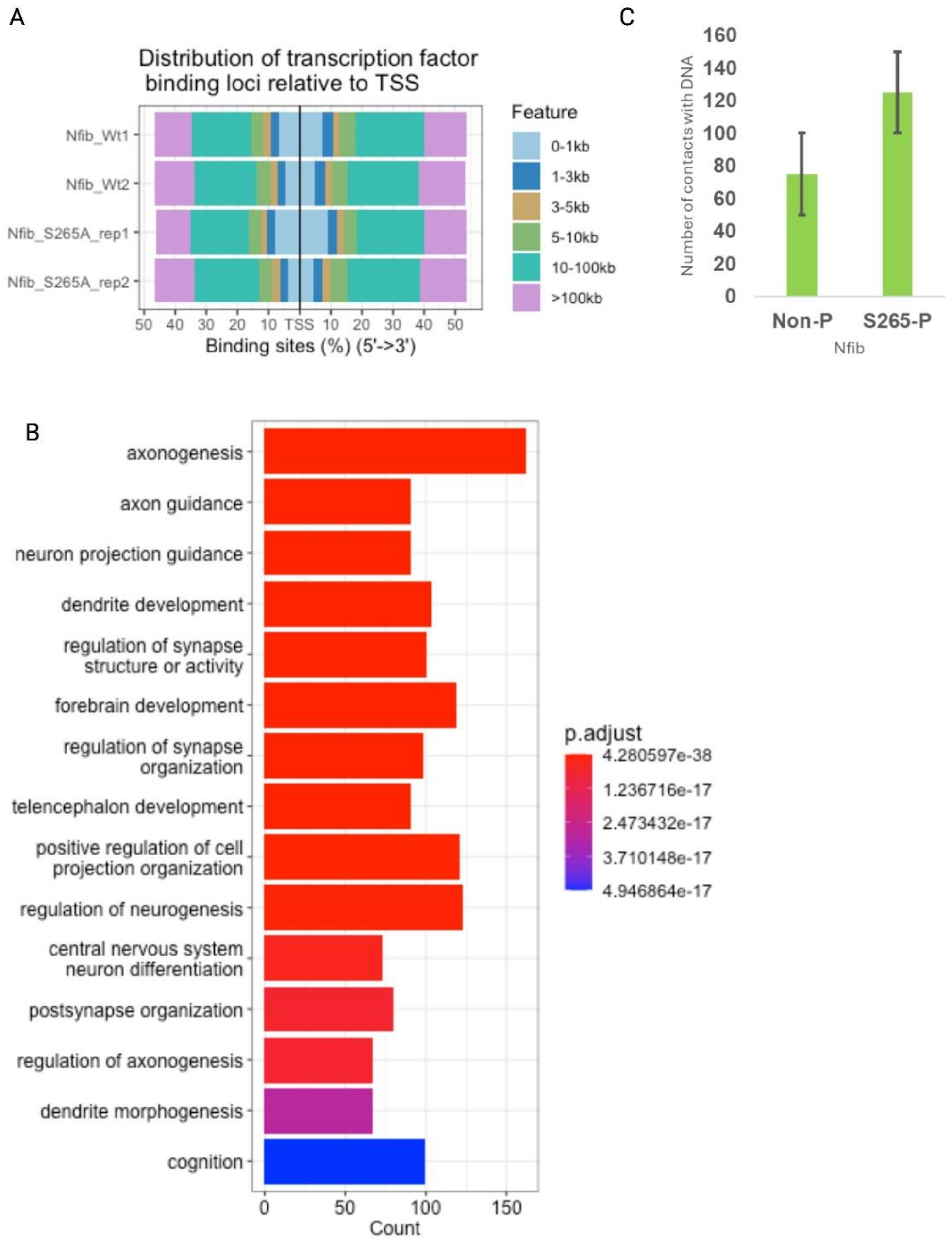

Supplementary Figure 18. Genomic distribution of Nfib binding sites and functional annotation. (A) Distribution of Nfib binding peaks across genomic features showing predominant enrichment at distal regulatory elements. (B) GO analysis of genes near promoter-proximal Nfib peaks highlights significant enrichment of axonogenesis and neuronal development pathways. (C) The number of contacts with DNA in non- and phosphorylated Nfib.

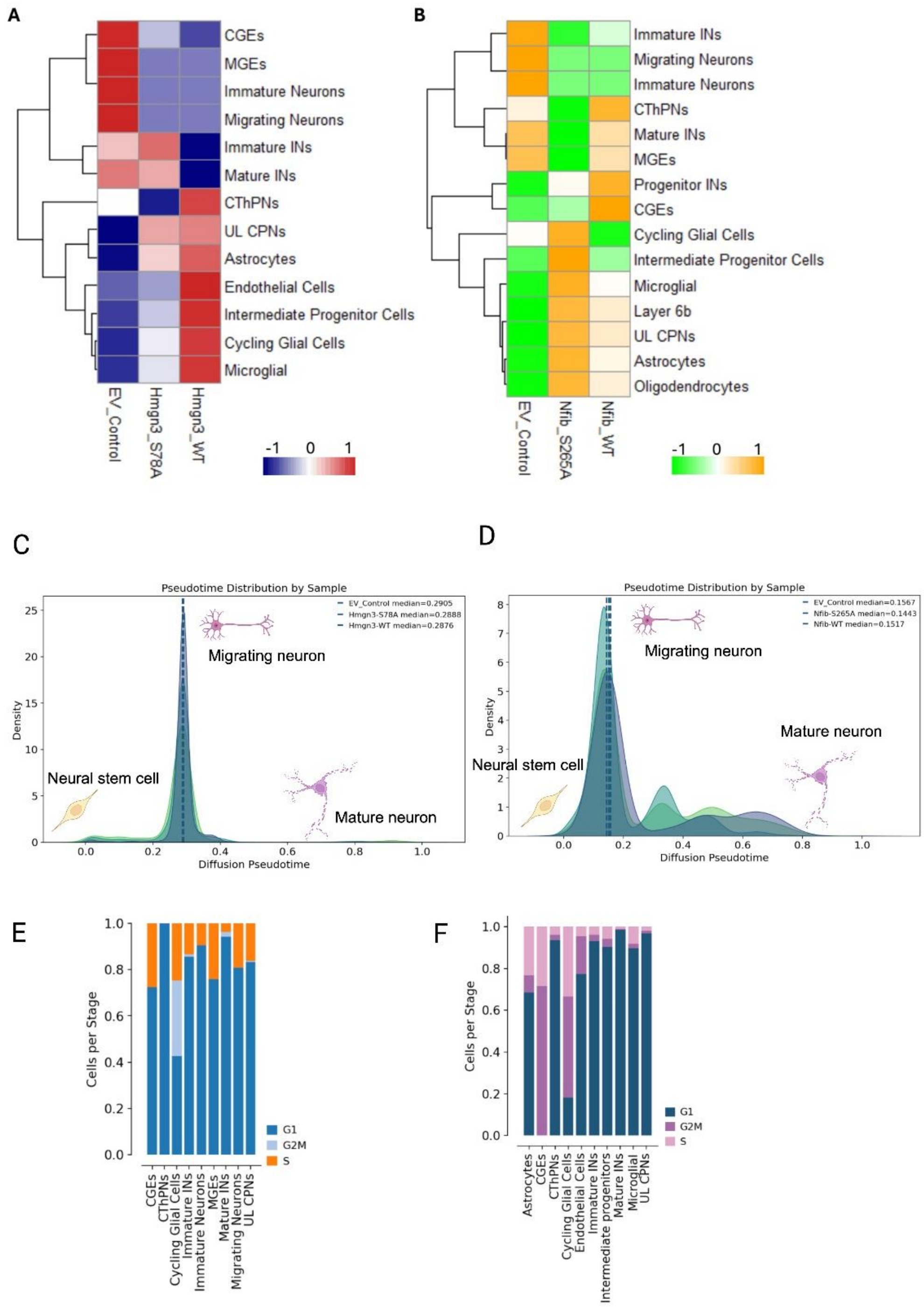

Supplementary Figure 19. Phosphorylation-dependent effects of Hmgn3 and Nfib on cell-type composition. (A) Heatmap showing relative abundance (row-normalized, z-scored) of cell types across control (EV), Hmgn3-S78A, and Hmgn3-WT conditions following single-cell RNA-seq analysis. Rows represent annotated cell populations and columns represent experimental conditions; hierarchical clustering is shown. (B) Heatmap showing relative abundance of cell types across control (EV), Nfib-S265A, and Nfib-WT conditions. Rows represent annotated cell populations and columns represent experimental conditions; hierarchical clustering is shown. (C) The pseudotime of Hmgn3-WT and Hmgn3-S78A shift the neural lineage compared with EV control from IUE-scRNA-Seq data. (D) The pseudotime

of Nfib-WT and Nfib-S265A shift the neural lineage compared with EV control from IUE-scRNA-Seq data. (E, F) Cell cycle stages from IUE-scRNA-Seq EV control and Hmgn3, the G2M phase increased from 31% (EV) to 44% (Hmgn3). Created in BioRender. Lusby, R. (2026) https://BioRender.com/b5m9jk2

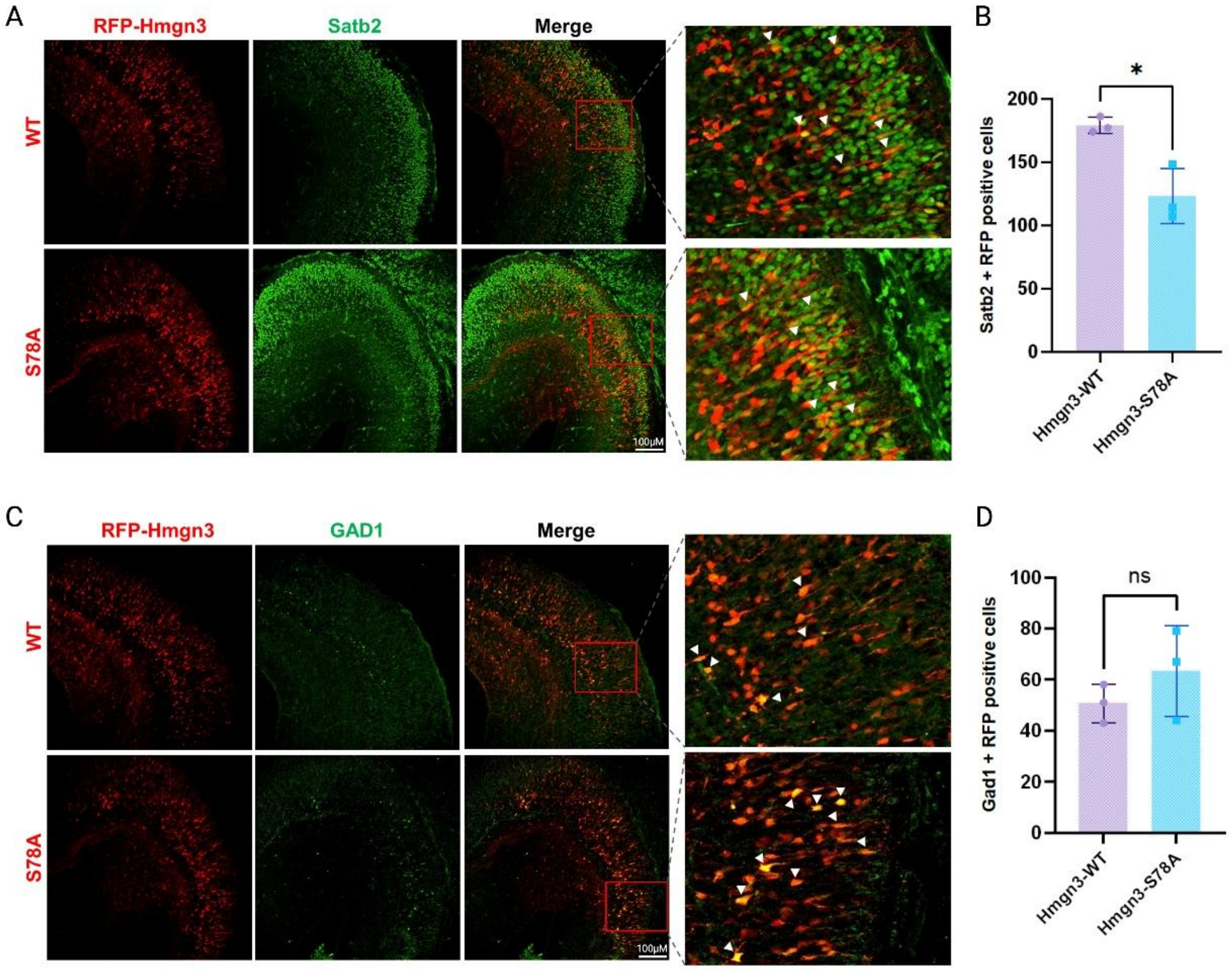

Supplementary Figure 20. Hmgn3 phosphorylation alters neuronal specifications (A-B) Immunofluorescence images stained with Satb2 showing confirmation of upper-layer neuronal cell populations generated following overexpression of wild-type and phospho-mutant of Hmgn3 (P = 0.039). (C-D) Immunofluorescence images show changes in interneuron cell populations stained with Gad1 following overexpression of wild-type and phospho-mutant of Hmgn3, with their quantification (RFP+ Gad1+ cells) represented in the bar graph (P = 0.34). Scale bars: 100 µm. Statistical significance was calculated using a Welch's two-tailed *t*-test. **P*<0.05, ***P*<0.01, ns: not significant; *n*=3 independent biological replicates. Data are mean±s.d. Created in BioRender. Lusby, R. (2026) https://BioRender.com/2hmzh6y

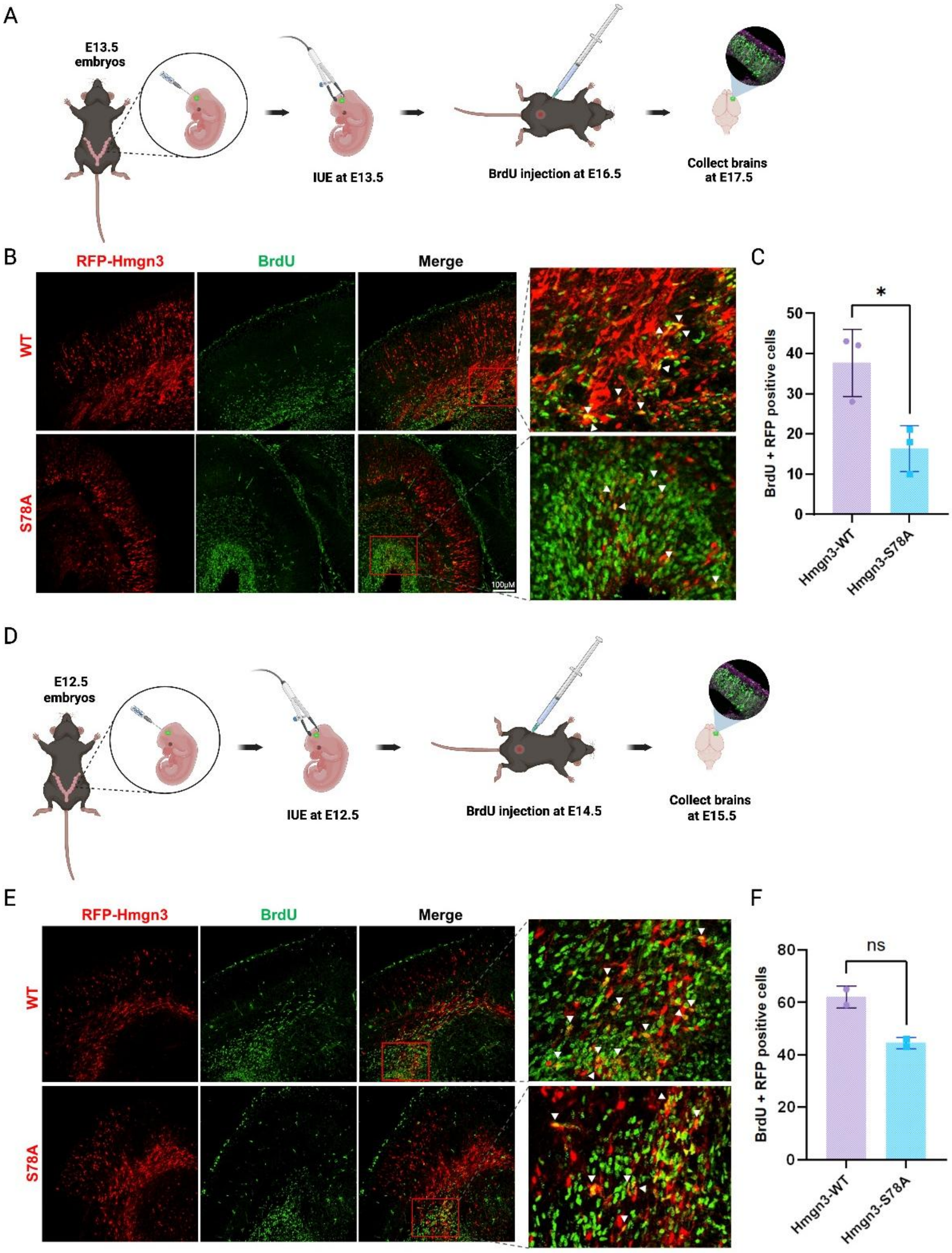

Supplementary Figure 21. BrdU incorporation analysis reveals progenitor expansion role of Hmgn3 (A) Experimental layout for the in-utero electroporation (IUE) assay, where IUE was carried out at E13.5, followed by BrdU administration at E16.5, and analysed at E17.5. (B-C) Immunofluorescence images showing changes in proliferative cell populations stained with BrdU following overexpression of wild-type and phospho-mutant of Hmgn3, with their quantification (RFP+ BrdU+ cells) represented in the bar graph (P = 0.027). (D) Experimental layout for the in-utero electroporation (IUE) assay, where IUE was carried out at E12.5, followed by BrdU administration at E14.5, and analysed at E15.5.(E-F) Immunofluorescence images showing changes in proliferative cell populations stained with BrdU following overexpression of wild-type and phospho-mutant of Hmgn3, with their quantification (RFP+

BrdU+ cells) represented in the bar graph ($P = 0.06$). Scale bars: 100 µm. Statistical significance was calculated using a Welch's two-tailed *t*-test. *$P<0.05$, **$P<0.01$, ns: not significant; $n=3$ independent biological replicates. Data are mean±s.d. Created in BioRender. Lusby, R. (2026) https://BioRender.com/534d30k

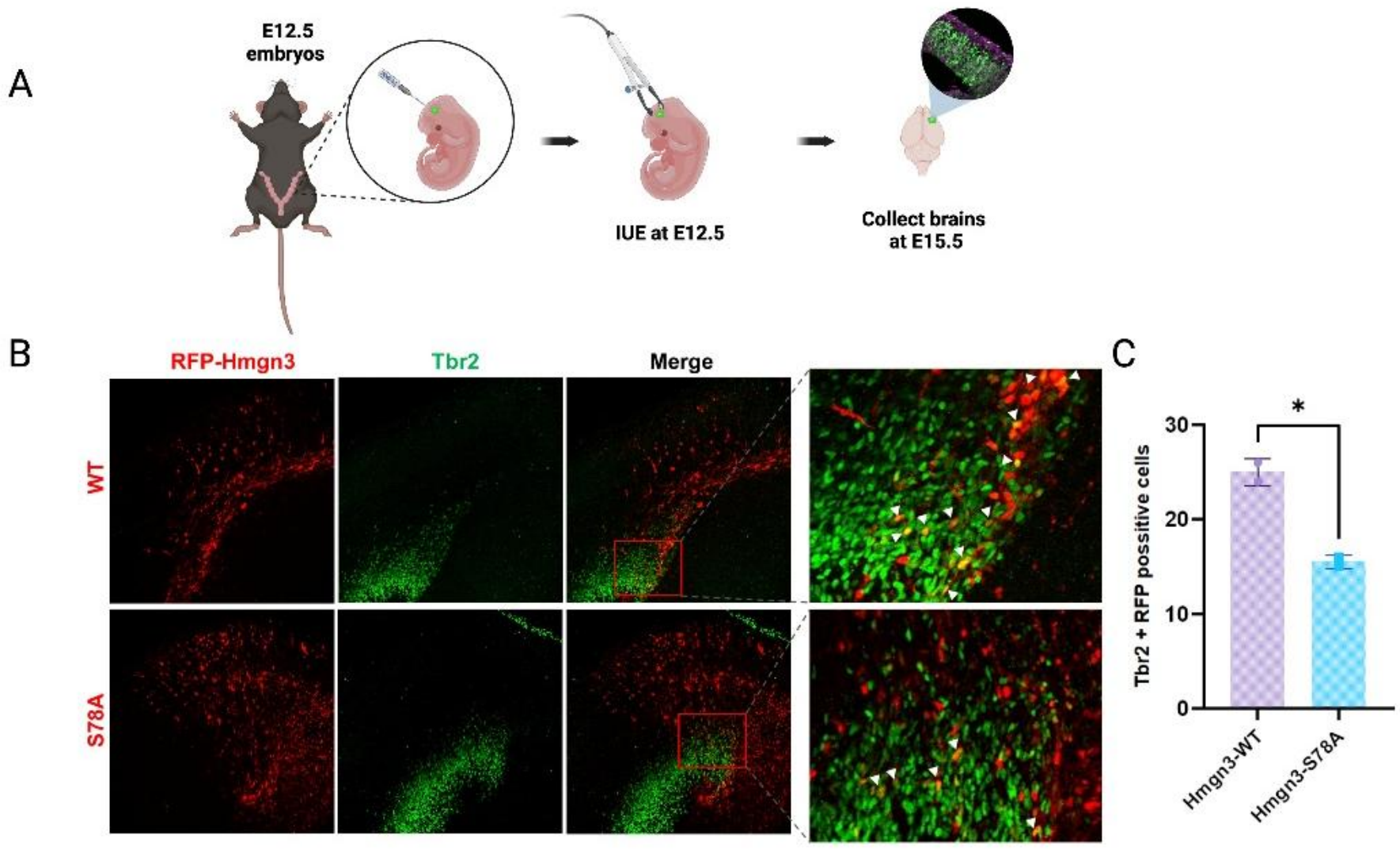

Supplementary Figure 22. Hmgn3 phosphorylation alters progenitor populations. (A) Experimental lectroporation (IUE) assay where IUE was carried out at E12.5 and analysed at E15.5. (B-C) Immunofluorescence images showing changes in intermediate progenitor cell populations stained with Tbr2 following overexpression of wild-type and phospho-mutant of Hmgn3, with their quantification (RFP+ Tbr2+ cells) represented in the bar graph (P = 0.031). Scale bars: 100 µm. Statistical significance was calculated using a Welch's two-tailed *t*-test. **P*<0.05, ***P*<0.01, ns: not significant; *n*=2 independent biological replicates. Data are mean±s.d. Created in BioRender. Lusby, R. (2026) https://BioRender.com/2hmzh6y

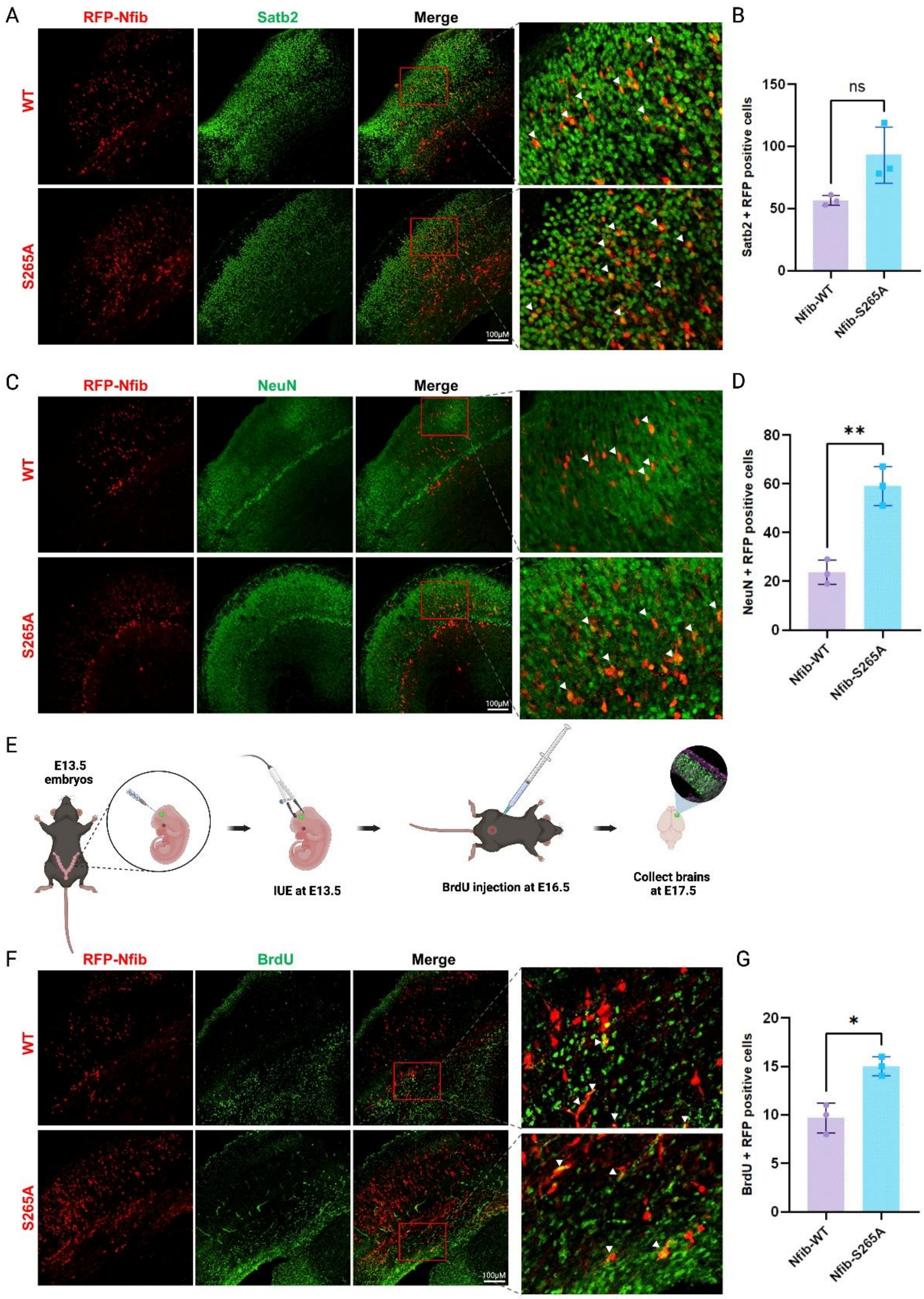

Supplementary Figure 23. Nfib phosphorylation affects neuronal fate. (A-B) Immunofluorescence images stained with Satb2 showing confirmation of upper-layer neuronal cell populations generated following overexpression of wild-type and phospho-mutant of Nfib (P = 0.104). (C-D) Immunofluorescence images stained with NeuN showing confirmation of neuronal cell populations generated following overexpression of wild-type and phospho-mutant of Nfib (P = 0.0052). (E) Experimental layout for the in-utero electroporation (IUE) assay, where IUE was carried out at E13.5, followed by BrdU administration at E16.5, and

analysed at E17.5. (F-G) Immunofluorescence images showing changes in proliferative cell populations stained with BrdU following overexpression of wild-type and phospho-mutant of Nfib, with their quantification (RFP+ BrdU+ cells) represented in the bar graph ($P = 0.010$). Scale bars: 100 μm. Statistical significance was calculated using a Welch's two-tailed *t*-test. *$P<0.05$, **$P<0.01$, ns: not significant; $n=3$ independent biological replicates. Data are mean±s.d. Created in BioRender. Lusby, R. (2026) https://BioRender.com/axe0i6e
