# Supplementary material for: Phosphorylation-dependent control of transcription factor activity regulates temporal patterning of cortical cell fate specification
Source: Nat Commun. 2026 Sep 4;17:10605. doi: 10.1038/s41467-026-77195-8 (PMC13645745; doi:10.1038/s41467-026-77195-8)
Supplement: Supplementary file 2 [file 41467_2026_77195_MOESM2_ESM.pdf]

Description of Additional Supplementary Files

File Name: Supplementary Data 1

Description: Differentially phosphorylated proteins

File Name: Supplementary Data 2

Description: Hmgn3 WT CUT&RUN rep1

File Name: Supplementary Data 3

Description: Hmgn3 WT CUT&RUN rep2

File Name: Supplementary Data 4

Description: Hmgn3 S78A CUT&RUN rep1

File Name: Supplementary Data 5

Description: Hmgn3 S78A CUT&RUN rep2

File Name: Supplementary Data 6

Description: Common genes in WT and Mutant

File Name: Supplementary Data 7

Description: Nfib WT CUT&RUN rep1

File Name: Supplementary Data 8

Description: Nfib WT CUT&RUN rep2

File Name: Supplementary Data 9

Description: Nfib S265A CUT&RUN rep1

File Name: Supplementary Data 10

Description: Nfib S265A CUT&RUN rep2

File Name: Supplementary Data 11

Description: IUE-scRNA-Seq quantifications

File Name: Supplementary Data 12

Description: IUE cell types quantifications
