# Supplementary material for: Phosphorylation-dependent control of transcription factor activity regulates temporal patterning of cortical cell fate specification
Source: Nat Commun. 2026 Sep 4;17:10605. doi: 10.1038/s41467-026-77195-8 (PMC13645745; doi:10.1038/s41467-026-77195-8)
Supplement: Supplementary file 3 [file 41467_2026_77195_MOESM4_ESM.pdf]

nature portfolio

Corresponding author(s): NCOMMS-26-001307A

Last updated by author(s): Jun 21, 2026

Reporting Summary

Nature Portfolio wishes to improve the reproducibility of the work that we publish. This form provides structure for consistency and transparency in reporting. For further information on Nature Portfolio policies, see our Editorial Policies and the Editorial Policy Checklist.

Statistics

For all statistical analyses, confirm that the following items are present in the figure legend, table legend, main text, or Methods section.

n/a	Confirmed	
☐	☒	The exact sample size (n) for each experimental group/condition, given as a discrete number and unit of measurement
☐	☒	A statement on whether measurements were taken from distinct samples or whether the same sample was measured repeatedly
☐	☒	The statistical test(s) used AND whether they are one- or two-sided<br>*Only common tests should be described solely by name; describe more complex techniques in the Methods section.*
☐	☒	A description of all covariates tested
☐	☒	A description of any assumptions or corrections, such as tests of normality and adjustment for multiple comparisons
☐	☒	A full description of the statistical parameters including central tendency (e.g. means) or other basic estimates (e.g. regression coefficient) AND variation (e.g. standard deviation) or associated estimates of uncertainty (e.g. confidence intervals)
☐	☒	For null hypothesis testing, the test statistic (e.g. F, t, r) with confidence intervals, effect sizes, degrees of freedom and P value noted<br>*Give P values as exact values whenever suitable.*
☒	☐	For Bayesian analysis, information on the choice of priors and Markov chain Monte Carlo settings
☒	☐	For hierarchical and complex designs, identification of the appropriate level for tests and full reporting of outcomes
☐	☒	Estimates of effect sizes (e.g. Cohen's d, Pearson's r), indicating how they were calculated

Our web collection on statistics for biologists contains articles on many of the points above.

Software and code

Policy information about availability of computer code

Data collection	Data collection for this study was performed using established experimental protocols and commercially available platforms, including tandem mass tag (TMT)-based proteomics, RNA sequencing, CUT&RUN, and single-cell RNA sequencing (Parse Biosciences). All datasets were generated using standard laboratory and sequencing methodologies as described in the Methods. No custom code was generated for data collection.<br>scRNA-seq (E10.5-P4) Public PMID: 34163074<br>scATAC-seq (E13.5-E18.5) Public PMID: 34163074<br>Proteome/Phosphoproteome This study PXD066619<br>Bulk RNA-seq This study GSE302501<br>CUT&RUN This study GSE327199<br>Parse scRNA-seq (IUE) This study GSE327212
Data analysis	All data analyses were performed using established, publicly available software and computational tools, including MaxQuant for proteomics and phosphoproteomics, Seurat for single-cell RNA-seq analysis, PySCENIC for gene regulatory network inference, and standard pipelines for bulk RNA-seq and CUT&RUN data processing. Default or previously published parameters were used unless otherwise specified in the Methods. No custom code was generated for data analysis.

For manuscripts utilizing custom algorithms or software that are central to the research but not yet described in published literature, software must be made available to editors and reviewers. We strongly encourage code deposition in a community repository (e.g. GitHub). See the Nature Portfolio guidelines for submitting code & software for further information.

Data

Policy information about availability of data

All manuscripts must include a data availability statement. This statement should provide the following information, where applicable:

- Accession codes, unique identifiers, or web links for publicly available datasets
- A description of any restrictions on data availability
- For clinical datasets or third party data, please ensure that the statement adheres to our policy

All datasets generated in this study are publicly available. Bulk RNA sequencing data generated during this study have been deposited in the Gene Expression Omnibus (GEO) under accession number GSE302501. Proteome and phosphoproteome datasets are available via the ProteomeXchange Consortium under accession number PXD066619. CUT&RUN sequencing data have been deposited in GEO under accession number GSE327199, and single-cell RNA-seq data from in utero electroporation experiments (Parse Biosciences platform) are available under accession number GSE327212. Previously published datasets used in this study include single-cell RNA-seq and single-cell ATAC-seq datasets of mouse cortical development (E10.5–P4 and E13.5–E18.5, respectively), available under GSE153164. All other data supporting the findings of this study are available from the corresponding author upon reasonable request.

Research involving human participants, their data, or biological material

Policy information about studies with human participants or human data. See also policy information about sex, gender (identity/presentation), and sexual orientation and race, ethnicity and racism.

Reporting on sex and gender	NA
Reporting on race, ethnicity, or other socially relevant groupings	NA
Population characteristics	NA
Recruitment	NA
Ethics oversight	NA

Note that full information on the approval of the study protocol must also be provided in the manuscript.

Field-specific reporting

Please select the one below that is the best fit for your research. If you are not sure, read the appropriate sections before making your selection.

☒ Life sciences ☐ Behavioural & social sciences ☐ Ecological, evolutionary & environmental sciences

For a reference copy of the document with all sections, see nature.com/documents/nr-reporting-summary-flat.pdf

Life sciences study design

All studies must disclose on these points even when the disclosure is negative.

Sample size	Three biological replicates for bulk RNA-seq, proteome and phospho proteome.
Data exclusions	No data exclusions.
Replication	Three biological replicates for bulk RNA-seq, proteome and phospho proteome.
Randomization	The cells and animals used for IUE are randomized transfected or injected with different plasmids. For the IUE-scRNA-Seq, the good IUE brains (n > 3) are used to generate the single cell data
Blinding	Blinding is not possible due to we need to label the IUE condition with scRNA-Seq experiments. But the scRNA-Seq annotation is blinding, as we integrate all condition and cluster all cells in one UMAP embedding. In this case, we do not annotate the specific condition in basis.

Reporting for specific materials, systems and methods

We require information from authors about some types of materials, experimental systems and methods used in many studies. Here, indicate whether each material, system or method listed is relevant to your study. If you are not sure if a list item applies to your research, read the appropriate section before selecting a response.

Materials & experimental systems

n/a	Involved in the study
☐	☒ Antibodies
☐	☒ Eukaryotic cell lines
☒	☐ Palaeontology and archaeology
☐	☒ Animals and other organisms
☒	☐ Clinical data
☒	☐ Dual use research of concern
☒	☐ Plants

Methods

n/a	Involved in the study
☐	☒ ChIP-seq
☒	☐ Flow cytometry
☒	☐ MRI-based neuroimaging

Antibodies

Antibodies used	anti-Tbr2 (1:200, Abcam, ab23345), anti-Ki67 (1:500, Thermo, 14-5698-82), anti-Satb2 (1:100, Abcam, ab92446), anti-NeuN (1:400, SYSY, 266 004), anti-Gad1 (1:200, SYSY, 198 308), anti-BrdU (1:400, Proteintech, 66241-1-Ig), anti-Nfib (1:100, Sigma, HPA003956), and anti-Hmgn3 (1:100, Abcam, ab72233)
Validation	IHC and CUT&RUN

Eukaryotic cell lines

Policy information about cell lines and Sex and Gender in Research

Cell line source(s)	IMR90 iPSC line from WiCell
Authentication	*Describe the authentication procedures for each cell line used OR declare that none of the cell lines used were authenticated.*
Mycoplasma contamination	Negative for mycoplasma.
Commonly misidentified lines (See ICLAC register)	No

Animals and other research organisms

Policy information about studies involving animals; ARRIVE guidelines recommended for reporting animal research, and Sex and Gender in Research

Laboratory animals	Plugged C57BL/6J females were either purchased from Janvier Labs or obtained from the core breeding unit at BML, University of Southern Denmark, and E0.5 was defined as the morningtimepoint on which the vaginal plug appeared. Mice either at E12.5 or E13.5 (n=3 embryos per condition per timepoints) were subjected to in utero electroporation and embryos harvested.
Wild animals	No wild animals used in this study.
Reporting on sex	For the IUE, we have to only use female. For the embryos, we have not done any PCR to validate the sex.
Field-collected samples	NA
Ethics oversight	Approval number: 2023-15-0201-01568

Note that full information on the approval of the study protocol must also be provided in the manuscript.

Plants

Seed stocks	NA
Novel plant genotypes	NA
Authentication	NA

ChIP-seq

Data deposition

☒ Confirm that both raw and final processed data have been deposited in a public database such as GEO.

☒ Confirm that you have deposited or provided access to graph files (e.g. BED files) for the called peaks.

Data access links<br>*May remain private before publication.*	All datasets generated in this study are publicly available. Bulk RNA sequencing data generated during this study have been deposited in the Gene Expression Omnibus (GEO) under accession number GSE327199.
Files in database submission	GSM9651173_phos_batch_igg_ctrl_R1.bigWig<br>GSM9651174_phos_batch_igg_ctrl_R2.bigWig<br>GSM9651175_Hmgn3_Mu_R1.bigWig<br>GSM9651176_Hmgn3_Mu_R2.bigWig<br>GSM9651177_Hmgn3_WT_R1.bigWig<br>GSM9651178_Hmgn3_WT_R2.bigWig<br>GSM9651179_NFIB_Mu_R1.bigWig<br>GSM9651180_NFIB_Mu_R2.bigWig<br>GSM9651181_NFIB_WT_R1.bigWig<br>GSM9651182_NFIB_WT_R2.bigWig
Genome browser session<br>(e.g. UCSC)	https://genome.ucsc.edu/s/Xuan1995/mm10

Methodology

Replicates	Biological replicates (n=2 per condition) were generated for all the CUT&RUN assays.
Sequencing depth	Samples were prepared following the EpiCypher CUTANA ChIP/CUT&RUN Kit protocol (Epicypher, SKU: 14-1048). An IgG antibody served as a negative control to evaluate non-specific background signal from chromatin captured during immunoprecipitation, and an E. Coli spike-in control was added for normalizing enrichment during analysis. CUT&RUN libraries were prepared according to the CUTANA™ CUT&RUN Library Prep Kit (Epicypher) for Illumina, and each sample was uniquely barcoded with the Multiplex Oligos for Illumina. Libraries were pooled and sequenced using 75 base pair paired-end reads on the NovaSeq 6000 platform, obtaining a sequencing depth of at least 10-12 million reads per sample.
Antibodies	CUT&RUN experiments were performed on E17.5 IUE sorted cells using specific antibodies for the following proteins: Flag (Sigma, M2 clone F1804), and normal IgG. Biological replicates (n=2 per condition) were generated for all the CUT&RUN assays.
Peak calling parameters	SEACR is used to call the peak with default parameter (https://github.com/FredHutch/SEACR).
Data quality	FDR 1% peaks are considered and used in following ChIPSeeker annotation.
Software	nf-core pipeline is used to process CUT&RUN data (https://nf-co.re/cutandrun/1.1/docs/usage/).
