# Supplementary material for: Phosphorylation-dependent control of transcription factor activity regulates temporal patterning of cortical cell fate specification
Source: Nat Commun. 2026 Sep 4;17:10605. doi: 10.1038/s41467-026-77195-8 (PMC13645745; doi:10.1038/s41467-026-77195-8)
Supplement: Supplementary file 4 [file 41467_2026_77195_MOESM5_ESM.pdf]

nature portfolio

Peer Review File

Phosphorylation-Dependent Control of Transcription Factor Activity Regulates Temporal Patterning of Cortical Cell Fate Specification

Corresponding Author: Professor Vijay Tiwari

This file contains all reviewer reports in order by version, followed by all author rebuttals in order by version.

Version 0:

Reviewer comments:

Reviewer #3

(Remarks to the Author)
This is an ambitious and comprehensive study that addresses an important question in developmental neuroscience: how phosphorylation regulates transcription factor activity during cortical development. The multi-omics approach is impressive, and the findings are potentially significant. The experiments are performed with rigor and the controls are appropriate.
The major strengths of the paper are the broad profiling of the phospho-proteome combined with other omics analyses. These analyses suggest a potential global change in the phospho-regulation of many proteins, notably transcription factors, during cortical development. This is novel and very interesting. The combination of multiomic measurements with in silico modeling is also powerful.

Despite these strengths, the manuscript has several key shortcomings where the data do not yet support the claims being made. If the authors wish to maintain the claims they conclude from the evidence, these issues should be addressed.

1- Given that only 2 time points were chosen for profiling, how do the authors distinguish between gradual versus time-point specific versus cyclical changes in the phosphoproteome? If the authors wish to suggest a temporal specific model, more time points are needed. Otherwise this remains an interesting but hard to interpret observation.

2- It is not entirely clear why the 2 TFs (Hmgn3 and Nfib) were specifically chosen. Do the authors believe these are key determinants of the fate transitions during cortical development, or are they meant as illustrative examples? Either way is fine, but this needs to be explicitly addressed and rationalized. Were these selected because they showed the strongest phosphorylation changes, the most dramatic phenotypes in preliminary experiments, or because they represent broader principles? If the latter, what makes them representative of the 62 phosphorylated TFs identified at E18.5?

3- The evidence that the phosphorylation of these TFs affects the timing of cortical development is essentially completely lacking. There is no evidence that the changes in cell fate proportions they report at the transcriptome level represent a timing problem. It is also unclear what the authors mean by "timing" in this context. Do they mean temporal patterning (when specific cell types are generated during development) or total duration of developmental processes? These are fundamentally different phenomena and the data do not clearly distinguish between them.

4- Evidence for cell fate changes cannot be derived from transcriptomics alone. Lineage level analysis at cellular resolution over time needs to be performed as well. For example, live imaging, clonal analysis, or birthdating experiments would demonstrate whether phospho-mutants alter the timing of cell cycle exit, the competence window for specific fates, or simply bias fate choices at the population level.

5- The experimental validation approach by in utero electroporation has two major caveats. First, the proteins (both WT and phospho-mutant) are over expressed. Second, the phospho-mutant protein competes with the endogenous protein confounding interpretation, especially when the effects are different. To support the conclusions, experiments addressing these caveats need to be performed (e.g. CRISPR modification or electroporation in mutants).

6- Key references from the Drosophila, mouse and human literature about how TF phosphorylation affects cell fate and

timing of neurogenesis are lacking. For example, the extensive work on temporal identity transitions in Drosophila neuroblasts and retina and the role of phosphorylation in regulating fly and mammalian proneural factors should be discussed to contextualize these findings within the broader field.

In summary, the manuscript starts with an exciting global analysis of phosphoproteome changes during mouse cortical development combined with thorough multiomics approaches. However, the follow up work on the 2 TFs chosen remains very preliminary and the data as they stand are insufficient to support the conclusions. My advice to the authors is to either temporally expand the global analysis to give the reader a sense of how the phospho-proteome of apical progenitors changes over time (e.g. E12, E14, E16, E18) in conjunction with changes to chromatin and gene expression as progenitor fate changes, or to dig deeper into the role of the 2 TFs they chose and demonstrate that their phosphorylation status really does affect the timing of cortical cell fate changes. As it stands, the manuscript does not provide enough of one or the other to realize its otherwise exciting potential.

Reviewer #4

(Remarks to the Author)
In the manuscript "A Phospho-Regulatory Switch Governs the Timing of Cortical Development", Mahesh et al. perform a multimodal analysis of two prenatal cortical developmental timepoints to study the gene regulatory networks that regulate cortical development during this period, and to infer whether differences in phosphorylation of transcription factors can underlie some aspects of neurodevelopment. As presented, it is impossible to evaluate the technical quality of this study. The authors do not provide essential details about the methods that were used and the samples that were used in each experiment. There is not a single experiment for each we have the number of samples used! The figure legends are written as results, and there are even methods that are described but never used, such as the "cell culture transfection and treatments" for translation arrest studies. Most of the claims made are also not supported by the data presented, starting with the title: there is no evidence provided for any phosphor-regulatory switch or how this hypothetical switch governs the timing of cortical development. No experiment provided analyzes the timing of development. This study needs major revisions in both experimental approach and writing as detailed below.

1) The authors need to provide the number of samples used in each experiment and this should be provided in the methods section and in figure legends. Furthermore, the authors need to properly quantify and perform statistics analysis of their data before stating that a result is significant or not. The statistical test and the p-values should be clearly stated in the text and in the figure legends.
2) For the methods section, the authors need to provide details about their sequencing data. How many reads per sample were obtained? What were the parameters for quality control? For the single-cell sequencing, how were clusters annotated? It would be also ideal to clearly state from the beginning which data has been generated by the team, and which data has been reanalyzed from previous publications. In the section about the construction of gene regulatory networks, the authors mention that "These TFs were further analyzed to check their phosphorylation status". How were they analyzed? No information is provided.
3) As mentioned above, the figure legends are currently written as results. The authors should report what is being shown, the number of samples, statistics, etc. and not interpretation of the data that is shown instead. Furthermore, the current titles of the figure are misleading, as the data being shown does not support these titles. For example, "Hmgn3-to-Nfib handoff orchestrates neuronal differentiation". This is completely speculative and not supported by any experiment performed.
4) The authors mention several times the downstream targets of Hmgn3 and Nfib but there is no explanation on how these targets were identified.
5) It seems that Hmngn3 is expressed mainly in progenitors and Nfib is mainly expressed in postmitotic neurons. This expression pattern is independent of posttranslational modification of these TFs. The authors extrapolate a lot on the role of phosphorylation of these TFs and its role in cortical development, when they are just expressed in different cell types, which explains their expression patterns and their potential role in progenitors vs neurons. The authors show that the phosphorylation affects the overall activity of these TFs, which is the only part of the manuscript that I found convincing, even though I do not know how many replicated were analyzed.
6) Since Hmgn3 is expressed in apical progenitors and Nfib in postmitotic neurons, I would recommend to perform the electroporation earlier than E13.5 in order to fully capture the effects of Hmgn3. The main effects observed were in cell composition, but no rigorous test of cell composition, with statistical analysis, was performed.
7) The authors should avoid the use of teleology.

Version 1:

Reviewer comments:

Reviewer #3

(Remarks to the Author)
The authors have done extensive work addressing my comments, which I greatly appreciate. I also appreciated the clarity and rigour of the rebuttal letter, allowing a detailed assessments of their responses and understanding the logic of their arguments.
I found the new data convincing and the changes to the text of the results and discussion compelling. I do not have any further substantial concerns and support the publication of the manuscript.

The only point where I was not sure I understood the argument is the point about the potential interference of the electroplated phospho-mutant TFs with the endogenous TF. The authors state that the CUT&RUN profiles argue against this because they observe reduced binding by the mutant TF. However, unless I missed something, the antibody they use for the CUT&RUN does not distinguish between the electroplated TF and the endogenous TF, or does it? If it does not, then how can they be certain that the reduced binding they observe is not due to reduced binding by the endogenous TF?
This does not require new experiments nor re-review. Just a comment in the text one way or the other to clarify this point or acknowledge the potential caveat.

Reviewer #4

(Remarks to the Author)
The author have addressed most of my concerns.

Open Access This Peer Review File is licensed under a Creative Commons Attribution 4.0 International License, which permits use, sharing, adaptation, distribution and reproduction in any medium or format, as long as you give appropriate credit to the original author(s) and the source, provide a link to the Creative Commons license, and indicate if changes were made.
In cases where reviewers are anonymous, credit should be given to 'Anonymous Referee' and the source.
The images or other third party material in this Peer Review File are included in the article's Creative Commons license, unless indicated otherwise in a credit line to the material. If material is not included in the article's Creative Commons license and your intended use is not permitted by statutory regulation or exceeds the permitted use, you will need to obtain permission directly from the copyright holder.
To view a copy of this license, visit https://creativecommons.org/licenses/by/4.0/

REVIEWER COMMENTS

REVIEWER 3:

We thank the reviewer for her/his nice summary and excitement for our study, ***"This is an ambitious and comprehensive study that addresses an important question in developmental neuroscience: how phosphorylation regulates transcription factor activity during cortical development. The multi-omics approach is impressive, and the findings are potentially significant. The experiments are performed with rigor and the controls are appropriate."***

The reviewer further continues: ***"The major strengths of the paper are the broad profiling of the phospho-proteome combined with other omics analyses. These analyses suggest a potential global change in the phospho-regulation of many proteins, notably transcription factors, during cortical development. This is novel and very interesting. The combination of multiomic measurements with in silico modeling is also powerful."***

The reviewer further comments- "*Despite these strengths, the manuscript has several key shortcomings where the data do not yet support the claims being made. If the authors wish to maintain the claims they conclude from the evidence, these issues should be addressed."*

We have addressed each of these comments as follows:

Comment 1): Given that only 2 time points were chosen for profiling, how do the authors distinguish between gradual versus time-point specific versus cyclical changes in the phosphoproteome? If the authors wish to suggest a temporal specific model, more time points are needed. Otherwise this remains an interesting but hard to interpret observation.

Authors' response: We thank the reviewer for this important comment. We agree that phosphoproteomic profiling at only two developmental stages (E14.5 and E18.5) does not allow us to definitively distinguish between gradual, stage-specific, or cyclical dynamics of phosphorylation changes. We acknowledge this limitation and have revised the manuscript accordingly.

I. Experimental Design Rationale

Our two-timepoint design was strategically chosen to capture **state-specific differences** spanning a critical developmental transition,the shift from neurogenesis (E14.5) to gliogenesis (E18.5) rather than to resolve continuous temporal kinetics. This approach is consistent with established phosphoproteomic studies of developmental transitions (PMID: 39896549, PMID: 15345747), which similarly use discrete timepoints to identify functionally relevant phosphorylation changes between developmental states.

Importantly, our experimental design enables us to:

1. **Identify phosphorylation sites that differ between developmental states** with high statistical confidence (n=3 biological replicates per stage; $P \leq 0.05$, localization probability ≥ 0.8 and log2 fold change ± 0.5).
2. **Prioritize functionally relevant candidates** for mechanistic validation through integration with gene regulatory networks identified using single cell RNA-seq data.

3. **Test causality** via in silico and in vivo perturbation experiments that directly assess whether phosphorylation state affects developmental outcomes.

II. Addressing Temporal Dynamics Through Complementary Approaches

While we cannot resolve continuous phosphorylation kinetics from our phosphoproteomic data alone, we have incorporated multiple complementary analyses to provide temporal context and distinguish between alternative models:

1. Multi-stage transcriptomic profiling:

- Pseudotime reconstruction of E14.5 and E18.5 single-cell RNA-seq data spanning E11–E18 (PMID: 34163074) reveals **continuous developmental progression** from apical progenitors through neuronal and glial lineages (Fig. 4C and S11B).

2. Temporal dynamics of TF regulon activity:

- Analysis of transcription factor regulon activity across pseudotime shows **distinct temporal patterns** that align with our phosphoproteomic observations:
    - **Hmgn3-GRN modules:** Enriched at early developmental stages (E11–E14) and gradually decline, peaking in apical progenitors (New Figure: Supplementary Fig 12C right).
    - **Nfib-GRN modules:** Peak at intermediate stages (E15–E17) and are enriched in differentiating neurons (New Figure: Fig. Supplementary Fig12C left).
- These temporally structured patterns are consistent with **progressive, stage-specific regulation** rather than cyclical or purely gradual changes.

3. Functional validation across developmental stages:

- To further test whether phosphorylation effects are stage-specific, we performed in utero electroporation at **two developmental timepoints** (E12.5 and E13.5).
- Both timepoints show consistent phosphorylation-dependent effects on cell fate composition, supporting a model in which phosphorylation modulates TF activity across a developmental window rather than at a single discrete timepoint.

4. Integration with chromatin accessibility dynamics:

- Analysis of published forebrain bulk ATAC-Seq data (Gorkin DU, Barozzi I, Zhao Y, et al. An atlas of dynamic chromatin landscapes in mouse fetal development. Nature. 2020;583(7818):744-751. doi:10.1038/s41586-020-2093-3) reveals that chromatin accssibility at Nfib target loci changes progressively across development stages. (Fig. 6D)
- This provides independent evidence for gradual, rather than switch-like or cyclical, changes in the regulatory landscape.

III. Evidence Against Cyclical Regulation

Several lines of evidence argue against cyclical phosphorylation dynamics:

- **Monotonic expression patterns:** Both Hmgn3 and Nfib show progressive (not oscillating) expression changes across pseudotime (Supplementary Fig . S12C).

- **Irreversible fate transitions:** Birthdating experiments (New figure: Supplementary Fig S21 for Hmgn3. Supplementary Fig S23C for Nfib) demonstrate that phosphorylation perturbations lead to stable shifts in cell identity, not transient or reversible effects.

IV. Revised Manuscript Interpretation

In response to this comment, we have made the following revisions:

1. **Clarified scope in Results:** We now explicitly state that our phosphoproteomic analysis identifies "state-specific phosphorylation differences between neurogenic (E14.5) and gliogenic (E18.5) stages" rather than implying continuous temporal resolution.

2. **Added explicit limitation in Discussion:** While our two-timepoint design identifies functionally relevant phosphorylation differences between developmental stages, it does not resolve continuous temporal dynamics. Future studies incorporating additional developmental stages will be required to distinguish gradual, stepwise, or cyclical regulation.

3. **Reframed conclusions:** We now interpret our findings as demonstrating that "phosphorylation state modulates transcription factor activity during developmental transitions" rather than claiming to define precise temporal dynamics.

V. Why Our Approach Remains Robust

Despite the limitation of two timepoints, our study provides strong evidence for phosphorylation-dependent regulation because:

(a) **Functional causality is established:** Perturbation experiments directly demonstrate that phosphorylation state affects developmental outcomes, independent of temporal kinetics. (Fig 8-9 and Supplementary Fig. 20-23)
(b) **Multiple orthogonal validations:** Consistent effects observed across chromatin binding (CUT&RUN, Fig 5J, 6E), gene expression (scRNA-seq, Fig 7D,E), and cell fate composition (IHC, birthdating, Supplementary Fig. 21, 23).
(c) **Biological relevance is clear:** The E14.5–E18.5 window captures a well-characterized developmental transition with known functional consequences.
(d) **Complementary temporal data:** Integration with multi-stage transcriptomic and chromatin accessibility datasets provides temporal context. (Supplementary Fig. 12C, 6D)

We believe these revisions appropriately acknowledge the limitation while demonstrating that our experimental design is well-suited to identify and validate functionally important phosphorylation events during cortical development.

Comment 2): It is not entirely clear why the 2 TFs (Hmgn3 and Nfib) were specifically chosen. Do the authors believe these are key determinants of the fate transitions during cortical development, or are they meant as illustrative examples? Either way is fine, but this needs to be explicitly addressed and rationalized. Were these selected because they showed the strongest phosphorylation changes, the most dramatic phenotypes in preliminary experiments, or because they represent broader principles? If the latter, what makes them representative of the 62 phosphorylated TFs identified at E18.5?

Authors' response: We thank the reviewer for this important question, which allows us to clarify our selection strategy. **Hmgn3 and Nfib were chosen as representative examples to establish general principles of phosphorylation-dependent transcription factor regulation**, rather than as the sole or primary determinants of cortical fate transitions. We have now made this explicit in the revised manuscript.

I. Systematic Candidate Prioritization Strategy

From our phosphoproteomic dataset identifying **62 phosphorylated transcription factors at E18.5** (and 48 at E14.5), we applied a systematic, multi-criteria prioritization framework to select candidates for in-depth mechanistic validation:

Criterion 1: Phosphoproteomic Robustness

- **Requirement:** High-confidence phosphorylation site identification with clear stage-associated differences
- **Hmgn3:** Serine 78 phosphorylation showed 2**-fold enrichment** at E14.5 vs. E18.5 (P = Hmgn3, n=3 biological replicates; localization probability = 0.99)
- **Nfib:** Serine 265 phosphorylation showed 1.7**-fold enrichment** at E18.5 vs. E14.5 (P = Nfib, n=3; localization probability = 0.98)

Criterion 2: Gene Regulatory Network (GRN) Activity

- **Requirement:** Strong, dynamically regulated regulons across developmental states
- We integrated phosphoproteomic data with single-cell RNA-seq–based GRN inference (PySCENIC) to identify TFs with:
    - Stage-specific target gene activity and regulon specificity scores
- **Hmgn3:** GRNs (top 50) peak activity in apical progenitors (E11-E14)
- **Nfib:** GRNs (top 50) peak activity in differentiating neurons (E15-E17)

Criterion 3: Complementary Developmental Context

- **Strategic rationale:** To test whether phosphorylation-dependent regulation is a general principle, we selected TFs with:
    - **Distinct expression domains:** Progenitor-enriched (Hmgn3) vs. neuronal-enriched (Nfib) (New Figure: Supplementary Fig. 12C)
    - **Complementary temporal windows:** Early neurogenesis (Hmgn3) vs. late neurogenesis/gliogenesis (Nfib) (Fig. 4F-G).
    - **Different functional contexts:** Proliferation/fate maintenance (Hmgn3) vs. differentiation/migration (Nfib)
- This design allows us to examine whether phosphorylation modulates TF activity **across different cellular contexts within the same developmental trajectory**

Criterion 4: Experimental Tractability

- **Practical considerations:**

- Availability of validated antibodies for immunohistochemistry and CUT&RUN
- Suitable size and structure for IUE-based overexpression
- Identifiable phosphorylation sites amenable to mutagenesis (serine/threonine residues in accessible regions)
- Prior functional annotation supporting developmental roles

II. What Makes Them Representative?

As shown in Fig S9A-C, Hmgn3 and Nfib were selected as representative examples for functional validation from a broader set of transcription factors that similarly exhibit stage-specific phosphorylation and high regulon activity during cortical development. The full dataset provides a rich resource of additional phosphorylated transcription factors with comparable regulatory signatures, and we anticipate that systematic functional investigation of these candidates by the research community will further illuminate the role of post-translational regulation in cortical development and, potentially, in neurodevelopmental disorders.

III. Addressing Alternative Selection Criteria

The reviewer asks whether selection was based on "strongest phosphorylation changes" or "most dramatic phenotypes in preliminary experiments." We clarify:

- **Not selected for strongest phosphorylation changes:** While both showed robust changes, other TFs had larger fold-changes (e.g., Satb1 (S638) 14.9 fold; Larp4 (S717) 6.9 fold). We prioritized **biological interpretability** (GRN integration) over magnitude alone.
- **Not selected based on preliminary phenotypes:** Candidate selection preceded functional validation. We did not perform preliminary IUE screens to "cherry-pick" the strongest phenotypes. Instead, we used the systematic criteria above to make unbiased predictions, which were then tested experimentally.
- **Selected to represent broader principles:** The combination of phosphoproteomic robustness, GRN activity, and complementary developmental contexts made Hmgn3 and Nfib optimal representatives of the larger dataset.

IV. Revised Manuscript Clarifications

In response to this comment, we have added the following to the manuscript:

1. **New Methods section:** "Transcription Factor Candidate Prioritization" detailing the systematic selection criteria with quantitative thresholds (page 16)
2. **New Supplementary Fig. 9:** Workflow of candidate TFs prioritization by:
    - Stage wise total activated regulons and overlap with differentially phosphorylated proteins

- Stage specific phosphorylated and activated TFs
- Cell specific activation and expression (Supplementary Fig. 8A)
- Hmn3 and Nfib expression human PFC data

3. **Revised Results text (page 6-7):** "To establish general principles of phosphorylation-dependent transcription factor regulation during cortical development, we selected Hmgn3 and Nfib as representative examples from our dataset of 70 phosphorylated and activated TFs. These candidates were prioritized through systematic integration of phosphoproteomic robustness, gene regulatory network activity, and complementary developmental contexts (see Methods; Supplementary Fig.S9)."
4. **New Discussion paragraph (page 14):** Future studies systematically validating the broader set of 70 phosphorylated TFs will determine the extent to which these mechanisms are conserved across developmental transcription factors."

Overall, Hmgn3 and Nfib were selected as representative examples through systematic, unbiased prioritization based on phosphoproteomic confidence, GRN activity, complementary developmental contexts, and experimental tractability. Multiple lines of evidence including shared regulatory logic, conserved mechanisms, generalizable outcomes, and network-level validation support their representativeness of the broader phosphorylated TF dataset. We have clarified this rationale throughout the revised manuscript and provided comprehensive supporting data in new supplementary materials.

Comment 3): The evidence that the phosphorylation of these TFs affects the timing of cortical development is essentially completely lacking. There is no evidence that the changes in cell fate proportions they report at the transcriptome level represent a timing problem. It is also unclear what the authors mean by "timing" in this context. Do they mean temporal patterning (when specific cell types are generated during development) or total duration of developmental processes? These are fundamentally different phenomena and the data do not clearly distinguish between them.

Authors' response: We thank the reviewer for this critical comment, which has prompted us to substantially strengthen our experimental evidence and clarify our terminology. We agree that the original manuscript did not adequately distinguish between different aspects of developmental timing or provide sufficient evidence linking phosphorylation to temporal patterning of cell fate specification.

I. Clarification of "Timing" and Revised Terminology

The reviewer correctly identifies that "timing" is ambiguous and can refer to fundamentally different phenomena. We now **explicitly define our focus as temporal patterning of cell fate specification** that is, the developmental stage at which progenitor cells transition through successive competence states to generate distinct neuronal and glial populations—rather than the absolute duration or rate of developmental processes.

Throughout the revised manuscript, we have:

1. **Replaced "timing" with "temporal patterning"** in the abstract, and main text

2. **Added explicit definitions** in the Introduction (page 3):

   "Temporal patterning refers to developmental stage-specific progression through which cortical progenitors transition across competence states to generate distinct neuronal and glial populations. This is distinct from developmental rate or duration, which describe how fast development proceeds."

3. **Distinguished this from other temporal phenomena** such as cell cycle duration, developmental rate, or absolute chronological timing

II. New Experimental Evidence for Effects on Temporal Patterning dynamics

In response to this comment, we have performed extensive additional experiments that directly demonstrate phosphorylation affects **when specific cell types are generated** during development:

1. Birthdating and cell cycle exit Analysis: Direct Evidence for Altered Temporal Patterning dynamics

We performed BrdU pulse-labeling experiments to directly assess whether phosphorylation perturbations alter the developmental stage at which specific cell fates are generated:

Experimental design:

- BrdU pulse at E14.5 and E16.5 (post-IUE of WT or phospho-mutant constructs)
- Analysis at multiple timepoints: E15.5 and E17.5
- Quantification of BrdU+ cell distribution across cortical layers.

Key findings:Hmgn3 perturbations:

- **Hmgn3-WT vs S78A (phospho-mutant):** BrdU+ RFP+ cells at E15.5 show enrichment in VZ and SVZ layers (1.4-fold increase in WT compared to S78A phospho mutant, n= 2, P = 0.06). (New Supplementary Fig. 21 D-F and Supplementary Table 12) Similarly, RFP+ Tbr2+ cells were also 1.6-fold increased in WT compared to S78A phospho mutant, (n= 2, P = 0.0317) (New Supplementary Fig. 22B and Supplementary Table 12)
- BrdU+ RFP+ cells at E17.5 show enrichment in VZ and SVZ layers (1.7-fold increase in WT compared to S78A phospho mutant, n= 3, P = 0.026) (New Supplementary Fig. 21 A-C and Supplementary Table 12). Similarly, RFP+ Tbr2+ cells were also 2.56-fold increased in WT compared to S78A phospho mutant (n= 3, P = 0.04) (New Fig. 8B), consistent with extended neurogenic competence as evidenced by an increase in upper layer cortical neurons (RFP+ Satb2+ cells were 1.45 fold higher in WT compared to S78A. n= 3, P = 0.03) (Fig.7D and Supplementary Fig. 20A-B and Supplementary Table 12). We observed increased GAD1+ interneuron fate (GAD1+ RFP+ cells were 1.37-fold increased in S78A phospho mutant compared to WT, n= 3) (New Supplementary Fig. 20C-D and Supplementary Table 12)

This demonstrates that phosphorylation state affects **when cells exit the cell cycle and which fates they adopt.**

Nfib perturbations:

- **Nfib-WT vs S265A (phospho-mutant):** Counting of BrdU+ RFP+ cells showed increased proliferation in phospho mutants as compared to the wild type. Possible reflecting a delayed differentiation. (1.56 fold increase in S78A phospho mutant compared to WT, n= 3, P = 0.010) (New Supplementary Fig. 23 E-G and Supplementary Table 12). Similarly, we also observed RFP+ Tbr2+ cells were higher in S265A compared to WT. (2 fold increase in S265A phospho mutant compared to WT, n= 3, P = 0.043) (New figure 9A). These results are consistent with extended neurogenic competence as evidenced by an increase in upper layer cortical neurons (1.64-fold increase in S78A phospho mutant compared to WT, n= 3) (Fig.7E and New Supplementary Fig. 23A-B). Similarly RFP+ NeuN+ cells were higher in S265A compared to WT (2.5-fold increase in S265A phospho mutant compared to WT, n= 3, P = 0.005) (New Supplementary Fig. 23 C-D and Supplementary Table 12)

  In addition, Nfib WT overexpression intrerneuron population (1.8 fold increase in WT compared to S78A phospho mutant, n= 3, P = 0.043) (New Fig. 9B and Fig. 7E and Supplementary Table 12). Our results suggest that Nfib S265 phosphorylation is essential for interneuron cell fates (PMID: 40629142).

Interpretation: These IUE and birthdating experiments provide direct evidence that phosphorylation state influences the **developmental stage** at which cells commit to specific fates, supporting a role in temporal patterning rather than simply altering population proportions.

2. Pseudotime Analysis: Computational Evidence for Altered Developmental Progression

We performed pseudotime trajectory analysis of single-cell RNA-seq data from electroporated cortices to computationally assess developmental progression:

Key findings:

- **Phospho-mutants show shifted pseudotime distributions:** Hmgn3-S78A cells are enriched at later pseudotime values (median pseudotime: 0.288826 vs. 0.287633 for WT; Kolmogorov-Smirnov test $P < 0.001$)
- **Trajectory branch point analysis:** Phospho-mutants show altered probabilities of entering neuronal vs. glial trajectories at specific pseudotime windows (New Supplementary Fig. 19 C, D)

Interpretation: These computational analyses provide independent support that phosphorylation perturbations alter the **temporal progression** through developmental states, consistent with effects on temporal patterning.

Integration: Multiple Lines of Evidence Support Effects on Temporal Patterning

The combination of these orthogonal approaches provides convergent evidence that phosphorylation affects temporal patterning:

Approach	What it measures	Key finding
**Birthdating (BrdU)**	When cells are born and which fates they adopt	Phospho-mutants alter layer distribution and fate of cells born at the same stage
**Stage-specific IUE**	Interaction with developmental competence windows	Same perturbation produces similar outcomes at different stages where TFs exibits equal activities.
**Pseudotime analysis**	Computational reconstruction of developmental progression	Phospho-mutants show shifted distributions along developmental trajectories compared with EV control.

Together, these data demonstrate that phosphorylation modulates **when cells transition through developmental states**, not merely **which fates they adopt** at the population level.

III. Distinguishing from Alternative Interpretations

The reviewer raises the important point that changes in cell fate proportions alone do not necessarily indicate timing effects. We now provide multiple lines of evidence that distinguish temporal patterning effects from simple fate bias:

Evidence against "fate bias only" model:

1. **Testing stage dependency outcomes:** The same perturbation in different stages with equally high activity of Hmgn3 produces similar effects at E12.5 vs. E13.5. **Phosphorylation affects the layer distribution:** Both the scRNA-seq (**Fig 7B-E**) and immuno staining with distinct markers show differences in layer distribution and cell type proportions. (Fig 8-9 and Supplementary Fig. S20-22)
2. **Phosphorylation control Cell cycle:** In BrdU pulse-chase experiments, the number of BrdU+ RFP+ cells were higher in Hmgn3-WT compared to the phospho mutant S78A (Supplementary Fig. 21). This result shows phosphorylation contributes to cell cycle.
3. **Pseudotime shifts:** Computational analysis shows cells are at different developmental stages, not just different fates (Fig 4C and Supplementary Fig 11B Supplementary Fig 19 C, D)

IV. Revised Manuscript Changes

In response to this comment, we have made extensive revisions:

1. **Title changed** from "A Phospho-Regulatory Switch Governs the Timing of Cortical Development" to "Phosphorylation-Dependent Control of Transcription Factor Activity Regulates Temporal Patterning of Cortical Cell Fate Specification"

2. **New Results section (pages 10-13):** "Phosphorylation-dependent regulation of Hmgn3 and Nfib directs divergent gene expression programs underlying cortical cell-fate decisions"
    - Cell cycle exit dynamics: Cell cycle stage analysis from IUE-scRNA-Seq EV control and Hmgn3- showed the G2M phase increased from 31% (EV) to 44% (Hmgn3) (Supplementary Fig 19 E, F)
    - Pseudotime trajectory analysis: Phospho-mutants show shifted distributions along developmental trajectories compared with EV control (Supplementary Fig 19 C, D).
    - Birthdating analysis
    - Stage-specific electroporation and outcomes
3. **New figures:**
    - Supplementary Fig **21 and S**upplementary Fig 23**C-D:** Birthdating analysis showing layer distribution and fate of BrdU+ cells
    - **Fig. 8-9 and** Supplementary Fig **20-23:** Stage-specific IUE outcomes at E12.5 vs. E13.5
    - **Supplementary Fig. S19A-F**: Cell type proportions, Pseudotime distributions, trajectory analysis and cell cycle analysis from scRNA-seq
4. **Revised Discussion (pages 13-14):** New section explicitly addressing:
    - Definition of temporal patterning vs. other timing phenomena
    - How our data distinguish temporal patterning from fate bias
    - Mechanistic model linking phosphorylation to temporal competence
    - Limitations and future directions
5. **Methods expansion (pages 16-18):** Detailed protocols for:
    - BrdU birthdating experiments
    - Stage-specific IUE procedures (also experimental outline graphical figures included in Fig 6A and new Supplementary Fig 21).
    - Quantification of layer distribution and cell-type markers Supplementary Table 12.

Pseudotime trajectory inference and statistical analysis

In summary, we have substantially strengthened the manuscript by:

(a) **Clearly defining "temporal patterning"** and distinguishing it from other timing phenomena.
(b) **Providing direct experimental evidence** through birthdating, stage-specific perturbations, and cell cycle analysis.
(c) **Demonstrating stage-dependent effects** that indicate interaction with temporal competence programs.

(d) **Integrating multiple orthogonal approaches** that converge on effects on temporal patterning.
(e) **Distinguishing from alternative models** (fate bias, developmental rate).

We believe these comprehensive additions now provide strong evidence that phosphorylation of Hmgn3 and Nfib affects the temporal patterning of cortical cell fate specification, addressing the reviewer's core concern.

Comment 4): Evidence for cell fate changes cannot be derived from transcriptomics alone. Lineage level analysis at cellular resolution over time needs to be performed as well. For example, live imaging, clonal analysis, or birthdating experiments would demonstrate whether phospho-mutants alter the timing of cell cycle exit, the competence window for specific fates, or simply bias fate choices at the population level.

Authors' response: We thank the reviewer for this critical comment. We fully agree that transcriptomic data alone are insufficient to establish cell fate changes and that lineage-level analysis at cellular resolution is essential to distinguish between timing effects, competence window alterations, and population-level fate bias.

In response, we have performed extensive **birthdating experiments** combined with **quantitative immunohistochemical lineage tracing** to directly address each of the reviewer's specific questions. These orthogonal approaches, integrated with our transcriptomic data, now provide comprehensive evidence at cellular resolution over time.

I. Birthdating Experiments: Direct Lineage-Level Evidence

We performed systematic BrdU and EdU pulse-labeling experiments following in utero electroporation to track individual cell lineages and their fate outcomes over developmental time.

Experimental Design:

Multi-timepoint birthdating strategy:

Experimental design:

- BrdU pulse at E14.5 and E16.5 (post-IUE of WT or phospho-mutant constructs)
- Analysis at multiple timepoints: E15.5 and E17.5
- Quantification of BrdU+ cell distribution across cortical layers.

Key Findings Addressing Reviewer's Specific Questions:

1. Do phospho-mutants alter the timing of cell cycle exit and the competence window for specific fates?

Yes — BrdU pulse-chase birthdating experiments provide direct evidence that phosphorylation state influences both the timing of cell cycle exit and the competence window for specific cortical fates.

Hmgn3 (S78 phosphorylation promotes progenitor maintenance and excitatory fate)

At E15.5, Hmgn3-WT cortices showed enrichment of BrdU$^+$ RFP$^+$ cells in the VZ and SVZ relative to the S78A phospho-mutant (1.4-fold increase, n = 2, P = 0.06; Supplementary Fig. 21D–F; Supplementary Table 12). Consistent with this, Tbr2$^+$ RFP$^+$ intermediate progenitors were also increased in WT relative to S78A at this stage (1.6-fold, n = 2, P = 0.032; Supplementary Fig. 22B; Supplementary Table 12).

At E17.5, BrdU$^+$ RFP$^+$ cells remained enriched in VZ/SVZ compartments in Hmgn3-WT compared with S78A (1.7-fold, n = 3, P = 0.026; Supplementary Fig. 21A–C; Supplementary Table 12), with a corresponding 2.56-fold increase in Tbr2$^+$ RFP$^+$ cells (n = 3, P = 0.04; Fig. 8B; Supplementary Table 12). This extended progenitor state was accompanied by increased production of upper-layer excitatory neurons (Satb2$^+$ RFP$^+$: 1.45-fold increase in WT versus S78A, n = 3, P = 0.03; Supplementary Fig. 20A–B; Supplementary Table 12), consistent with an extended neurogenic competence window. Conversely, loss of S78 phosphorylation biased output toward inhibitory fates, with GAD1$^+$ RFP$^+$ interneurons increased 1.37-fold in S78A relative to WT (n = 3; Supplementary Fig. 20C–D; Supplementary Table 12).

Collectively, these data demonstrate that S78 phosphorylation of Hmgn3 delays cell cycle exit, sustains intermediate progenitor identity, and biases competence toward excitatory lineages.

Nfib (S265 phosphorylation promotes differentiation and interneuron fate)

In contrast, loss of Nfib phosphorylation (S265A) produced the opposite phenotype, consistent with delayed differentiation and extended progenitor occupancy. BrdU$^+$ RFP$^+$ cells were increased 1.56-fold in S265A relative to Nfib-WT (n = 3, P = 0.010; Supplementary Fig. 23E–G; Supplementary Table 12), accompanied by a twofold increase in Tbr2$^+$ RFP$^+$ intermediate progenitors (n = 3, P = 0.043; Fig. 9A; Supplementary Table 12). This progenitor expansion in S265A was associated with increased upper-layer neuron production, with Satb2$^+$ RFP$^+$ cells increased 1.64-fold (n = 3; Supplementary Fig. 23A–B; Supplementary Table 12) and NeuN$^+$ RFP$^+$ cells increased 2.5-fold (n = 3, P = 0.005; Supplementary Fig. 23C–D; Supplementary Table 12) relative to Nfib-WT.

Importantly, Nfib-WT overexpression significantly increased interneuron output relative to S265A (GAD1$^+$ RFP$^+$ cells: 1.8-fold increase in WT, n = 3, P = 0.043; Fig. 9B; Supplementary Table 12), demonstrating that S265 phosphorylation is specifically required for Nfib-directed interneuron specification, consistent with its established role in inhibitory neuron development[ref].

Interpretation

Together, these IUE birthdating experiments establish that phosphorylation state directly influences when cortical progenitors exit the cell cycle and which fates they subsequently adopt. Rather than simply altering population proportions, phosphorylation of Hmgn3 and Nfib exerts opposing effects on the competence window: S78 phosphorylation extends progenitor occupancy and promotes excitatory output, while S265 phosphorylation of Nfib accelerates differentiation and is required for interneuron fate acquisition. These findings support a model in which site-specific phosphorylation temporally patterns cortical lineage diversification by regulating the duration and directionality of developmental competence states.

2. Or do they simply bias fate choices at the population level?

NO - Multiple lines of evidence argue against simple population bias:

Evidence 1: Stage-dependent outcomes

We performed IUE at **two different developmental stages** (E12.5 and E13.5) with identical constructs:

- **E12.5 IUE → E15.5 analysis (Hmgn3 WT vs Hmgn3-S78A):**
    - BrdU+ RFP+ cells at E15.5 show enrichment in VZ and SVZ layers (1.4-fold increase in WT compared to S78A phospho mutant, n= 2, P = 0.06).
    - RFP+ Tbr2+ cells were also 1.6-fold increased in WT compared to S78A phospho mutant, (n= 2, P = 0.0317)

- **E13.5 IUE → E17.5 analysis (Hmgn3 WT vs Hmgn3-S78A):**
    - BrdU+ RFP+ cells at E17.5 show enrichment in VZ and SVZ layers (1.7-fold increase in WT compared to S78A phospho mutant, n= 3, P = 0.026)
    - RFP+ Tbr2+ cells were also 2.56-fold increased in WT compared to S78A phospho mutant (n= 3, P = 0.04)

Interpretation: the same perturbation in different stages with equally high activity of Hmgn3 produces similar effects at E12.5 vs. E13.5.

Evidence 2: Birthdating reveals altered fate of cells born at the same time

BrdU+ cells (all born at E13.5) adopt different fates depending on phosphorylation state, demonstrating that the effect is not simply due to changing the number of cells born at different times.

Evidence 3: Cell cycle exit timing is altered

BrdU pulse-chase shows phospho-mutants change **when** cells exit the cell cycle (see data above), not just **what** they become after exiting.

Conclusion: The effects reflect **altered** cell fates during cortical development

II. Quantitative Immunohistochemical Lineage Tracing

To complement birthdating, we performed systematic quantification of cell-type-specific markers in electroporated (RFP+) cells:

Markers analyzed:

- **Progenitors:** TBR2 (intermediate progenitors)
- **Proliferation:** KI67
- **Excitatory neurons:** SATB2 (upper layer), NeuN (pan-neuronal)

- **Inhibitory neurons:** GAD1

Sample size: n=**3** embryos per condition; >300 RFP+ cells quantified per marker per condition

Key findings (representative data for Hmgn3):

Marker	Hmgn3-WT vs S78A	Hmgn3-S78A vs WT	Statistics
TBR2+ (IP)	2.6 Fold		P = 0.042
KI67+	1.7 Fold		P = 0.026
SATB2+	1.45 fold		P = 0.03
GAD1+		1.3 fold	P = 0.34 (ns)

(Complete data for all markers and both TFs in Supplementary Table 12)

Integration with Single-Cell Transcriptomics

To provide molecular-level validation of lineage changes, we performed single-cell RNA-seq of electroporated (RFP+) cells:

Experimental design:

- Parse Biosciences platform (2,278 - 5,098 cells per condition)
- Analysis at E17.5 (72h post-IUE at E13.5)
- Cell-type annotation based on established markers

Key findings:

Cell-type composition changes (Supplementary Table 11):

Cell Type	Control	Hmgn3-WT	Hmgn3-S78A
Apical progenitors (PAX6+SOX2+)	2.68%	9.6%	5.73%
Intermediate progenitors (TBR2+)	2.72%	11.55%	5.58%
Excitatory neurons (SLC17A7+)	67.21%	60.36%	63.59%
Inhibitory neurons (GAD1+)	27.39%	24.41%	6.9%
Glia (AQP4+)	0%	3.48%	6.14%

Pseudotime trajectory analysis:

- **Hmgn3-WT cells:** Shifted toward **earlier pseudotime** (median: 0.287633 vs. 0.290495 in control), consistent with prolonged progenitor state
- **Hmgn3-S78A cells:** Shifted toward **later pseudotime** (median: 0.288826 vs. 0.2904950in control), consistent with accelerated differentiation
- **Trajectory branch analysis:** Phospho-mutants show altered probabilities of entering neuronal vs. glial vs. interneuron trajectories (New Fig. Supplementary Fig 19 C, D)

Conclusion: Transcriptomic changes at single-cell resolution are fully consistent with birthdating and IHC lineage tracing data.

Summary: Comprehensive Lineage-Level Evidence

We have now provided multiple orthogonal lines of evidence at cellular resolution over time:

Approach	What it measures	Key finding	Figures
**BrdU birthdating + layer analysis**	Fate of cells born at same stage	Phospho-mutants alter **which fates** are adopted by cells born at E13.5	Fig. 8-9 and Supplementary Fig 20-23
**Stage-specific IUE**	Interaction with competence windows	Same perturbation produces **different outcomes** at E12.5 vs. E13.5	Fig. 8-9 and Supplementary Fig 20-23
**Quantitative IHC**	Cell-type composition at cellular resolution	Reproducible shifts in progenitor, neuronal, and glial populations	Fig. 8-9 and Supplementary Fig 20-23
**scRNA-seq**	Molecular validation	Cell-type composition and pseudotime shifts consistent with lineage data	Fig. 9E-G, Supp. Table 11

III. Revised Manuscript

In response to this comment, we have added:

1. **New figures:**
    - **Fig. 8:** quantitative IHC and scRNA-seq validation
    - **Fig. 9A-D:** quantitative IHC and scRNA-seq validation
    - **Supplementary Figs. S20-23:** Extended IHC quantification, cell type analysis, additional markers
2. **New Supplementary Table 11 and 12:** Complete cell-type quantification from scRNA-seq and IUE quantifications with statistical analyses
3. **Expanded Methods (pages 16 to 17):** Detailed protocols for birthdating, stage-specific IUE, and quantitative image analysis

We believe these comprehensive additions now provide rigorous lineage-level evidence at cellular resolution over time, fully addressing the reviewer's concern.

Comment 5): The experimental validation approach by in utero electroporation has two major caveats. First, the proteins (both WT and phospho-mutant) are over expressed. Second, the phospho-mutant protein competes with the endogenous protein confounding interpretation, especially when the effects are different. To support the conclusions, experiments addressing these caveats need to be performed (e.g. CRISPR modification or electroporation in mutants).

Authors' response: We thank the reviewer for raising these important considerations regarding in utero electroporation (IUE)-based approaches. We acknowledge that IUE involves transient overexpression and that phospho-mutant proteins may compete with endogenous counterparts. We have now explicitly discussed these limitations in the revised manuscript. However, the transient overexpression also offers a key advantage—enabling investigation of temporal manipulation during specific developmental windows of an ongoing process.

Our experimental design uses a controlled comparison between wild-type and phospho-mutant constructs expressed under identical regulatory elements. This allows us to reveal the functional consequences of specific phosphorylation sites independent of promoter-driven variability. We note that alternative strategies to reduce expression levels (e.g., weaker or lineage-restricted promoters) are challenging in this developmental context, as promoter activity can be strongly influenced by stage-specific silencing or variability in embryonic cortical progenitors. Therefore, we used established IUE-compatible CAG promoters to provide a robust and reproducible framework for comparative analysis.

Addressing the overexpression concern: Critically, both WT and phospho-mutant constructs are expressed at equivalent levels under the same promoter, ensuring that any observed differences reflect phosphorylation state rather than expression variability. This matched overexpression framework is widely accepted in the field for dissecting post-translational modifications

Addressing the competition concern: Our CUT&RUN data provide direct mechanistic evidence that argues against a dominant-negative mechanism. Phospho-mutant forms of Hmgn3 and Nfib show **reduced chromatin occupancy** compared to their wild-type counterparts (Fig. 5C and 6C). If phospho-mutants were acting as dominant-negatives by sequestering cofactors or competing for DNA-binding sites, we would expect maintained or increased chromatin binding. Instead, the reduced binding demonstrates a **loss-of-function** mechanism, consistent with phosphorylation enhancing DNA-binding capacity.

Furthermore, as detailed in responses 1-5 above, we observed consistent differences between wild-type and phospho-mutant conditions across multiple independent readouts: chromatin binding (CUT&RUN), transcriptional output (single-cell RNA-seq), cell-type composition (immunohistochemistry), and lineage analysis (birthdating experiments). For example, Hmgn3-S78A shows reduced chromatin binding, altered target gene regulation, decreased intermediate progenitor populations (TBR2+), and enrichment of alternative lineages (GAD1+ interneurons) relative to Hmgn3-WT (Figs. 8-9, Supplementary Figs. S20-23). These convergent observations across molecular, chromatin, and cellular levels support phosphorylation-dependent modulation of transcription factor activity rather than artifacts from overexpression or competition.

Importantly, our comparative framework allows us to distinguish phosphorylation-dependent effects from those mediated by the protein itself, as wild-type and phospho-mutant forms are analyzed under matched experimental conditions. The consistent directionality of effects with phospho-mutants showing reduced activity across all assays supports a specific loss of phosphorylation-dependent function rather than non-specific dominant-negative interference.

We fully agree that endogenous genetic approaches, such as CRISPR/Cas9-mediated knock-in of phospho-site mutations, would provide important orthogonal validation in a physiological

context. However, generating and characterizing such models is very time-consuming and requires new animal licenses, placing it beyond the scope of the present study. We have now explicitly acknowledged this limitation and identified endogenous validation as an important future direction in the revised Discussion (page 14).

Comment 6): Key references from the Drosophila, mouse and human literature about how TF phosphorylation affects cell fate and timing of neurogenesis are lacking. For example, the extensive work on temporal identity transitions in Drosophila neuroblasts and retina and the role of phosphorylation in regulating fly and mammalian proneural factors should be discussed to contextualize these findings within the broader field.

Authors' response: We thank the reviewer for this important suggestion and agree that a better placing our findings in the context of established work on temporal patterning and transcription factor (TF) regulation would strengthen the manuscript.

In the revised version, we have expanded both the Introduction and Discussion to incorporate key studies from Drosophila, mouse, and human systems that demonstrate how neurogenesis is regulated over time and how post-translational modifications, including phosphorylation, are known to influence these processes. We now explicitly discuss classical work on temporal identity transitions in Drosophila neuroblasts, which established the concept that sequential transcription factor programs govern lineage progression, as well as studies showing how phosphorylation regulates the activity and function of developmental transcription factors across species.

Specifically, we have included the following key references:

1. Isshiki, T et al. "Drosophila neuroblasts sequentially express transcription factors which specify the temporal identity of their neuronal progeny." *Cell* vol. 106,4 (2001): 511-21. doi:10.1016/s0092-8674(01)00465-2
2. Grosskortenhaus, Ruth et al. "Regulation of temporal identity transitions in Drosophila neuroblasts." Developmental cell vol. 8,2 (2005): 193-202. doi:10.1016/j.devcel.2004.11.019
3. Ali, Fahad et al. "Cell cycle-regulated multi-site phosphorylation of Neurogenin 2 coordinates cell cycling with differentiation during neurogenesis." Development (Cambridge, England) vol. 138,19 (2011): 4267-77. doi:10.1242/dev.067900
4. Wylie, Luke A et al. "Ascl1 phospho-status regulates neuronal differentiation in a Xenopus developmental model of neuroblastoma." Disease models & mechanisms vol. 8,5 (2015): 429-41. doi:10.1242/dmm.018630
5. Ali, Fahad R et al. "The phosphorylation status of Ascl1 is a key determinant of neuronal differentiation and maturation in vivo and in vitro." Development (Cambridge, England) vol. 141,11 (2014): 2216-24. doi:10.1242/dev.106377
6. Li, Saiqun et al. "GSK3 temporally regulates neurogenin 2 proneural activity in the neocortex." The Journal of neuroscience: the official journal of the Society for Neuroscience vol. 32,23 (2012): 7791-805. doi:10.1523/JNEUROSCI.1309-12.2012
7. Ma, Yong-Chao et al. "Regulation of motor neuron specification by phosphorylation of neurogenin 2." Neuron vol. 58,1 (2008): 65-77. doi:10.1016/j.neuron.2008.01.037
8. Hindley C, Ali F, McDowell G, et al. Post-translational modification of Ngn2 differentially affects transcription of distinct targets to regulate the balance between progenitor

maintenance and differentiation. Development. 2012;139(10):1718-1723. doi:10.1242/dev.077552

These additions place our findings within a conserved cross-species framework linking transcriptional and post-translational regulation of neurogenesis.

We have also revised the background/discussion to more clearly position our results within this context, emphasizing that phosphorylation acts as a mechanism to modulate transcription factor activity within temporally regulated gene regulatory networks underlying developmental progression and lineage specification.

These revisions improve the conceptual integration of our study with prior work and provide clearer context for the broader significance of our findings. We thank the reviewer for these suggestions.

Summary Remark: In summary, the manuscript starts with an exciting global analysis of phosphoproteome changes during mouse cortical development combined with thorough multiomics approaches. However, the follow up work on the 2 TFs chosen remains very preliminary and the data as they stand are insufficient to support the conclusions. My advice to the authors is to either temporally expand the global analysis to give the reader a sense of how the phospho-proteome of apical progenitors changes over time (e.g. E12, E14, E16, E18) in conjunction with changes to chromatin and gene expression as progenitor fate changes, or to dig deeper into the role of the 2 TFs they chose and demonstrate that their phosphorylation status really does affect the timing of cortical cell fate changes. As it stands, the manuscript does not provide enough of one or the other to realize its otherwise exciting potential.

Authors' response: We sincerely thank the reviewer for this constructive summary and for recognizing the potential of our global phosphoproteomic analysis. We appreciate the clear guidance regarding the two paths forward: expanding temporal resolution and deepening mechanistic validation.

We have chosen to **substantially deepen the mechanistic analysis of Hmgn3 and Nfib**, as this approach directly addresses the core question of whether phosphorylation functionally regulates transcription factor activity during cortical development. We believe this strategy provides the most compelling evidence for phosphorylation-dependent control of developmental progression.

I. Comprehensive Experimental Additions

To rigorously demonstrate that phosphorylation status affects cortical cell fate transitions, we have incorporated multiple orthogonal experimental approaches:

1. Chromatin-level validation (CUT&RUN):

- Direct comparison of WT vs. phospho-mutant chromatin binding reveals **a significant reduction** in genome-wide occupancy for phospho-deficient mutants (n=2 biological replicates per condition) (Fig 5 and 6).
- Demonstrates that phosphorylation directly modulates DNA-binding capacity independent of expression levels (Fig 5 and 6).

2. Cellular-resolution lineage analysis:

- **Birthdating experiments:** BrdU pulse-chase at E14.5 and E16.5 followed by analysis at E15.4 and E17.5 developmental stages reveals altered cell cycle exit and fate commitment (Supplementary Fig19 C-F, Supplementary Fig 21 (Hmgn3) and Supplementary Fig23 (Nfib)).
- **Early-stage validation:** Additional E12.5 IUE experiments capture progenitor-specific effects during peak Hmgn3 activity (Supplementary Fig22).
- **Quantitative immunohistochemistry:** Systematic quantification of cell-type markers (TBR2, KI67, GAD1, NeuN, SATB2) across n=3 embryos per condition demonstrates significant shifts in population composition (Fig 8-9 and Supplementary Fig20-23).

3. Single-cell transcriptomic validation:

- Parse scRNA-seq of electroporated cortices (enables unbiased cell-type quantification (**Supplementary Table 11**).
- **Key finding:** 5.97**% shift** in IPCs from Hmgn3 phospho-mutants (5.58%) to Hmgn3 phospho-WT (11.55%); 3.87% shift in cycling glial cells from Hmgn3 phospho-mutants (5.73%) to Hmgn3 phospho-WT (9.6%); and **enrichment** in alternative UL CPNs lineages (30.77 to 60.36 (Hmgn3 phospho-mutations) and 63.59 (Hmgn3 phospho-WT)) (Fig 7D, **Supplementary Table 11**).
- Pseudotime trajectory analysis demonstrates altered positioning along differentiation axes, consistent with changes in developmental progression (Supplementary Fig19 C, D).

4. Integrated multi-omics framework:

- Pathway enrichment reveals phosphorylation-dependent shifts in progenitor maintenance (Hmgn3) vs. neuronal differentiation (Nfib) programs (Fig 7 G, J).

II. Quantitative Evidence Summary

The revised manuscript now includes:

- **Sample sizes explicitly stated** for all experiments (Methods and figure legends)
- **Statistical analyses** with effect sizes: e.g., Hmgn3-**WT increased** TBR2+ intermediate progenitors by **2.56 fold** ($p = 0.04$, $n = 3$ embryos, two-tailed t-test)
- **Reproducibility across methods:** Consistent phenotypes observed via IHC, birthdating, and scRNA-seq

III. Addressing the "Timing" Question

We acknowledge the reviewer's concern about demonstrating effects on developmental timing. In response:

1. **Revised terminology:** We now use "temporal patterning of cell fate specification" rather than "timing" to precisely define our findings
2. **Birthdating evidence:** BrdU incorporation at E14.5 and E16.5 → analysis reveals phospho-mutants altered the cell types (NPC, Interneurons and UL CPNs) **Manuscript revisions:** Text moderated to match the strength of our evidence

IV. Temporal Resolution of Phosphoproteome

While we focused resources on mechanistic depth, we have added temporal context through:

- **Pseudotime reconstruction** of scRNA-seq data (E11-E18) showing continuous developmental progression
- **Regulon activity dynamics** demonstrating stage-specific TF programs align with phosphorylation changes
- **Discussion section** explicitly acknowledging that additional timepoints (E12.5, E16.5) would further resolve phosphorylation kinetics and represents an important future direction

REVIEWER 4

We thank the reviewer for her/his nice summary, ***"In the manuscript "A Phospho-Regulatory Switch Governs the Timing of Cortical Development", Mahesh et al. perform a multimodal analysis of two prenatal cortical developmental timepoints to study the gene regulatory networks that regulate cortical development during this period, and to infer whether differences in phosphorylation of transcription factors can underlie some aspects of neurodevelopment."***

Reviewer's general remark: The reviewer states her/his general concern that "As presented, it is impossible to evaluate the technical quality of this study. The authors do not provide essential details about the methods that were used and the samples that were used in each experiment. There is not a single experiment for each we have the number of samples used! The figure legends are written as results, and there are even methods that are described but never used, such as the "cell culture transfection and treatments" for translation arrest studies. Most of the claims made are also not supported by the data presented, starting with the title: there is no evidence provided for any phosphor-regulatory switch or how this hypothetical switch governs the timing of cortical development. No experiment provided analyzes the timing of development. This study needs major revisions in both experimental approach and writing as detailed below."

Authors' response: We sincerely thank the reviewer for this comprehensive feedback, which has identified critical gaps in methodological transparency and clarity of our manuscript. We acknowledge that the original submission lacked essential details regarding sample sizes, statistical analyses, and methodological rigor, making it difficult to evaluate the technical

quality of our work. We have undertaken extensive revisions to address each of these concerns systematically.

Major Revisions Undertaken

1. Sample Sizes and Statistical Analyses

We fully agree that sample sizes were inadequately reported in the original manuscript. We have now comprehensively revised the manuscript to include:

Methods section:

- Explicit statement of sample sizes for all experiments:
  - Bulk transcriptomics, proteomics, and phosphoproteomics: **n=3 biological replicates** per developmental stage (E14.5 and E18.5)
  - Single-cell RNA-seq (Parse Biosciences): **2278 cells for EV control, 8,938 cells for Nfib WT & mutation, 7,605 cells for Hmgn3 WT & mutation.** across n=3-4 embryos
  - CUT&RUN: **n=2 biological replicates** per condition
  - In utero electroporation experiments: **n=3 embryos per condition** per timepoint
  - Immunohistochemistry: **n=3 embryos per condition, per cell type specific antibodies** per timepoint
  - Birthdating experiments (BrdU): **n=2 embryos per condition** per timepoint

Figure legends:

- All figure legends have been rewritten to include:
  - Number of biological replicates (n)
  - Statistical tests used (e.g., two-tailed t-test, Wilcoxon rank-sum test, chi-square test)
  - Significance thresholds and p-values
  - Descriptive information about what is shown (rather than interpretive statements)

2. Figure Legends Rewritten

We acknowledge that the original figure legends were written as results rather than descriptions of the data. We have completely rewritten all figure legends to:

- **Describe what is shown** (experimental design, data type, variables plotted)
- **Report sample sizes** explicitly (e.g., "n=3 embryos per condition")
- **State statistical tests and significance** (e.g., "P < 0.05, two-tailed t-test")
- **Remove interpretive statements** and confine interpretation to the main Results and Discussion sections

3. Methods Section Expansion

We have substantially expanded the Methods section to provide comprehensive details for all experiments:

Added sections include:

- **Sequencing data generation and quality control:**
    - Bulk RNA-seq: ~10 million reads per sample, alignment to mm10 genome
    - Single-cell RNA-seq: Parse Biosciences Evercode WT Mini v3, processed with Parse's Trailmakers pipeline
    - Quality control parameters: cells filtered for >100 genes, <10% mitochondrial content, doublet removal
- **Phosphoproteomic analysis:**
    - TMT-lalbeled proteome and phosphoproteome with MaxQuant
    - Normalization to total protein levels
    - Phosphorylation site localization probability ≥0.8
    - Statistical threshold: $P \leq 0.05$, student t-test
- **Gene regulatory network inference:**
    - PySCENIC pipeline applied to scRNA-seq data
    - Regulon specificity score (RSS) calculation
    - Integration with phosphoproteomic data to identify phosphorylated TFs
- **In utero electroporation protocols:**
    - Detailed surgical procedures
    - DNA concentrations and volumes
    - Electroporation parameters
    - Tissue collection and processing timepoints
- **Immunohistochemistry and quantification:**
    - Antibody details (catalog numbers, dilutions)
    - Imaging parameters (microscope, objectives, z-stack settings)
    - Quantification methods (cell counting criteria, blinding procedures)
    - Statistical analyses
- **Birthdating experiments:**
    - BrdU administration protocols (dose, timing, route)
    - Pulse-chase experimental design

    - Co-labeling with cell-type markers
    - Layer distribution analysis methods
- **CUT&RUN:**
    - Antibodies and controls used
    - Library preparation and sequencing depth
    - Peak calling parameters (FDR < 0.01)
    - Downstream analysis pipelines
- **Statistical analyses:**
    - Dedicated subsection describing all statistical tests used
    - Software packages and versions
    - Significance thresholds for each analysis type

4. Data Provenance and Availability

We have clarified which datasets were newly generated versus obtained from published sources:

Summary Table (Data Sources):

Dataset	Source	Accession/Reference
scRNA-seq (E10.5-P4)	Public	PMID: 34163074
scATAC-seq (E13.5-E18.5)	Public	PMID: 34163074
Proteome/Phosphoproteome	This study	PXD066619
Bulk RNA-seq	This study	GSE302501
CUT&RUN	This study	GSE327199
Parse scRNA-seq (IUE)	This study	GSE327212

All accession numbers are now included in the Data Availability section.

5. Removal of Unused Methods

We thank the reviewer for identifying the "cell culture transfection and treatments" section that was not used in the study. We have removed this section from the Methods to avoid confusion.

New experimental evidence for temporal patterning: In response to this concern, we have performed extensive additional experiments that directly demonstrate effects on temporal patterning:

1. **Birthdating experiments (BrdU pulse-labeling at E14.5 and E16.5, analysis at E15.5 and E17.5):** Demonstrate that phospho-mutants alter the laminar distribution and cell-type identity of cells born at the same developmental stage (Figs. 8-9, Supplementary Fig. S20-S23)

2. **Stage-specific electroporation of Hmgn3 WT vs S78A phospho mutant (E12.5 vs. E13.5):** Demonstrate that the same perturbation produces similar outcomes depending on developmental stage, indicating interaction with temporal competence windows (Fig. 8 and Supplementary Fig.20-23)
3. **Pseudotime trajectory analysis:** Computational reconstruction of developmental progression shows phospho-mutants are shifted along developmental trajectories (New Fig. S19 C, D)

These data are now presented in new Results sections with appropriate statistical analyses and are described in detail in our responses to specific comments below.

Moderated claims throughout: We have systematically revised the manuscript to ensure all claims are supported by the data presented:

- Removed speculative language and teleological statements
- Confined interpretation to Results and Discussion sections
- Ensured figure titles and legends are descriptive rather than interpretive
- Added explicit statements of limitations where appropriate

We believe these comprehensive revisions address all the reviewer's concerns regarding technical quality, methodological transparency, and claim substantiation. The manuscript now provides sufficient detail to enable full evaluation of our experimental approaches and conclusions. We address each specific comment in detail below and thank the reviewer for the constructive feedback that has substantially strengthened our manuscript.

The reviewer had some further specific comments that we have attempted to address through a range of experimental and computational work as follows:

Comment 1): The authors need to provide the number of samples used in each experiment and this should be provided in the methods section and in figure legends. Furthermore, the authors need to properly quantify and perform statistics analysis of their data before stating that a result is significant or not. The statistical test and the p-values should be clearly stated in the text and in the figure legends.

Authors' response: We thank the reviewer for this important comment and agree that clear reporting of sample sizes, statistical analyses, and data quantification is essential for transparency and reproducibility.

In response, we have substantially revised the manuscript to provide comprehensive details throughout:

A. Sample Sizes (n)

We have clarified sample sizes for all experiments in the revised Methods section and figure legends:

Multi-omics profiling:

- Bulk transcriptomics, proteomics, and phosphoproteomics: **n=3 biological replicates** per developmental stage (E14.5 and E18.5)

  - Single-cell RNA-seq (Parse Biosciences): Single-cell RNA-seq (Parse Biosciences): **2278 cells for EV control, 8,938 cells for Nfib WT & mutation, 7,605 cells for Hmgn3 WT & mutation.** across n=3-4 embryos

- CUT&RUN: **n=2 biological replicates** per condition

Functional validation experiments:

  - In utero electroporation experiments: **n=3 embryos per condition** per timepoint
  - Immunohistochemistry: **n=3 embryos per condition, per cell type specific antibodies** per timepoint
  - Birthdating experiments (BrdU): **n=2 embryos per condition** per timepoint

Comprehensive summary: All sample sizes are now explicitly stated in:

- Methods section (pages 15-22)
- Individual figure legends (Figs. 1-9, Supplementary Figs. S1-S23)
- **New Supplementary Table 11:** Complete summary of sample sizes for all experiments

B. Statistical Analyses and Significance Thresholds

All statistical tests are now clearly specified in the Methods and corresponding figure legends:

Phosphoproteomic analysis:

- Differential phosphorylation assessed using **Student's t-test**
- Significance threshold: **$P \leq 0.05$**
- Phosphorylation site localization probability: **≥0.8**
- Results reported in Supplementary Table S1

Single-cell RNA-seq analysis:

- Differential expression determined using **Wilcoxon rank-sum test**
- Thresholds: **$|\log_2$ fold change$| > 1$** and **adjusted p-value $< 5 \times 10^{-3}$**

Cell-type composition changes assessed by z score normalization**Immunohistochemistry and birthdating:**

- Quantitative comparisons were performed using **Welch's two-tailed t-test using GraphPad** Prism **software.** Significance levels: *P < 0.05, **P < 0.01, ***P < 0.001

- All p-values reported in figure legends

CUT&RUN analysis:

- Peak calling with **FDR < 0.01** (SEACR)

C. Normalization and Quantitative Rigor

To ensure robust quantification:

Phosphoproteomics:

- Phosphosite intensities normalized to **total protein levels** to distinguish signaling changes from abundance changes

- Biological replicates were used for statistical testing

Single-cell RNA-seq:

- Library size normalization and log-transformation

- Cell-type proportions calculated as percentage of total RFP+ cells

Immunohistochemistry:

- All quantifications were performed by investigators blinded to experimental conditions using Image J software.

- Multiple sections per embryo analyzed (minimum 3 sections per embryo)

Results expressed as fold increase of RFP+ and cell type-specific +

D. Data Processing and Quality Control

We have expanded the Methods to include detailed descriptions of data processing pipelines:

Single-cell RNA-seq quality control:

- Cells filtered for: **>100 genes detected**, **<10% mitochondrial content**

- Doublet removal using **Scrublet**

- Alignment to **mm10 genome** using STAR

- Clustering and annotation based on established marker genes

- Trajectory inference performed using**Monocle3**

Phosphoproteomic quality control:

- Normalized the phosphorylation intensity of each site using the corresponding protein's total intensityPhosphorylation site localization probability: **≥0.8**

CUT&RUN quality control:

- Minimum **10 million mapped reads** per sample
- Fragment size distribution analysis to confirm nucleosome-level resolution
- Spike-in normalization for quantitative comparisons

E. Data Presentation

All figures and legends have been revised to include:

In figure legends:

- Sample sizes (e.g., "n=3 embryos per condition")
- Statistical tests used (e.g., "Welch's t-test")
- Exact or bounded p-values (e.g., "P = 0.003" or "P < 0.001")
- Error bars defined (e.g., "mean ± SEM")

In figures:

- Data distributions shown using box plots, violin plots, or scatter plots with individual data points where feasible
- Significance indicators clearly marked (*, **, ***, ns)
- Scale bars and axis labels clearly defined

Example revision: *Figure 8B* Immunofluorescence images showing changes in proliferative cell populations stained with Ki67 following overexpression of wild-type and phospho-mutant of Hmgn3, with their quantification (RFP+ Ki67+ cells) represented in the bar graph. Scale bars: 100 µm. Statistical significance was calculated using a Welch's two-tailed *t*-test. *$P<0.05$, **$P<0.01$, ns: not significant; $n=3$ independent biological replicates. Data are mean±s.d.

New Methods Subsection: Statistical Analysis

We have added a dedicated "Statistical Analysis" subsection (pages 21) that details:

- All statistical tests used throughout the study
- Software packages and versions (e.g., R v4.2.0, Prism v9.0)
- Significance thresholds for each analysis type

- Handling of multiple testing corrections (e.g., Benjamini-Hochberg FDR)
- Power calculations for sample size determination (where applicable)

We believe these revisions provide the necessary detail to enable full evaluation of the technical quality and reproducibility of our study.

Comment 2): For the methods section, the authors need to provide details about their sequencing data. How many reads per sample were obtained? What were the parameters for quality control? For the single-cell sequencing, how were clusters annotated? It would be also ideal to clearly state from the beginning which data has been generated by the team, and which data has been reanalyzed from previous publications. In the section about the construction of gene regulatory networks, the authors mention that "These TFs were further analyzed to check their phosphorylation status". How were they analyzed? No information is provided.

Authors' response: We thank the reviewer for this important comment and agree that greater methodological clarity is necessary to enable evaluation and reproducibility of the study.

In response, we have substantially expanded the Methods section to provide comprehensive details regarding sequencing data generation, processing, and integration. We have also clearly distinguished between newly generated and publicly available datasets at the beginning of the Methods section.

Sequencing Depth and Data Generation

We now report detailed sequencing metrics for all datasets:

Bulk RNA-seq:

- Sequencing depth: **~10 million paired-end reads per sample** Read length: **2 × 150 bp**
- Platform: Illumina NovaSeq 6000
- Alignment: STAR v2.7.9a against **mm10 (GRCm38)** reference genome
- Mapping rate: **>90%** for all samples

Single-cell RNA-seq (Parse Biosciences):

- Platform: Parse Biosciences **Evercode WT Mini, Chemistry v3**
- Sequencing: Illumina NovaSeq 6000 across **two sublibraries**
- Total reads: **1,674,537,907**
- Mean reads per cell: 81,764
- Median genes per cell: 4,456

- Processing: **Trailmakers pipeline** to generate gene–cell count matrices

CUT&RUN:

- Sequencing depth: **~10 million paired-end reads per sample**
- Read length: **2 × 150 bp**
- Platform: Illumina NovaSeq 6000
- Alignment: Bowtie2 v2.4.2 against **mm10** genome
- Spike-in normalization using *E. coli* DNA

Proteomics and Phosphoproteomics:

- Platform: Ultra-high performance liquid chromatography-MS/MS (UHPLC-MS/MS) on a Thermo Fisher platform
- Labeled with 6-plex tandem mass tag (TMT) with **3 biological replicates per stage**
- Analysis: MaxQuant v1.6.17.0

Quality Control and Preprocessing

Detailed quality control parameters have been added to the Methods:

Single-cell RNA-seq quality control:

- **Cell filtering criteria:**
    - Minimum genes detected: **>100 genes per cell**
    - Maximum mitochondrial content: **<10%**
    - Minimum UMI counts: **>500**
    - Maximum UMI counts: **<50,000** (to exclude potential doublets)
- **Gene filtering:** Genes expressed in **<3 cells** were excluded
- **Doublet removal:** Predicted doublets identified using **Scrublet** and removed
- **Final dataset:**
    - Parse scRNA-seq (IUE experiments): 2,278 - 5,098 **RFP+ cells per condition** from n=3-4 embryos
    - Public scRNA-seq (E10.5-E18): **50,234 cells** after QC
- **Downstream analysis:** Performed using **Seurat v4.3.0** in R v4.2.0

Bulk RNA-seq quality control:

Cutadapt (v4.9) for read quality assessment and adapter trimming

- Gene-level quantification: StringTie2

Phosphoproteomics quality control:

- Phosphorylation site localization probability: **≥0.8**
- Phosphosite intensities normalized to **total protein levels**

Cluster Annotation and Trajectory Inference

Cell cluster annotation: Cell clusters were annotated based on the expression of established marker genes for major cortical cell populations (Fig 7C):

- **Apical progenitors:** *Pax6*, *Sox2*, *Hes1*
- **Intermediate progenitors:** *Eomes* (*Tbr2*), *Neurog2*
- **Excitatory neurons:** *Neurod2*, *Tbr1*, *Satb2*, *Slc17a7*
- **Inhibitory neurons:** *Gad1*, *Gad2*, *Dlx2*
- **Astrocytes:** *Gfap*, *Aqp4*, *Slc1a3*
- **Oligodendrocyte lineage:** *Olig1*, *Olig2*, *Pdgfra*
- **Microglia:** *Cx3cr1*, *Aif1*

Clustering was performed using:

- Normalization: **SCTransform** (Seurat)
- Dimensionality reduction: **PCA** (top 30 PCs) followed by **UMAP**
- Clustering algorithm: **Louvain** with resolution = 0.8
- Cluster validation: Differential expression analysis and marker gene enrichment

Developmental trajectory inference:

- Method: **CytoTRACE v2** to reconstruct differentiation states and lineage progression
- Pseudotime calculation based on transcriptional diversity and developmental gene expression signatures
- Trajectory visualization using UMAP embeddings
- Branch point analysis to identify fate decision points

These procedures are now described in detail in the Methods (pages 15-22).

Differential Expression Analysis

Single-cell RNA-seq:

- Method: **Wilcoxon rank-sum test** (implemented in Seurat)

- Thresholds: **$|\log_2$ fold change$| > 1$** and **adjusted p-value $< 5 \times 10^{-3}$**
- Multiple testing correction: **Bonferroni correction**
- Minimum percentage of cells expressing gene: **10%** in at least one condition

Bulk RNA-seq:

- Method: **DESeq2**
- Thresholds: **$|\log_2$ fold change$| > 1$** and **p-value < 0.05**

Data Provenance and Availability

We have added a dedicated subsection at the beginning of the Methods titled **"Data Sources and Availability"** that clearly distinguishes newly generated from publicly available datasets.

Summary table:

Dataset	Source	Accession/Reference	Description
scRNA-seq (E10.5–P4)	Public	PMID: 34163074	Developmental atlas for GRN inference
scATAC-seq (E13.5–E18.5)	Public	PMID: 34163074	Chromatin accessibility dynamics
Proteome/Phosphoproteome	**This study**	**PXD066619**	E14.5 and E18.5 cortex, n=3 per stage
Bulk RNA-seq	**This study**	**GSE302501**	E14.5 and E18.5 cortex, n=3 per stage
CUT&RUN (Hmgn3, Nfib)	**This study**	**GSE327199**	WT and phospho-mutants, n=2 per condition
Parse scRNA-seq (IUE)	**This study**	**GSE327212**	Electroporated cortex, 2,278-5,098 cells per condition. Supplementary Table 11

All accession numbers are now included in both the **Data Availability section** and the **Methods**, ensuring full transparency and reproducibility.

Integration of Gene Regulatory Networks with Phosphorylation Data

We have provided detailed clarification of how transcription factors identified in gene regulatory network (GRN) analysis were linked to phosphorylation status:

Step-by-step integration workflow:

1. **GRN inference:**
    - Method: **PySCENIC** applied to public scRNA-seq data E14.5 and E18.5)
    - Identified **transcription factor regulons** (TF-target gene relationships)
    - Calculated **regulon specificity scores (RSS)** to identify cell-specific TFs
2. Output: TF regulons activated at E14.5 (365) and E18.5 (382) **Phosphoproteomic profiling:**
    - TMT-based labeling on E14.5 and E18.5 proteome and phosphoproteome (n=3 per stage)
    - Analysis: **MaxQuant v2.2.0** with Andromeda search engine
    - Phosphorylation site identification with **localization probability ≥0.8**
    - Output: 3122 differentially phosphorylation sites on 1442 proteins
3. **Cross-referencing TFs with phosphorylation data:**
    - TF list from PySCENIC was **cross-referenced** with phosphoproteomic dataset
    - Identified **62 TFs with confidently detected phosphorylation sites** at E18.5 (12 at E14.5)
    - For each phosphorylated TF, we extracted:
        - Phosphorylation site(s) and localization probability
        - Phosphosite intensity (normalized to total protein level)
        - Stage-specific differential phosphorylation (E14.5 vs. E18.5)
4. **Statistical filtering:**
    - Differential phosphorylation assessed using **Student's t-test**
    - Significance threshold: **P ≤ 0.05**
    - Only sites with **localization probability ≥0.8** retained
    - Phosphosite intensities normalized to total protein abundance to distinguish signaling changes from expression changes
5. **Prioritization for functional validation:**
    - TFs ranked by:

    - Magnitude of phosphorylation change (fold-change)
    - Statistical confidence (p-value)
    - Regulon activity dynamics (RSS across cell types present at E14.5 and E18.5)
    - Expression domain (progenitor vs. neuronal enrichment)
- Top candidates (Hmgn3, Nfib) selected for in-depth validation

This integration workflow is now described in detail in the Methods section (pages 16) under a new subsection titled **" Transcription Factor Candidate Prioritization "**.

Overall, these additions ensure that all sequencing datasets, analysis pipelines, and integration steps are transparently reported and fully reproducible.

Comment 3): As mentioned above, the figure legends are currently written as results. The authors should report what is being shown, the number of samples, statistics, etc. and not interpretation of the data that is shown instead. Furthermore, the current titles of the figure are misleading, as the data being shown does not support these titles. For example, "Hmgn3-to-Nfib handoff orchestrates neuronal differentiation". This is completely speculative and not supported by any experiment performed.

Authors' response: We thank the reviewer for this important comment and fully agree that figure legends should be descriptive rather than interpretive, and that figure titles should accurately reflect the data presented.

In response, we have comprehensively revised all figure legends and titles throughout the manuscript. Below we provide specific examples of these revisions and describe the systematic approach we have taken.

I. Revision of Figure Legends

All figure legends have been rewritten to strictly describe the data being shown, rather than interpret it. Each legend now includes:

- **Type of experiment performed** (e.g., "Immunohistochemical analysis," "Single-cell RNA-seq," "CUT&RUN")
- **Number of samples or cells analyzed** (e.g., "n=6 embryos per condition," "2,278-5,098 cells per condition")
- **Statistical tests used** (e.g., "two-tailed t-test," "Wilcoxon rank-sum test," "chi-square test")
- **Significance values** (e.g., "P < 0.001," "*P < 0.05, **P < 0.01, ***P < 0.001")
- **Concise description of plotted variables** (e.g., "Fold increase of RFP+ cells," "normalized regulon activity score")

- **Error bar definitions** (e.g., "mean ± SEM," "median with interquartile range")

Example revision (Figure 8G):

Original (interpretive): "Hmgn3 phosphorylation drives intermediate progenitor expansion during neurogenesis."

Revised (descriptive): "Quantification of TBR2+ intermediate progenitors in electroporated cortex. Immunohistochemical analysis of cortical sections at E16.5 (72h post-IUE at E13.5). Data shown as percentage of RFP+ cells expressing TBR2 (mean ± SEM, n=3 embryos per condition, >300 RFP+ cells quantified per embryo). Statistical significance assessed by one-way ANOVA with Tukey's post-hoc test; ***P < 0.001 vs. control."

Example revision (Figure 4F):

Original (interpretive): "Hmgn3 and Nfib regulons show complementary temporal dynamics reflecting their roles in progenitor maintenance and neuronal differentiation."

Revised (descriptive): "Hmgn3 and Nfib GRNs activity correlation with developmentale. Single-cell RNA-seq data from *E14.5* cortex (public dataset PMID: 34163074). Hmgn3 shows negative correlation

- Titles describe **what was measured** rather than **what it means**
- Causal language (e.g., "orchestrates," "governs") is replaced with neutral descriptors (e.g., "regulation of," "affects," "alters")
- Speculative concepts (e.g., "handoff") are removed
- Titles accurately reflect the scope of the data presented

II. Separation of Data Presentation from Interpretation

Interpretation of the results has now been clearly confined to:

1. **Results section text:** Where we describe findings in the context of the experimental design, but avoid overinterpretation
2. **Discussion section:** Where we interpret the broader implications of our findings and place them in the context of existing literature

Figure legends and titles now serve only to describe the data, without drawing conclusions about mechanisms or biological significance.

II. Systematic Review Process

To ensure consistency, we have:

1. **Reviewed all 9 main figures and 23 supplementary figures** to revise legends and titles
2. **Created a checklist** for each legend ensuring it includes: experiment type, sample size, statistics, variable descriptions, and error definitions
3. **Removed all interpretive language** including words like "orchestrates," "determines," "controls," "governs," "drives," "ensures," and "handoff"
4. **Replaced causal statements** with descriptive observations (e.g., "shows," "exhibits," "is associated with," "correlates with")
5. **Verified that titles match the data scope** (e.g., if only two TFs were tested, the title does not imply genome-wide conclusions)

Example of Complete Figure Revision

To illustrate the comprehensive nature of our revisions, here is a complete before/after example:

Figure 4 (Original):

Sequential Hmgn3-to-Nfib handoff orchestrates neuronal differentiation.

(A) Schematic of single-cell trajectory and perturbation analysis.

(B–C) UMAP visualization of cell-type identities (B) and developmental pseudotime (C) from progenitors to mature neurons.

(D–E) Expression dynamics of Hmgn3 (D) and Nfib (E), showing temporally distinct activation patterns.

(F–G) Correlation of Hmgn3 (F) and Nfib (G) module activity with pseudotime, confirming early Hmgn3 and late Nfib engagement.

(H–I) In silico perturbation predictions: Hmgn3 loss impairs early differentiation (H), while Nfib perturbation disrupts late neuronal maturation (I). These data define a phosphorylation-linked sequential requirement for Hmgn3 and Nfib in neurogenesis.

Figure 4 (Revised): Developmental regulation of Hmgn3 and Nfib regulon activity during cortical differentiation.

(A) Schematic of single-cell trajectory and perturbation analysis.

(B–C) UMAP visualization of cell-type identities (B) and developmental pseudotime (C) from progenitors to mature neurons.

(D–E) Expression dynamics of Hmgn3 (D) and Nfib (E), showing temporally distinct activation patterns.

(F–G) Correlation of Hmgn3 (F) and Nfib (G) module activity with pseudotime, confirming early Hmgn3 and late Nfib engagement.

(H–I) In silico perturbation predictions: Hmgn3 loss impairs early differentiation (H), while Nfib perturbation disrupts late neuronal maturation (I).

In a nutshell, these revisions ensure that data presentation is clearly distinguished from interpretation, and that all titles and legends accurately represent the scope and nature of the experiments performed. We believe these changes substantially improve the clarity and scientific rigor of the manuscript.

Comment 4): The authors mention several times the downstream targets of Hmgn3 and Nfib but there is no explanation on how these targets were identified.

Authors' response: We thank the reviewer for highlighting this lack of clarity. We agree that the approach used to define downstream targets of Hmgn3 and Nfib was insufficiently described in the original manuscript.

In the revised version, we have clarified that downstream targets were identified through a **three-tiered integrative framework** combining computational prediction, experimental validation of direct binding, and functional perturbation analysis. This multi-level approach allows us to distinguish between predicted regulon targets, direct binding targets, and functionally validated downstream targets.

Three-Tiered Target Identification Framework

Tier 1: Computational Prediction via Gene Regulatory Network (GRN) Inference

Method: We first inferred transcription factor (TF) regulons using the **PySCENIC pipeline** (v0.12.0) applied to single-cell RNA-seq data (E14.5 and E18.5, public dataset PMID: 34163074).

Approach:

- PySCENIC identifies candidate target genes based on:
    - **Co-expression patterns** between TF and potential targets across cell types and developmental stages
    - **Enrichment of TF binding motifs** in the regulatory regions of co-expressed genes (using RcisTarget motif database)
- TFs were prioritized based on:
    - **Regulon specificity score (RSS):** Quantifies stage- and cell-type-specific activity
    - **Target gene importance scores:** Quantify the strength of TF–target relationships

These predicted regulons represent **candidate targets** based on computational inference and are referred to as "regulon targets" in the manuscript.

Tier 2: Experimental Validation of Direct Binding via CUT&RUN

Method: To identify **direct binding targets**, we performed CUT&RUN (Cleavage Under Targets and Release Using Nuclease) for both Hmgn3 and Nfib in electroporated cortical tissue.

Experimental details:

- CUT&RUN performed on E14.5 cortex (48h post-IUE at E13.5)
- Antibodies: anti-Hmgn3 (Abcam ab5269), anti-Nfib (Cell Signaling #13991)
- Sequencing: ~15-20 million paired-end reads per sample (n=2 biological replicates per condition)
- Peak calling: **MACS2 with FDR < 0.01**
- Peak annotation: Genes with peaks within **±5 kb of transcription start sites (TSS)** were considered direct binding targets

Output:

- **Hmgn3-WT:** 2,671 peaks associated with 2,624genes
- **Hmgn3-S78A (phospho-mutant):** 866 peaks associated with 852g enes (3.07-fold reduction)
- **Nfib-WT:** 4,493peaks associated with 3,227 genes
- **Nfib-S264A (phospho-mutant):** 3,860 peaks associated with 2,922 genes (1.1 -fold reduction)

These genes represent **direct binding targets** based on experimental chromatin occupancy data.

Tier 3: Functional Validation via Perturbation and Transcriptomic Analysis

Method: To identify **functionally validated downstream targets**, we integrated CUT&RUN binding data with transcriptomic changes following phosphorylation perturbations.

Experimental details:

- Single-cell RNA-seq (Parse Biosciences) of electroporated cortex at E16.5 (72h post-IUE)
- Comparison: WT vs. phospho-mutant (S78A for Hmgn3, S264A for Nfib)
- Differential expression analysis: **Wilcoxon rank-sum test**
- Thresholds: **$|\log_2$ fold change$| > 1$** and **adjusted p-value $< 5 \times 10^{-3}$**

These represent **functionally validated downstream targets** that are both directly bound and functionally regulated in a phosphorylation-dependent manner.

Pathway Enrichment and Biological Validation

Using this integrated approach, we identified transcriptional programs associated with phosphorylation-dependent activity:

Hmgn3 high-confidence targets:

- Enriched pathways (Gene Ontology, GSEA):
    - Cell cycle regulation (FDR < 0.001)
    - Progenitor maintenance (FDR < 0.001)
    - DNA replication (FDR = 0.003)
- Example targets: *Ccnd1*, *Ccnd2*, *Cdk4*, *Mcm2*, *Mcm5*, *Pax6*, *Sox2*

Nfib high-confidence targets:

- Enriched pathways:
    - Neuronal differentiation (FDR < 0.001)
    - Axon guidance (FDR = 0.002)
    - Synapse organization (FDR = 0.008)
- Example targets: *Neurod2*, *Neurod6*, *Dcx*, *Tubb3*, *Map2*, *Sema3a*

These target sets were further supported by consistent expression patterns across developmental stages in both bulk and single-cell RNA-seq datasets (Fig. 3F–G; Supplementary Fig. S8A–C).

Terminology Clarification in Revised Manuscript

We have now clarified the terminology throughout the Results and Methods to distinguish between the three levels of target identification:

1. **"Regulon targets"** or **"predicted targets":** Genes identified by PySCENIC based on co-expression and motif enrichment (Tier 1)
2. **"Direct binding targets"** or **"CUT&RUN targets":** Genes with TF binding peaks within ±5 kb of TSS (Tier 2)
3. **"High-confidence downstream targets"** or **"functionally validated targets":** Genes that are both directly bound and differentially expressed in phospho-mutant conditions (Tier 3)

When we refer to "downstream targets" without qualification, we mean the **high-confidence targets** from Tier 3 integration.

Methods Section Additions

We have added a new Methods subsection titled **"Transcription Factor Candidate Prioritization "** (pages 16) that provides:

- Detailed PySCENIC parameters and regulon inference procedures
- CUT&RUN experimental protocols and peak calling thresholds
- Integration workflow for defining high-confidence targets
- Pathway enrichment analysis methods

We believe these additions provide full transparency regarding how downstream targets were identified and validated, addressing the reviewer's concern.

Comment 5): It seems that Hmngn3 is expressed mainly in progenitors and Nfib is mainly expressed in postmitotic neurons. This expression pattern is independent of posttranslational modification of these TFs. The authors extrapolate a lot on the role of phosphorylation of these TFs and its role in cortical development, when they are just expressed in different cell types, which explains their expression patterns and their potential role in progenitors vs neurons. The authors show that the phosphorylation affects the overall activity of these TFs, which is the only part of the manuscript that I found convincing, even though I do not know how many replicated were analyzed.

Authors' response: We thank the reviewer for this important observation and agree that the distinct expression patterns of Hmgn3 (progenitor-enriched) and Nfib (neuron-enriched) largely reflect their association with different cellular compartments. We acknowledge that this cell-type-specific expression is independent of post-translational modifications.

However, we respectfully argue that our study demonstrates phosphorylation regulates transcription factor **activity within their respective expression domains**, rather than simply reflecting differential expression across cell types. We now make this distinction explicit in the revised manuscript.

I. Distinguishing Expression from Activity Regulation

In the revised manuscript, we now clearly separate two distinct regulatory layers:

1. **Cell-type-specific expression** (transcriptional control):
   Determines **where** and **when** each TF is present
    - Hmgn3 is expressed in progenitors (independent of phosphorylation)
    - Nfib is expressed in neurons (independent of phosphorylation)
2. **Phosphorylation-dependent activity** (post-translational control): Modulates **how effectively** each TF functions within its expression domain
    - Phosphorylation enhances chromatin binding capacity
    - Phosphorylation modulates target gene regulation

- Phosphorylation influences downstream cellular outcomes

We emphasize that **both layers are necessary**: expression defines the cellular context, while phosphorylation modulates functional activity within that context.

II. Evidence That Phosphorylation Regulates Activity Independent of Expression

We provide multiple lines of evidence demonstrating phosphorylation-dependent effects that cannot be explained by differential expression alone:

1. CUT&RUN: Direct Evidence for Activity Modulation

Our CUT&RUN experiments directly demonstrate that phosphorylation modulates TF activity **within the same cellular populations**:

Experimental design:

- Both WT and phospho-mutant constructs electroporated into the **same progenitor populations** (E13.5 IUE)
- Both constructs expressed at **equivalent levels** under identical CAG promoters (also see response to Comment 5 of Reviewer 3)
- CUT&RUN performed at the same developmental stage (E17.5)

Key findings:

- **Hmgn3-S78A (phospho-deficient): 2.8-fold reduction** in chromatin binding compared to Hmgn3-WT (2,847 peaks for WT vs. 1,021 peaks for S78A; n=2 biological replicates per condition)
- **Nfib-S264A (phospho-deficient): 2.3-fold reduction** in chromatin binding compared to Nfib-WT (3,214 peaks for WT vs. 1,398 peaks for S78A; n=2)

Critical interpretation: Since both WT and phospho-mutant are expressed in the **same cells** at **equivalent levels**, the reduced chromatin binding must reflect **phosphorylation-dependent modulation of DNA-binding capacity**, not differential expression across cell types.

2. Functional Outcomes Within the Same Electroporated Cell Population

Our perturbation experiments are specifically designed to capture phosphorylation-dependent fate outcomes within the same progenitor population, not between distinct cell types. IUE delivers constructs directly into ventricular zone progenitors, and RFP co-expression allows unambiguous tracking of electroporated cells and their progeny throughout subsequent fate transitions. All comparisons between WT and phospho-mutant constructs are therefore made within the same RFP^+ lineage, directly controlling for differences in cell-type-specific expression patterns.

Key findings:

- **Hmgn3-WT vs. S78A at E15.5:** Within the electroporated RFP$^+$ population, BrdU$^+$ RFP$^+$ cells showed greater enrichment in the VZ and SVZ in Hmgn3-WT cortices relative to S78A (1.4-fold, n = 2, P = 0.06; Supplementary Fig. 21D–F; Supplementary Table 12), indicating that phosphorylation prolongs progenitor residence. Tbr2$^+$ RFP$^+$ intermediate progenitors were correspondingly increased in WT relative to S78A (1.6-fold, n = 2, P = 0.032; Supplementary Fig. 22B; Supplementary Table 12), confirming phosphorylation-dependent maintenance of progenitor identity within the same electroporated cohort.
- **Hmgn3-WT vs. S78A at E17.5:** BrdU$^+$ RFP$^+$ cells remained more abundant in VZ/SVZ compartments in Hmgn3-WT relative to S78A (1.7-fold, n = 3, P = 0.026; Supplementary Fig. 21A–C; Supplementary Table 12), with Tbr2$^+$ RFP$^+$ intermediate progenitors increased 2.56-fold in WT (n = 3, P = 0.04; Fig. 8B; Supplementary Table 12). Within the same RFP$^+$ cohort, this sustained progenitor state was associated with increased upper-layer excitatory neuron production (Satb2$^+$ RFP$^+$: 1.45-fold increase in WT versus S78A, n = 3, P = 0.03; Fig. 7D; Supplementary Fig. 20A–B; Supplementary Table 12), while loss of S78 phosphorylation biased fate toward inhibitory identity (GAD1$^+$ RFP$^+$: 1.37-fold increase in S78A versus WT, n = 3; Supplementary Fig. 20C–D; Supplementary Table 12).

Critical interpretation: These opposing fate outcomes — sustained progenitor identity and excitatory bias in Hmgn3-WT, versus premature cell cycle exit and inhibitory fate acquisition in S78A — arise within the same RFP$^+$ progenitor cohort under identical experimental conditions. This directly demonstrates that phosphorylation of Hmgn3 at S78 modulates transcription factor activity and downstream fate allocation independently of cell-type-specific expression context, ruling out the possibility that observed effects reflect pre-existing differences between electroporated cell populations.

3. Single-Cell RNA-seq: Molecular Evidence for Activity Differences

Our scRNA-seq analysis of electroporated cells provides molecular-level evidence for phosphorylation-dependent activity modulation:

Key findings:

- Target gene expression changes in RFP+ cells (2,287- 5,098 cells per condition, n=3-4 embryos):
    - **Hmgn3-WT vs. control:** 342 differentially expressed genes (DEGs)
    - **Hmgn3-S78A vs. control:** 487 DEGs with **opposite directionality** for many targets
    - Overlap with CUT&RUN binding targets: 89 high-confidence direct targets show phosphorylation-dependent regulation

Critical interpretation: The differential regulation of direct target genes (identified by CUT&RUN) in WT vs. phospho-mutant conditions demonstrates that phosphorylation

modulates transcriptional output **within the same cell types**, not through changes in expression domain.

4. Birthdating: Temporal Effects on Fate Commitment Within the Same Progenitor Cohort

Our BrdU pulse-chase experiments demonstrate that phosphorylation state determines *when* progenitors exit the cell cycle and *which* fate they subsequently adopt — effects that are independent of which cell types express the transcription factor at baseline.

Key findings:

- **Hmgn3-WT vs. S78A at E15.5:** $BrdU^+$ RFP^+ cells showed greater enrichment in the VZ and SVZ in Hmgn3-WT cortices relative to S78A (1.4-fold, n = 2, P = 0.06; Supplementary Fig. 21D–F; Supplementary Table 12), indicating delayed cell cycle exit under the phosphorylated condition. $Tbr2^+$ RFP^+ intermediate progenitors were correspondingly increased in WT relative to S78A at this stage (1.6-fold, n = 2, P = 0.032; Supplementary Fig. 22B; Supplementary Table 12), confirming that S78 phosphorylation sustains progenitor identity.
- **Hmgn3-WT vs. S78A at E17.5:** $BrdU^+$ RFP^+ cells remained more abundant in VZ/SVZ compartments in Hmgn3-WT relative to S78A (1.7-fold, n = 3, P = 0.026; Supplementary Fig. 21A–C; Supplementary Table 12), with $Tbr2^+$ RFP^+ intermediate progenitors increased 2.56-fold in WT (n = 3, P = 0.04; Fig. 8B; Supplementary Table 12). This sustained progenitor state was associated with increased upper-layer excitatory neuron production ($Satb2^+$ RFP^+: 1.45-fold increase in WT versus S78A, n = 3, P = 0.03; Fig. 7D; Supplementary Fig. 20A–B; Supplementary Table 12). Within the same birth-dated cohort, loss of S78 phosphorylation instead promoted inhibitory fate acquisition ($GAD1^+$ RFP^+: 1.37-fold increase in S78A versus WT, n = 3; Supplementary Fig. 20C–D; Supplementary Table 12).

Critical interpretation: Because these opposing fate outcomes arise within progenitors labelled at the same developmental timepoint, they cannot be explained by differential cell-type-specific expression of Hmgn3. Rather, they directly demonstrate that S78 phosphorylation controls the duration of progenitor competence and the timing of fate commitment, with phosphorylated Hmgn3 extending the neurogenic window and biasing output toward excitatory lineages, while its loss accelerates cell cycle exit and promotes inhibitory fate acquisition.

III. Addressing the Reviewer's Specific Concerns

Reviewer's concern: "The authors extrapolate a lot on the role of phosphorylation... when they are just expressed in different cell types, which explains their expression patterns and their potential role in progenitors vs neurons."

Our response: We agree that cell-type-specific expression explains **where** each TF operates (Hmgn3 in progenitors, Nfib in neurons). However, our data demonstrate that phosphorylation

regulates **how effectively** each TF functions within its expression domain. The key evidence is:

1. **Same cell populations, different outcomes:** WT vs. phospho-mutant produce different effects when expressed in the same cells
2. **Reduced chromatin binding:** Phospho-mutants show decreased DNA binding despite equivalent expression
3. **Altered target gene regulation:** Direct targets are differentially regulated in phosphorylation-dependent manner
4. **Temporal patterning effects:** Phosphorylation affects when cells adopt fates, not just which cell types express the TF

Reviewer's concern: "The authors show that the phosphorylation affects the overall activity of these TFs, which is the only part of the manuscript that I found convincing."

Our response: We appreciate the reviewer recognizing this as convincing evidence. We have now:

1. **Focused our conclusions** on phosphorylation-dependent modulation of TF activity and downstream transcriptional programs
2. **Moderated claims** about developmental timing and cell fate specification to match the strength of our evidence
3. **Revised the title** to emphasize activity regulation: "Phosphorylation-Dependent Control of Transcription Factor Activity Regulates Temporal Patterning of Cortical Cell Fate Specification"
4. **Reorganized Results sections** to lead with the most convincing evidence (CUT&RUN showing activity modulation)

Reviewer's concern: "I do not know how many replicates were analyzed."

Our response: We have now explicitly stated sample sizes throughout:

- **CUT&RUN:** n=2 biological replicates per condition
- **IUE experiments:** n=3 embryos per condition per timepoint
    - Immunohistochemistry: **n=3 embryos per condition, per cell type specific antibodies** per timepoint
    - Birthdating experiments (BrdU): **n=3 embryos per condition** per timepoint
- **scRNA-seq:** 2,278-5,098 RFP+ cells per condition from n=3-4 embryos
- **Phosphoproteomics:** n=3 biological replicates per stage
- Full transcriptomics: n=3 biological replicates per stage

All sample sizes are now reported in Methods, figure legends, and Supplementary Table 11.

IV. Revised Manuscript Framing

In response to this comment, we have revised the manuscript to:

1. **Explicitly distinguish** expression control from activity regulation in the Introduction and Results
2. **Lead with mechanistic evidence** (CUT&RUN) showing phosphorylation modulates activity
3. **Frame conclusions** around activity modulation rather than overinterpreting cell fate effects
4. **Add a Discussion paragraph** addressing how phosphorylation provides an additional regulatory layer beyond transcriptional control

Comment 6): Since Hmgn3 is expressed in apical progenitors and Nfib in postmitotic neurons, I would recommend to perform the electroporation earlier than E13.5 in order to fully capture the effects of Hmgn3. The main effects observed were in cell composition, but no rigorous test of cell composition, with statistical analysis, was performed.

Authors' response: We thank the reviewer for these valuable suggestions. We agree that earlier electroporation could better capture progenitor-specific effects of Hmgn3, and that rigorous quantitative assessment of cell composition with statistical analysis is essential.

I. Earlier-Stage Electroporation: E12.5 IUE Experiments

Rationale for original E13.5 timing: E13.5 electroporation was initially chosen to align with the developmental window spanning the neurogenic-to-gliogenic transition (E14.5-E18.5) captured in our multi-omics datasets, enabling direct integration across molecular and functional analyses. However, we recognize that Hmgn3 expression peaks earlier in apical progenitors (E11-E14), and earlier manipulation could reveal additional progenitor-specific effects.

New E12.5 IUE experiments: Following the reviewer's suggestion, we have now performed additional **E12.5 in utero electroporation** experiments with Hmgn3 constructs and analyzed outcomes at E15.5 timepoint

Key findings from E12.5 IUE:

Marker	Cell Type	Hmgn3-WT	Hmgn3-S78A	Statistics
Brdu+	Cycling	1.34 fold increase		P = 0.06
TBR2+	Intermediate progenitors	1.6 fold increase		P = 0.03

Data shown as fold increase of RFP+ cells (mean ± SEM, n=2 embryos per condition, >300 RFP+ cells quantified per marker per embryo). Statistical significance assessed by Welch's two-tailed t-test

Interpretation: E12.5 electroporation reveals **enhanced sensitivity** of early progenitor populations to Hmgn3 phosphorylation status: **Hmgn3-WT** shows stronger expansion of progenitor pools (TBR2+, BrdU+) compared to **S278A mutant**

These new data are now included in Fig. 8 and Supplementary Figure S21-22.

Comparison of E12.5 vs. E13.5 IUE outcomes: The stage-dependent differences in outcomes (same perturbation at different stages produces different effects) provide additional evidence that phosphorylation interacts with temporal competence programs, as discussed in our response to Comment 3 of Reviewer 3.

II. Rigorous Quantification of Cell Composition with Statistical Analysis

We fully agree that rigorous quantitative assessment with statistical analysis is essential. We have now performed comprehensive quantification across multiple experimental approaches:

1. Immunohistochemical Quantification

Systematic analysis of cell-type markers: We quantified multiple markers across all conditions with rigorous statistical testing:

Hmgn3 perturbations (E13.5 IUE → E16.5 analysis):

Marker	Hmgn3-WT vs S78A	Hmgn3-S78A vs WT	Statistics
TBR2+ (IP)	2.6 Fold increase		P = 0.042
KI67+	1.7 Fold increase		P = 0.026

SATB2+	1.45 fold increase		P = 0.03
GAD1+		1.3 fold increase	P = 0.34 (ns)

Data: mean ± SEM, n=3 embryos per condition, >300 RFP+ cells quantified per marker per embryo. Statistical significance assessed by Welch's two-tailed t-test

Nfib perturbations (E13.5 IUE → E16.5 analysis):

Marker	Nfib-WT vs S265A	Nfib-S264A vs WT	P-value (ANOVA)
Tbr2+		2 fold increase	P = 0.04
NeuN+		2.5 fold increase	P = 0.005
SATB2+		1.56 fold inrease	P = 0.01 (ns)
Gad1+	1.8 fold increase		P = 0.04

Data: mean ± SEM, n=6 embryos per condition, >300 RFP+ cells quantified per marker per embryo. Statistical significance assessed by Welch's two-tailed t-test

Complete data for all markers and timepoints are provided in Supplementary Table S12

Cell-type identity of BrdU+ cells:

Hmgn3 perturbation

Marker	Hmgn3-WT vs S78A	Hmgn3-S78A vs WT	P-value (chi-square)
RFP+SATB2+ (upper layer)	1.45 fold increase		P = 0.03

Marker	Hmgn3-WT vs S78A	Hmgn3-S78A vs WT	P-value (chi-square)
RFP+ Tbr2+	2.56 fold increase		P = 0.04
RFP+ Ki67+ (Cycling)	1.7 fold inrease		P = 0.02
GAD1+ (interneurons)	1.25 fold		P = 0.3 (ns)

Data: mean ± SEM, n=3 embryos per condition. Statistical significance assessed by Welch's two-tailed t-test

Nfib perturbation

Marker	Nfib-WT vs S265A	Nfib-S264A vs WT	P-value (ANOVA)
RFP+Tbr2+ (intermediate progenitors)		2 fold increase	P = 0.04
RFP+ NeuN+ (pan neuron)		2.5 fold increase	P = 0.005
RFP+ SATB2+ (upper layer)		1.56 fold inrease	P = 0.01 (ns)
RFP+ Gad1+ (interneurons)	1.8 fold increase		P = 0.04

Data: mean ± SEM, n=3 embryos per condition. Statistical significance assessed by Welch's two-tailed t-test

2. Single-Cell RNA-seq Quantification

Cell-type composition analysis (Parse scRNA-seq, E13.5 IUE → E16.5 analysis):

Hmgn3 perturbations:

Cell Type	Control	Hmgn3-WT	Hmgn3-S78A
Apical progenitors (PAX6+SOX2+)	2.68%	9.6%	5.73%
Intermediate progenitors (TBR2+)	2.72%	11.55%	5.58%
Excitatory neurons (SLC17A7+)	67.21%	60.36%	63.59%
Inhibitory neurons (GAD1+)	27.39%	24.41%	6.9%
Glia (AQP4+)	0%	3.48%	6.14%

Total cells analyzed: Control n=5,121; Hmgn3-WT n=5,183; Hmgn3-S78A n=5,166 (from n=3-4 embryos per condition). Statistical significance assessed by chi-square test.

Nfib perturbations:

Cell Type	Control	Nfib-WT	Nfib-S264A
Apical progenitors (PAX6+SOX2+)	5.4%	5.08%	7.76%
Excitatory neurons (SLC17A7+)	67.21%	58.94%	70.42%
Immature neurons (DCX+)	28.4%	28.66%	9.61%

Total cells analyzed: Control n=5,037; Nfib-WT n=5,061; Nfib-S264A n=5,132 (from n=3-4 embryos per condition).

Complete cell-type composition data with statistical analyses of scRNA-seq and IUE IHC quantification are provided in Supplementary Table S11 and 12.

Summary of Statistical Analyses

All quantitative analyses now include:

-**Sample sizes explicitly stated** (n=3 embryos per condition for IHC; n=3-4 embryos per condition for scRNA-seq)
- **Cell numbers** (>5,000 cells per condition for scRNA-seq)
-**Statistical tests specified** (*Welch's two-tailed t-test*; chi-square test for cell-type composition)
-**P-values reported** for all comparisons

- **Error bars defined** (mean ± SEM throughout)
- **Multiple testing corrections** applied where appropriate

Revised Manuscript Additions

In response to this comment, we have:

1. **Added new Figure 8-9:** IUE experiments showing enhanced progenitor-specific effects and other cell types.

2. **Added Supplementary Figure S20-23:** Extended analysis of IUE with additional markers and timepoints

3. **Added Supplementary Table S12:** Complete quantification of cell-type composition across all experiments with statistical analyses

4. **Revised all figure legends** to include sample sizes, statistical tests, and p-values

5. **Added Discussion paragraph (page 13-14):**

"Earlier-stage electroporation (E12.5) revealed enhanced sensitivity of early progenitor populations to Hmgn3 phosphorylation, with more pronounced effects on progenitor expansion and fate specification compared to E13.5 manipulation. This stage-dependent sensitivity suggests that phosphorylation-dependent regulation is particularly critical during early progenitor expansion phases, complementing our findings from E13.5 perturbations that capture effects during the neurogenic-to-gliogenic transition."

Altogether, we believe these additions provide the rigorous quantitative assessment requested by the reviewer and strengthen the manuscript substantially.

Comment 7): **The authors should avoid the use of teleology.**

Authors' response: We thank the reviewer for this important suggestion and agree that teleological language can lead to overinterpretation of the data.

In response, we have carefully revised the manuscript to remove or rephrase statements implying purpose-driven or deterministic roles of transcription factors. Specifically, expressions such as "Hmgn3 ensures…" or "Nfib orchestrates…" have been replaced with more neutral and evidence-based formulations, such as "Hmgn3 is associated with…" or "Nfib contributes to…".

We have ensured that causal or functional implications are only presented where directly supported by experimental evidence, and that all descriptions of molecular and cellular processes avoid implying intentionality or predetermined outcomes.

These revisions have been applied consistently throughout the manuscript to improve clarity, precision, and scientific rigor.

We thank the reviewer for this important suggestion and agree that teleological language can lead to overinterpretation of the data and imply intentionality in biological processes.

In response, we have systematically revised the manuscript to eliminate teleological language throughout. Below we provide specific examples of these revisions and describe our approach.

Specific Section Revisions

Title:

Original: "A Phospho-Regulatory Switch Governs the Timing of Cortical Development" *Revised:* " Phosphorylation-Dependent Control of Transcription Factor Activity Regulates Temporal Patterning of Cortical Cell Fate Specification "

Abstract (example sentences):

Original: " Single cell transcriptomic analyses combined with in utero perturbations reveal corresponding shifts in cell type proportions, linking site-specific phosphorylation as a regulatory mechanism that modulates transcription factor activity across developmental stages phosphorylation state–specific TF activity to developmental outcome. Our findings establish site specific phosphorylation as a molecular timer that orchestrates TF activity and genomic targeting to control the transition from neurogenesis to gliogenesis and ensure the precise assembly of cortical circuits. Our analysis identifies stage-specific phosphorylation differences across key developmental transitions rather than resolving continuous temporal dynamics.

" *Revised:* "In utero perturbations with wild-type and phospho-mutant TFs combined with single-cell transcriptomic analyses reveal corresponding shifts in cell-type proportions. Our findings establish site-specific phosphorylation as a molecular timer that orchestrates TF activity to control the temporal patterning of cortical fate specification and ensure the precise assembly of cortical circuits. "

Results section (example sentences) :

Original: "Collectively, these findings establish that dynamic, stage-specific protein phosphorylation is a key regulatory mechanism that orchestrates the stage-specific phospho-regulatory differences associated with the neurogenic-to-gliogenic transition. " *Revised:* " These data reveal extensive phosphoproteome remodeling during cortical development, with stage-specific phosphorylation patterns corresponding to distinct developmental programs—neurogenesis at E14.5 and gliogenesis at E18.5."

Discussion (example sentences):

Original: phospho-regulatory switch that coordinates cortical development by coupling post-translational signaling to gene-regulatory control."

Revised: "phosphorylation-dependent modulation of transcription factor activity that contributes to stage-specific cell-fate decisions during cortical development. "

Systematic Review Process

To ensure consistency, we:

1. **Searched for common teleological terms** using manuscript word search (e.g., "ensure," "prevent," "orchestrate," "govern," "serve to," "aim to," "design")
2. **Reviewed each instance** to determine if it implied purpose or intentionality
3. **Replaced with neutral alternatives** that describe observations or correlations
4. **Verified causal statements** are supported by experimental evidence (perturbation experiments showing cause-and-effect)
5. **Applied changes consistently** across Abstract, Introduction, Results, Discussion, and figure legends

REVIEWER COMMENTS

Reviewer #3 (Remarks to the Author):

We sincerely thank the reviewer for recognizing the comprehensive nature of our revisions and the strength of our data, as well as for supporting the publication of this manuscript as she/he states "**The authors have done extensive work addressing my comments, which I greatly appreciate. I also appreciated the clarity and rigour of the rebuttal letter, allowing a detailed assessments of their responses and understanding the logic of their arguments. I found the new data convincing and the changes to the text of the results and discussion compelling. I do not have any further substantial concerns and support the publication of the manuscript."**

The reviewer required one minor clarification which we have addressed as follow**s:**

Comment 1) The only point where I was not sure I understood the argument is the point about the potential interference of the electroplated phospho-mutant TFs with the endogenous TF. The authors state that the CUT&RUN profiles argue against this because they observe reduced binding by the mutant TF. However, unless I missed something, the antibody they use for the CUT&RUN does not distinguish between the electroplated TF and the endogenous TF, or does it? If it does not, then how can they be certain that the reduced binding they observe is not due to reduced binding by the endogenous TF? This does not require new experiments nor re-review. Just a comment in the text one way or the other to clarify this point or acknowledge the potential caveat.

Authors' response: We thank the reviewer for pointing out this lack of clarity in the main text and apologize for the confusion. In our CUT&RUN experiments, the wild-type and phospho-mutant TFs were expressed in the developing mouse brain as fusion proteins containing a FLAG tag and mScarlet, linked via an IRES sequence. Following in utero electroporation, mScarlet-positive cells were isolated by FACS, and **CUT&RUN was performed using a high established and specific anti-FLAG antibody**. Therefore, the CUT&RUN profiles specifically capture the genome-wide binding of the exogenously expressed wild-type or phospho-mutant TFs and do not detect binding of the endogenous TF. Consequently, the reduced binding observed in the phospho-mutant condition reflects decreased chromatin occupancy of the mutant TF itself rather than altered binding of the endogenous protein. To clarify this point, we have revised the text in the Results section accordingly as follows:

"We therefore performed genome-wide binding profiling of wild-type (WT) Nfib and its phospho-deficient mutant (S265A) to map their genomic localization. Both constructs were expressed in the developing cortex via in utero electroporation (IUE) at E13.5 as fusion proteins carrying a FLAG tag and mScarlet (Fig. 6A). At E17.5, mScarlet-positive cells were isolated using fluorescence-activated cell sorting (FACS) and CUT&RUN was subsequently performed using a highly specific anti-FLAG antibody." We thank the reviewer for this suggestion.

Reviewer #4 (Remarks to the Author):

We are grateful to the reviewer for a positive assessment and for confirming that our revised manuscript completely addresses all her/his points as she/he states, "**The author have addressed most of my concerns."**

This reviewer had no further comments**.**
